# Distributed electricity–carbon co-optimization of hydrogen energy storage microgrids with dynamic laplace perturbation and model predictive control

**DOI:** 10.1371/journal.pone.0350884

**Published:** 2026-09-23

**Authors:** Zhongzheng Li, Mengke Liao, Yan Wang, Ruixuan Zhang, Jiawen Sun, Hua Zheng

**Affiliations:** 1 Economic and Technical Research Institute of State Grid Xinjiang Electric Power Co., Ltd., Urumqi, Xinjiang, China; 2 School of Electrical and Electronic Engineering, North China Electric Power University, Beijing, China; Netaji Subhas University of Technology Dwarka, INDIA

## Abstract

Hydrogen energy storage microgrids can improve renewable-energy accommodation and cross-energy flexibility, while repeated boundary-variable exchange in distributed scheduling may expose private operating information. This paper develops a privacy-aware electricity–carbon co-optimization framework that integrates day-ahead distributed clearing with intra-day model predictive control (MPC). The day-ahead model coordinates electricity, heat, hydrogen conversion, battery storage, and thermal storage while accounting for direct gas-boiler emissions and indirect emissions associated with imported electricity. A dynamically decaying Laplace mechanism is incorporated into the alternating direction method of multipliers (ADMM), and privacy performance is evaluated through finite-transcript privacy-budget accounting and reconstruction-based leakage analysis. Compared with fixed Laplace noise, the dynamic mechanism exhibits a higher baseline mean leakage-risk score (0.67 versus 0.58), but reduces the centralized cost gap from 2.85% to 0.94% and the number of iterations from 50 to 38. Matched-condition tests further show lower cost gaps at equal leakage risk and lower leakage risk at equal cost gap. An inverse-variance reconstruction attack reveals increased information exposure during later iterations. Meanwhile, the intra-day MPC maintains feasible multi-energy operation under the tested forecast errors. The base-case operational emissions are 2.09 t CO_2_, and the carbon-parameter sensitivity analysis shows a smooth variation in cleared cost. The results demonstrate the coordinated economic, privacy, and convergence characteristics of the proposed scheduling framework.

## Introduction

The transition toward low-carbon energy systems is increasing the penetration of wind and photovoltaic generation in distribution networks and microgrids, thereby raising the need for flexible resources that can accommodate renewable variability and coordinate multiple energy carriers [1,2]. Hydrogen energy storage is attractive in this context because it can complement electrochemical storage over longer time scales and enable power-to-hydrogen-to-power conversion, although renewable intermittency and forecasting uncertainty remain important scheduling challenges [3–5]. At the same time, carbon-market mechanisms are being incorporated into local energy transactions so that economic scheduling reflects both conventional operating expenditure and emission responsibility [6,7].

Hydrogen-oriented energy-management studies have therefore moved from isolated device models toward coordinated scheduling of electrolyzers, fuel cells, batteries, and renewable generation. Day-ahead hydrogen-microgrid scheduling has explicitly considered photovoltaic and load forecast errors [8], while power-to-gas research has established the role of conversion flexibility in renewable integration [9]. More recently, robust MPC has been used to coordinate hybrid hydrogen–battery systems under uncertain operating conditions [10]. In parallel, low-carbon dispatch formulations have incorporated carbon taxes, trading mechanisms, and emission-allocation rules into system operation [11]. These developments improve energy and carbon coordination, but most rely on centralized or semi-centralized information processing and do not address the information contained in repeatedly exchanged optimization variables.

The cyber layer of distributed energy management has consequently received increasing attention. Blockchain-based trust management and cloud–edge resource coordination provide mechanisms for controlled data exchange and distributed computation [12,13], while secure energy trading schemes use blockchain or cryptographic structures to reduce direct disclosure of participant information [14]. Privacy-preserving learning techniques have also been introduced for smart-grid data processing [15]. From the optimization perspective, consensus-based ADMM has been applied to decentralized scheduling of integrated community energy systems [16], and distributed stochastic and constraint-coupled optimization methods have reduced the need for global information exchange [17,18]. Surveys of smart-grid privacy technologies emphasize that communication transcripts themselves can remain informative even when raw local data are not directly uploaded [19]; recent privacy-preserving peer-to-peer trading further confirms the need to balance information protection against economic performance [20]. Resilience-oriented encryption has also been investigated for cyber-physical energy systems under multiple extreme events [21].

A related stream of work has examined privacy and communication efficiency at the control layer. Reliable privacy-preserving data sharing has been studied for cyber-physical social networks [22], and privacy-aware load control has shown that explicit confidentiality objectives can alter operational decisions [23]. Event-triggered control has also been used to reduce communication burden in microgrid frequency regulation [24]. These studies demonstrate that confidentiality, communication, and control performance are coupled rather than independent design objectives. However, the privacy implications of an iterative day-ahead market-clearing transcript and the way that such a privacy-protected schedule should be transferred to an intra-day controller are still not commonly treated within one framework.

Multi-time-scale scheduling provides a natural mechanism for connecting these layers. Forecast-driven stochastic scheduling can coordinate day-ahead market participation with uncertainty management [25]; day-ahead robust optimization combined with intra-day MPC has been used to update microgrid schedules as new information becomes available [26]; and dynamic prediction horizons have been investigated for integrated-energy dispatch [27]. Theoretical and implementation studies of MPC have further highlighted the importance of closed-loop stability, horizon selection, and physical constraints [28], while recent grid-forming energy-storage optimization illustrates the continuing need to coordinate storage operation with network-level objectives under high renewable penetration [29]. Nevertheless, these multi-time-scale studies generally assume that the day-ahead information passed to the real-time layer is already available without an explicit privacy cost.

Taken together, the literature reveals three unresolved issues. First, electricity–carbon scheduling studies generally quantify economic or emission performance without measuring information leakage from repeated boundary-power releases. Second, privacy-preserving distributed optimization studies rarely evaluate finite-transcript privacy consumption, reconstruction resistance, convergence behavior, and economic optimality loss together in a hydrogen-based multi-energy setting. Third, day-ahead distributed clearing and intra-day MPC are commonly developed as separate modules, so the consistency between a privacy-perturbed reference schedule and a real-time controller is insufficiently characterized. Table 1 summarizes these distinctions at the end of the literature analysis.

**Table 1:** Comparison with representative studies.

Study	Distributed clearing	Privacy mechanism	Electricity– carbon	Hydrogen storage	Day-ahead/ intra-day	Theoretical analysis
Xiang et al. (2024) [7]	Yes	No	Yes	No	No	Bargaining model
Pinnarelli et al. (2026) [8]	No	No	No	Yes	Day-ahead	Forecast-error assessment
Lim et al. (2026) [10]	No	No	No	Yes	Yes	Robust MPC analysis
Ouyang et al. (2024) [11]	No	No	Yes	No	No	No
Jiang et al. (2024) [14]	Yes	Blockchain	No	No	No	Security
Zou and Hu (2026) [20]	Yes	Yes	No	No	No	Privacy
This work	Yes	Dynamic Laplace	Yes	Yes	Yes	Privacy, ADMM, and MPC

To address these gaps, the main contributions are summarized as follows.

1. An electricity–carbon joint clearing model is formulated for a grid-connected hydrogen energy storage microgrid. It coordinates electricity, heat, hydrogen conversion, battery storage, and thermal storage while accounting for both direct gas-boiler emissions and indirect emissions from imported electricity.
2. A privacy-aware ADMM mechanism is developed for the exchanged boundary-power iterates. Finite-transcript differential-privacy budget consumption and reconstruction-based leakage are evaluated separately, and the geometrically decaying perturbation is analyzed in terms of conditional convergence and expected optimality loss.
3. The privacy-protected day-ahead schedule is connected to an intra-day mixed-integer MPC through a single control-reference tracking formulation. Terminal-set and terminal-decrease conditions are used to establish nominal recursive feasibility and nominal closed-loop stability, while bounded mismatch is characterized without claiming robust recursive feasibility for the nominal controller.
4. Numerical tests compare centralized, noise-free, fixed-noise, and dynamically decaying-noise cases under baseline, matched-privacy, matched-cost, and stronger reconstruction conditions. Forecast-error robustness, terminal-set verification, operational emissions, and carbon-parameter sensitivity are additionally evaluated to quantify the practical trade-offs of the complete scheduling framework.

**Fig 1:**
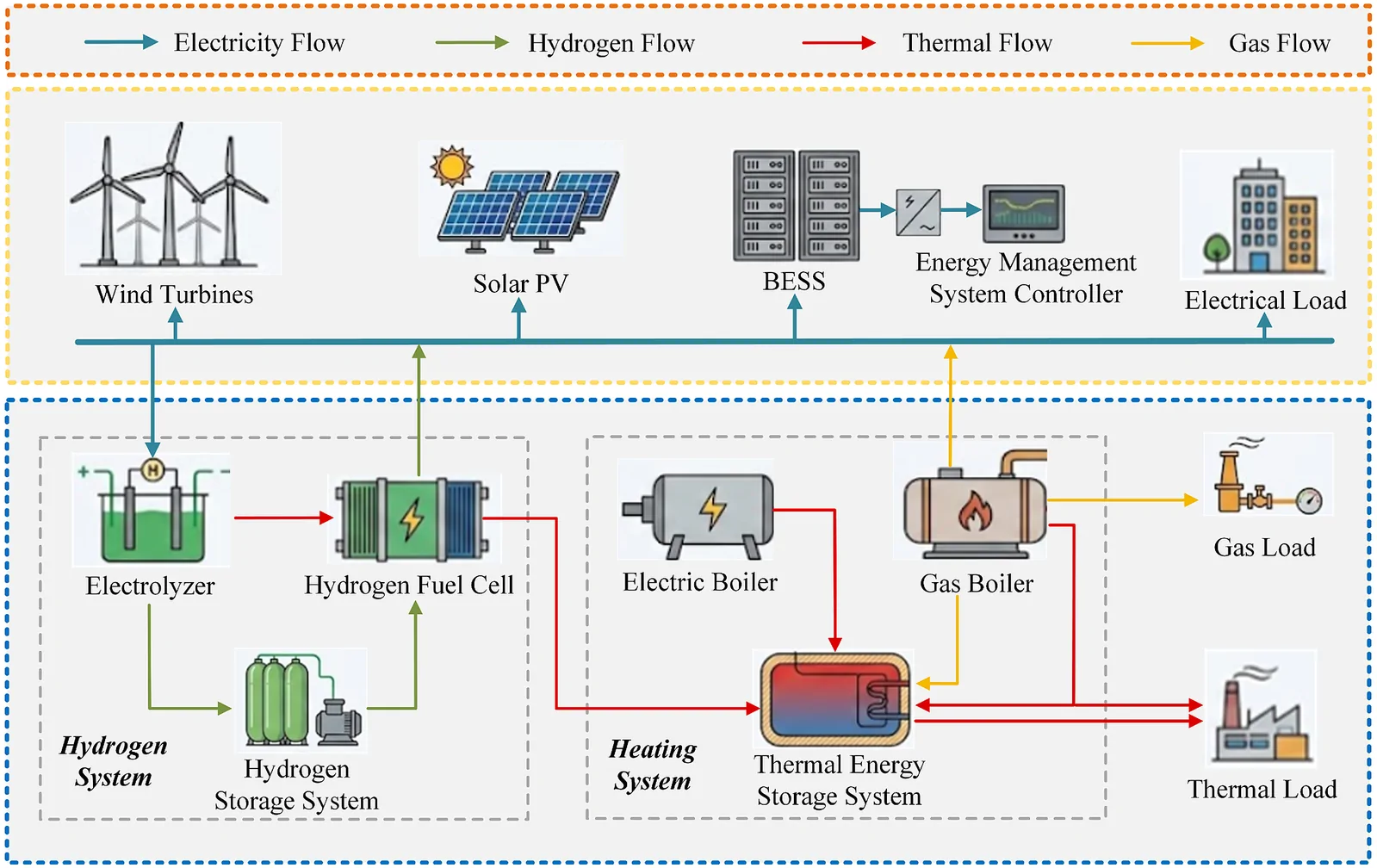
Physical structure and multi-energy flow coupling of the hydrogen energy storage microgrid.

The physical structure and multi-energy coupling relationships of the studied hydrogen energy storage microgrid are illustrated in Fig 1.

## Multi-time-scale electricity–carbon joint clearing model for a hydrogen-based microgrid

As shown in Fig 1, the proposed microgrid coordinates electricity, hydrogen, heat, and gas flows utilizing wind and solar generation, electric and gas boilers, and multi-medium energy storage. To optimize these multi-energy flows, rigorous mathematical models are established for the physical assets, carbon trading mechanism, and multi-time-scale scheduling framework.

### Physical equipment and energy conversion models

The hydrogen energy storage system consists of an electrolyzer and a fuel cell. The electrolyzer consumes electricity to produce hydrogen, accompanied by heat dissipation, while the fuel cell consumes hydrogen to generate electricity and heat. The equipment input/output variables and the separately defined charging and discharging variables are constrained to be non-negative. The tie-line exchange variables $P_{MG}(t)$ and $P_{Grid}(t)$ are exceptions: they are signed variables because the grid-connected microgrid is allowed to import or export electricity. The thermal power outputs of the energy conversion devices are formulated in Eqs (1)–(4).


$$\begin{array}{c} Q_{EB}(t)=\eta_{EB}P_{EB}(t) \end{array}$$ (1)

$$\begin{array}{c} Q_{GB}(t)=\eta_{GB}P_{GB}(t) \end{array}$$ (2)

$$\begin{array}{c} Q_{EL}(t)=\eta_{EL}^{heat}P_{EL}(t) \end{array}$$ (3)

$$\begin{array}{c} Q_{FC}(t)=\eta_{FC}^{heat}P_{FC}(t) \end{array}$$ (4)

where $P_{EB}(t)$ and $P_{GB}(t)$ represent the active electrical power consumed by the electric boiler and the equivalent fuel power consumed by the gas boiler. $Q_{EB}(t)$ and $Q_{GB}(t)$ denote their respective thermal outputs. $\eta_{EB}$ and $\eta_{GB}$ are the heat conversion efficiencies. $P_{EL}(t)$ and $P_{FC}(t)$ denote the electrical power of the electrolyzer and fuel cell, while $\eta_{EL}^{heat}$ and $\eta_{FC}^{heat}$ represent their respective heat recovery coefficients.

The state of charge for the battery, the state of hydrogen charge for the hydrogen system, and the state of heat charge for the thermal storage tank are governed by discrete time dynamic functions, as shown in Eqs (5)–(7).


$$\begin{array}{c} S_{SOC}(t)=S_{SOC}(t-1)+\frac{P_{bat}^{cha}(t)\eta_{bat}^{cha}-P_{bat}^{dis}(t)/\eta_{bat}^{dis}}{E_{bat}^{cap}}\Delta t \end{array}$$ (5)

$$\begin{array}{c} S_{SOHC}(t)=S_{SOHC}(t-1)+\frac{\gamma_{EL}P_{EL}(t)-\gamma_{FC}P_{FC}(t)}{E_{HSS}^{cap}}\Delta t \end{array}$$ (6)

$$\begin{array}{c} S_{HOC}(t)=S_{HOC}(t-1)+\frac{Q_{tst}^{cha}(t)\eta_{tst}^{cha}-Q_{tst}^{dis}(t)/\eta_{tst}^{dis}}{E_{tst}^{cap}}\Delta t \end{array}$$ (7)

where $S_{SOC}(t)$, $S_{SOHC}(t)$, and $S_{HOC}(t)$ represent the normalized states of the three storage systems. $P_{bat}^{cha}(t)$, $P_{bat}^{dis}(t)$, $Q_{tst}^{cha}(t)$, and $Q_{tst}^{dis}(t)$ denote the charging and discharging powers of the battery and thermal storage.

In the hydrogen-storage state equation, $E_{HSS}^{cap}$ is expressed in ${\text{kg-H}}_{2}$. Taking the hydrogen lower heating value as $L_{H_{2}}=33.3$ kWh/${\text{kg-H}}_{2}$ [30], the electrolyzer hydrogen-production efficiency as $\eta_{EL}^{H_{2}}=0.70$, and the fuel-cell electrical efficiency as $\eta_{FC}^{el}=0.55$, the mass-conversion coefficients are calculated as $\gamma_{EL}=\eta_{EL}^{H_{2}}/L_{H_{2}}=0.0210$ ${\text{kg-H}}_{2}$/kWh and $\gamma_{FC}=1/(\eta_{FC}^{el}L_{H_{2}})=0.0546$ ${\text{kg-H}}_{2}$/kWh. Thus, $\gamma_{EL}P_{EL}(t)\Delta t$ represents the hydrogen mass produced by the electrolyzer, whereas $\gamma_{FC}P_{FC}(t)\Delta t$ represents the hydrogen mass consumed by the fuel cell. The selected efficiencies are representative case-study values and provide a dimensionally consistent conversion between electrical energy and stored hydrogen mass. To prevent simultaneous charging and discharging, binary variables are introduced to constrain the operating states. The constraint for the battery is formulated in Eq (8), which is similarly applied to the hydrogen and thermal storage systems.


$$\begin{array}{c} U_{bat}^{cha}(t)+U_{bat}^{dis}(t)+U_{bat}^{static}(t)=1 \end{array}$$ (8)

where $U_{bat}^{cha}(t)$, $U_{bat}^{dis}(t)$, and $U_{bat}^{static}(t)$ are boolean indicators representing the charging, discharging, and static statuses.

### Energy balance and electricity-carbon coupling model

The studied microgrid operates in a grid-connected mode rather than as an electrically isolated system. It performs local energy management independently while retaining a bidirectional tie-line connection to the upper-level grid. The electrical and thermal power balance constraints are formulated in Eqs (9) and (10).


$$\begin{array}{c} P_{MG}(t)+P_{W}(t)+P_{PV}(t)+P_{FC}(t)+P_{bat}^{dis}(t)=P_{load}(t)+P_{EL}(t)+P_{bat}^{cha}(t)+P_{EB}(t) \end{array}$$ (9)

$$\begin{array}{c} Q_{GB}(t)+Q_{EB}(t)+Q_{tst}^{dis}(t)+Q_{EL}(t)+Q_{FC}(t)=Q_{load}(t)+Q_{tst}^{cha}(t) \end{array}$$ (10)

where $P_{W}(t)$ and $P_{PV}(t)$ denote the renewable generation from wind and solar power, while $P_{load}(t)$ and $Q_{load}(t)$ are the electrical and thermal demands.

The tie-line power $P_{MG}(t)$ is a signed net-exchange variable. Its import and export components are defined as


$$\begin{array}{cc} P_{MG}(t) & =P_{MG}^{imp}(t)-P_{MG}^{exp}(t),\\ P_{MG}^{imp}(t) & =\max\{P_{MG}(t),0\},\;P_{MG}^{exp}(t)=\max\{-P_{MG}(t),0\},\\ -P_{MG}^{exp,max} & \leq P_{MG}(t)\leq P_{MG}^{imp,max}. \end{array}$$ (11)

Accordingly, $P_{MG}(t)>0$ indicates that the microgrid imports electricity from the upper-level grid, $P_{MG}(t)<0$ indicates that surplus electricity is exported, and $P_{MG}(t)=0$ indicates no tie-line exchange at that time. Therefore, export is explicitly allowed within the tie-line capacity limit. Because $P_{MG}^{imp}(t)$ and $P_{MG}^{exp}(t)$ are derived from the same signed variable $P_{MG}(t)$ through the positive- and negative-part definitions in Eq (11), they are mutually exclusive by construction and are not optimized as two independent simultaneous exchange variables. All device-generation, consumption, charging, and discharging variables remain non-negative; only the net boundary-exchange variable uses a signed convention.

The carbon emission trading mechanism accounts for both direct on-site emissions and indirect emissions associated with electricity imported from the upper-level grid. The baseline carbon quota is allocated according to the equivalent energy supplied to the electrical and thermal loads. Using the sign convention in Eq (11), only the non-negative import component $P_{MG}^{imp}(t)$ is assigned a grid-emission term; the export component is not treated as a negative-emission credit. The carbon trading cost is formulated in Eq (12).


$$\begin{array}{cc} C_{CO2}=\lambda_{CO2}\sum_{t=1}^{T}[ & \mu_{GB}P_{GB}(t)+\mu_{Grid}(t)P_{MG}^{imp}(t)\\ & -\mu_{quota}(P_{load}(t)+Q_{load}(t))]\Delta t \end{array}$$ (12)

where $C_{CO2}$ is the total carbon trading cost evaluated over the scheduling cycle, $\lambda_{CO2}$ is the carbon trading price, $\mu_{GB}$ is the direct emission intensity of the gas boiler, and $\mu_{Grid}(t)$ is the emission intensity of imported grid electricity. $\mu_{quota}$ denotes the baseline quota allocation coefficient. The formulation permits a time-varying grid emission factor; a constant value is adopted in the base case for reproducibility. Only positive grid imports are assigned indirect emissions, whereas exported electricity is not credited with negative grid emissions. The numerical natural-gas emission factor used in the case study follows the IPCC stationary-combustion guidance [31]; its unit conversion is reported with the simulation parameters.

For the numerical evaluation, the physical operational emissions are reported separately from the monetary carbon-trading term. The total direct and indirect operational emissions over the scheduling horizon are defined as


$$\begin{array}{c} E_{CO_{2}}^{op}=\sum_{t=1}^{T}\left[\mu_{GB}P_{GB}(t)+\mu_{Grid}(t)P_{MG}^{imp}(t)\right]\Delta t. \end{array}$$ (13)

This quantity excludes the quota term because the quota changes the settlement cost but does not represent a physical negative emission. The base-case value and the sensitivity to the grid-import emission factor are evaluated in the simulation section.

### Multi-time-scale optimization objectives

The proposed dispatch framework consists of a day-ahead global scheduling stage and an intra-day real-time scheduling stage. In the day-ahead stage, the objective function aims to minimize the comprehensive operation cost, encompassing the grid transaction cost, operation and maintenance cost, fuel cost, and carbon trading cost. The complete cost formulation and the throughput definitions used in the maintenance term are given in Eqs (14)–(18).


$$\begin{array}{c} \min F_{DA}=C_{grid}+C_{OM}+C_{fuel}+C_{CO2} \end{array}$$ (14)

$$\begin{array}{c} C_{grid}=\sum_{t=1}^{T}\lambda_{grid}(t)P_{MG}(t)\Delta t \end{array}$$ (15)

Under the signed convention of Eq (11), a positive term in Eq (15) is an import payment, whereas a negative term represents export revenue. The base case adopts the same time-of-use settlement price $\lambda_{grid}(t)$ for both directions to maintain a consistent bidirectional settlement rule.


$$\begin{array}{c} C_{OM}=\sum_{t=1}^{T}\left(k_{W}P_{W}(t)+k_{PV}P_{PV}(t)+k_{bat}P_{bat}^{abs}(t)+k_{hss}P_{hss}^{abs}(t)\right)\Delta t \end{array}$$ (16)

$$\begin{array}{c} C_{fuel}=\sum_{t=1}^{T}\lambda_{gas}(t)P_{GB}(t)\Delta t, \end{array}$$ (17)

where $\lambda_{gas}(t)$ is the natural-gas price in CNY/${\text{kWh}}_{fuel}$ and $P_{GB}(t)$ is the gas-boiler fuel-input power. In the base case, $\lambda_{gas}(t)$ is constant over the scheduling horizon. The absolute battery and hydrogen-system throughputs appearing in Eq (16) are defined as


$$\begin{array}{c} P_{bat}^{abs}(t)=P_{bat}^{cha}(t)+P_{bat}^{dis}(t),\;P_{hss}^{abs}(t)=P_{EL}(t)+P_{FC}(t). \end{array}$$ (18)

These definitions charge operation-and-maintenance costs according to the total energy processed by the bidirectional storage devices and avoid ambiguity associated with signed net power.

where $F_{DA}$ represents the day-ahead objective function. $C_{grid}$ is the electricity transaction cost with the main grid, and $\lambda_{grid}(t)$ is the time-of-use electricity price at time slot *t*. $C_{OM}$ and $C_{fuel}$ denote the maintenance and natural-gas purchase costs, respectively. $k_{W}$, $k_{PV}$, $k_{bat}$, and $k_{hss}$ represent the respective unit maintenance penal*t*y coefficients.

In the intra-day real-time scheduling stage, model predictive control is used to correct short-term forecast deviations around the day-ahead baseline. The intra-day MPC adopts control-sequence tracking based on the day-ahead control reference. The day-ahead control reference corresponding to the control vector in Eq (52) is defined as


$$\begin{array}{cc} u^{ref}(k)=[ & P_{bat,DA}^{cha}(k),\;P_{bat,DA}^{dis}(k),\;P_{EL,DA}(k),\;P_{FC,DA}(k),\\ & Q_{tst,DA}^{cha}(k),\;Q_{tst,DA}^{dis}(k),\;P_{EB,DA}(k),\;P_{GB,DA}(k){]}^{T}. \end{array}$$ (19)

where the subscript DA denotes the value obtained from the day-ahead clearing stage. The storage states are not tracked by a separate stage-wise state objective. Instead, they evolve through the state-space model and enter the single rolling objective only through a terminal-state penalty. The complete and unique intra-day objective is stated in Eq (63).

The resulting objective and the mixed-integer physical constraints can be written in the canonical mixed-integer quadratic programming form in Eq (20). This canonical form is a solver representation of the same rolling objective and is not an additional tracking formulation.


$$\begin{array}{c} \left\{ \begin{array}{c} \min\frac{1}{2}x^{T}Qx+c^{T}x\\ s.t.\;Ax\leq b\\ \;x_{j}\in \{0,1\},\forall j\in J \end{array}\right. \end{array}$$ (20)

**Fig 2:**
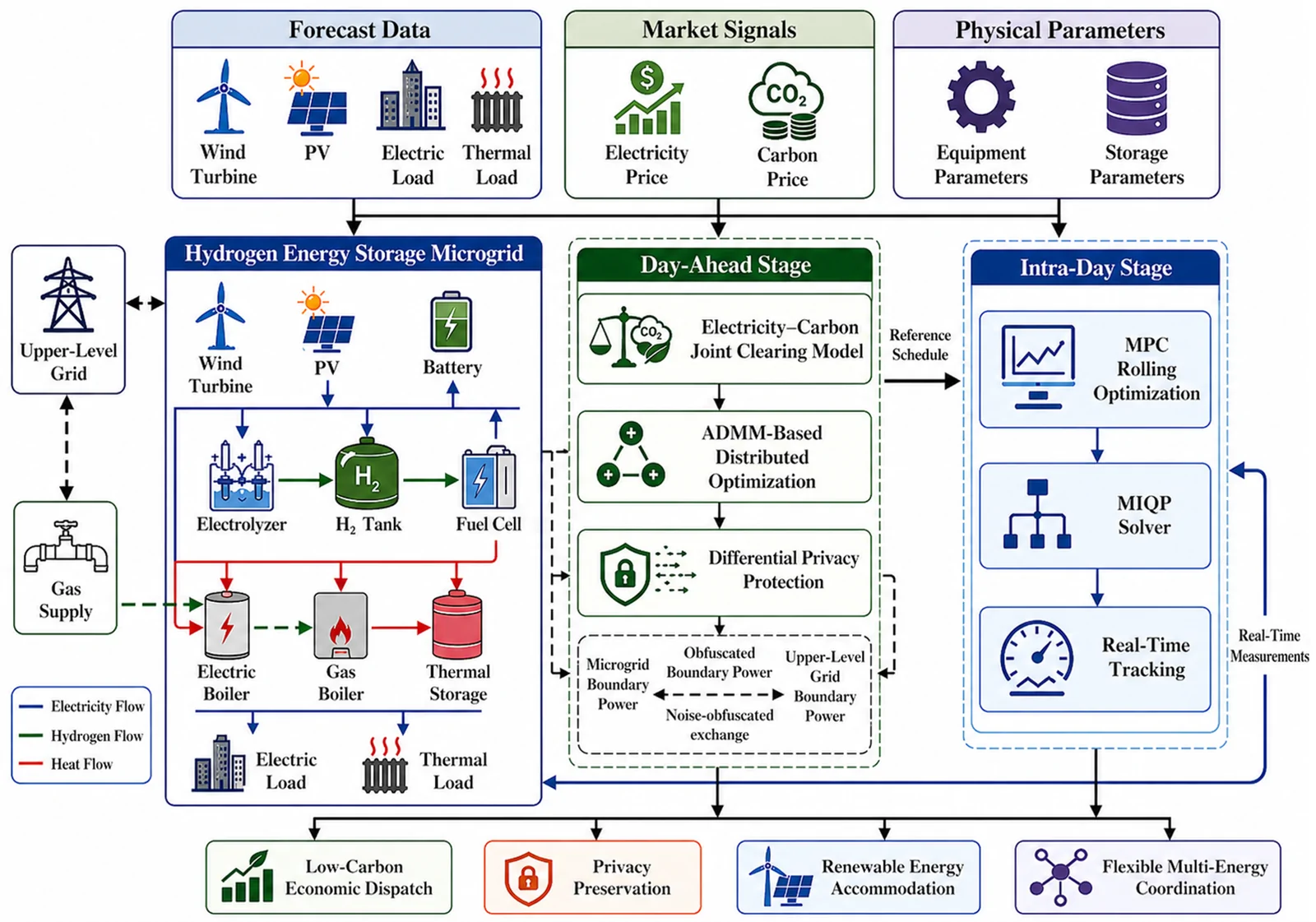
Overall framework of the proposed privacy-preserving multi-time-scale scheduling strategy.

Here, *Q* and *c* are the quadratic and linear coefficients obtained from the single rolling objective in Eq (63). *A* and *b* collect the linear physical-boundary and energy-balance constraints, and $x_{j}$ denotes the binary equipment-state variable. Therefore, Eq (20) changes only the algebraic representation used by the solver and does not define a second tracking criterion. Based on the electricity-carbon joint clearing model and the intra-day rolling optimization formulation, the overall framework of the proposed privacy-preserving multi-time-scale scheduling strategy is illustrated in Fig 2. The framework consists of the hydrogen energy storage microgrid, the day-ahead privacy-preserving distributed clearing stage, and the intra-day MPC-based rolling tracking stage.

## Privacy-preserving distributed optimization based on the alternating direction method of multipliers

The day-ahead scheduling model established in the previous section involves a large number of physical constraints and sensitive operational parameters of the microgrid. Submitting these parameters to a centralized energy management system increases the risk of commercial data disclosure. Therefore this section proposes a distributed optimization algorithm combining the alternating direction method of multipliers and differential privacy to decouple the global scheduling problem.

### Standard distributed decoupling framework

The physical coupling boundary between the microgrid and the upper-level main grid is the tie line active power. Let $P_{MG}(t)$ denote the expected boundary exchange power optimized by the internal energy management system of the microgrid. Let $P_{Grid}(t)$ denote the expected tie line power scheduled by the upper-level grid operator. Both variables follow the sign convention of Eq (11): positive values denote power delivered from the upper-level grid to the microgrid, and negative values denote power exported by the microgrid. To achieve global power balance, the boundary coupling constraint is formulated in Eq (21).


$$\begin{array}{c} P_{MG}(t)=P_{Grid}(t) \end{array}$$ (21)

The global objective function is decoupled into the independent operational cost of the microgrid and the power supply cost of the main grid. By introducing an augmented Lagrangian penalty term, the unconstrained augmented Lagrangian function of the system is constructed as shown in Eq (22).


$$\begin{array}{c} L(x,y,\lambda )=F_{MG}(x)+F_{Grid}(y)+\sum_{t=1}^{T}\lambda (t)\left(P_{MG}(t)-P_{Grid}(t)\right)+\frac{\rho }{2}\sum_{t=1}^{T}{\left\|P_{MG}(t)-P_{Grid}(t)\right\|}_{2}^{2} \end{array}$$ (22)

where *x* represents the set of all internal decision variables of the microgrid. *y* represents the set of decision variables of the main grid. $F_{MG}(x)$ and $F_{Grid}(y)$ denote their respective local objective functions. λ(t) is the dual multiplier associated with the boundary power consensus constraint. ρ is the positive constant penalty parameter. The numerical value used in all reported ADMM experiments is ρ=1.00, as summarized together with the stopping tolerances in Table 4. During the iterative solving process, the microgrid and the main grid alternately update their own decision variables. In the *k* + 1 iteration step, the microgrid local optimization problem is updated by minimizing the Lagrangian function with the fixed variables from the *k* iteration, as formulated in Eq (23).


$$\begin{array}{c} x^{k+1}=\text{arg}\min_{x}\left(F_{MG}(x)+\sum_{t=1}^{T}\lambda^{k}(t)P_{MG}(t)+\frac{\rho }{2}\sum_{t=1}^{T}{\left\|P_{MG}(t)-P_{Grid}^{k}(t)\right\|}_{2}^{2}\right) \end{array}$$ (23)

Subsequently, the main grid updates its local decision variables based on the latest boundary power boundary information received from the microgrid, as shown in Eq (24).


$$\begin{array}{c} y^{k+1}=\text{arg}\min_{y}\left(F_{Grid}(y)-\sum_{t=1}^{T}\lambda^{k}(t)P_{Grid}(t)+\frac{\rho }{2}\sum_{t=1}^{T}{\left\|P_{MG}^{k+1}(t)-P_{Grid}(t)\right\|}_{2}^{2}\right) \end{array}$$ (24)

Finally, the dual multiplier is updated through the gradient ascent method according to the primal residual, as shown in Eq (25).


$$\begin{array}{c} \lambda^{k+1}(t)=\lambda^{k}(t)+\rho \left(P_{MG}^{k+1}(t)-P_{Grid}^{k+1}(t)\right) \end{array}$$ (25)

### Differential privacy noise mechanism

Although standard distributed frameworks avoid sharing explicit parameters, adversaries can still infer microgrid internals by analyzing the continuous trajectory of the exchanged boundary power $P_{MG}(t)$ across iterations. A differential privacy mechanism is therefore integrated into the information interaction process to provide a quantifiable privacy guarantee for each released iterate and for the finite communication transcript. For two adjacent datasets *D* and $D^{\prime }$ differing by one element, a randomized algorithm *M* satisfies ϵ differential privacy if the probability ratio of outputting any identical result set *S* is bounded as defined in Eq (26).


$$\begin{array}{c} Pr[M(D)\in S]\leq \exp(\epsilon )\times Pr[M(D^{\prime })\in S] \end{array}$$ (26)

where ϵ is the privacy budget parameter defining the privacy protection level. A smaller ϵ indicates stronger privacy preservation but introduces larger numerical perturbations. In the dynamically decaying mechanism below, $\epsilon_{0}$ is used as the baseline calibration coefficient, whereas the actual privacy budget of the *k*th released iterate is denoted by $\epsilon_{k}$. To calibrate the required noise magnitude, the *L*_1_ global sensitivity of the exchanged power sequence is calculated. The mathematical definition of the global sensitivity Δf is formulated in Eq (27).


$$\begin{array}{c} \Delta f=\max_{D,D^{\prime }}{\left\|f(D)-f(D^{\prime })\right\|}_{1} \end{array}$$ (27)

The Laplace mechanism is utilized to generate the random noise sequence. The probability density function of the zero mean Laplace distribution is shown in Eq (28).


$$\begin{array}{c} p(z)=\frac{1}{2b}\exp\left(-\frac{\left|z\right|}{b}\right) \end{array}$$ (28)

where *z* is the random variable and *b* is the scale parameter determined by the global sensitivity and the privacy budget.

### Eavesdropping attack model and privacy leakage metric

The privacy evaluation considers a passive data-eavesdropping attack on the communication link between the microgrid and the upper-level grid. The eavesdropper can continuously intercept the boundary-power messages transmitted during the ADMM iterations and is assumed to know the public communication protocol and iteration index. However, the eavesdropper cannot access the local objective functions, equipment operating states, private load data, or the realizations of the injected Laplace noise. The attack is passive; therefore, the intercepted packets are used only for inference and are neither modified nor blocked. For operating day *d*, time period *t*, and ADMM iteration *k*, the intercepted value is expressed as


$$\begin{array}{c} z_{d}^{k}(t)=\left\{ \begin{array}{cc} {\tilde{P}}_{MG,d}^{k}(t), & \text{with the proposed privacy-preserving strategy},\\ P_{MG,d}^{k}(t), & \text{without privacy-preserving protection}. \end{array}\right. \end{array}$$ (29)

The eavesdropper reconstructs the unperturbed converged boundary power by applying a weighted least-squares estimator to the complete intercepted iteration trajectory. Later iterations receive larger weights because they contain more information about the converged scheduling result. The reconstructed value is defined as


$$\begin{array}{c} {\hat{P}}_{MG,d}^{atk}(t)=\text{arg}\min_{p}\sum_{k=1}^{K_{d}}w_{d,k}{\left[z_{d}^{k}(t)-p\right]}^{2}=\sum_{k=1}^{K_{d}}w_{d,k}z_{d}^{k}(t),\;w_{d,k}=\frac{2k}{K_{d}(K_{d}+1)}, \end{array}$$ (30)

where $K_{d}$ is the total number of ADMM iterations on day *d*, $w_{d,k}\geq 0$, and $\sum_{k=1}^{K_{d}}w_{d,k}=1$. The unperturbed converged boundary power $P_{MG,d}^{⋆}(t)$ is retained only as the offline ground truth for performance evaluation and is not available to the eavesdropper.

Because the dynamically decaying mechanism produces iteration-dependent noise variance, a second reconstruction rule is included as a stronger attack model. For independent Laplace perturbations, $\mathrm{Var}[\theta^{k}(t)]=2b_{k}^{2}$, and the inverse-variance weights are therefore


$$\begin{array}{c} w_{d,k}^{IV}=\frac{b_{k}^{-2}}{\sum_{j=1}^{K_{d}}b_{j}^{-2}},\;{\hat{P}}_{MG,d}^{IV}(t)=\sum_{k=1}^{K_{d}}w_{d,k}^{IV}z_{d}^{k}(t). \end{array}$$ (31)

This estimator assigns greater weight to late iterations with smaller $b_{k}$ and therefore provides a stricter reconstruction test for the decaying-noise trajectory. For a fixed-noise sequence, all $b_{k}$ are equal and Eq (31) reduces to uniform weighting. The leakage metrics in Eqs (32)–(36) are evaluated for both the original linear-weight estimator and the inverse-variance estimator. The normalized reconstruction error and the corresponding privacy leakage risk are calculated as


$$\begin{array}{c} E_{d,t}=\min\left\{\frac{\left|{\hat{P}}_{MG,d}^{atk}(t)-P_{MG,d}^{⋆}(t)\right|}{P_{MG}^{\max}-P_{MG}^{\min}+\delta },\;1\right\}, \end{array}$$ (32)

$$\begin{array}{c} R_{d,t}=1-E_{d,t},\;0\leq R_{d,t}\leq 1, \end{array}$$ (33)

where $P_{MG}^{\max}$ and $P_{MG}^{\min}$ are the maximum and minimum unperturbed boundary-power values in the evaluation dataset, and δ is a small positive constant used to avoid division by zero. A larger $R_{d,t}$ indicates a smaller reconstruction error and, consequently, a higher reconstruction success level for the unperturbed boundary-power profile from the intercepted data. Thus, the metric evaluates reconstruction-based leakage under the specified eavesdropping attack rather than directly replacing the theoretical differential-privacy budget ϵ.

For $N_{d}$ operating days and *T* time periods per day, the mean privacy leakage risk is defined as


$$\begin{array}{c} \bar{R}=\frac{1}{N_{d}T}\sum_{d=1}^{N_{d}}\sum_{t=1}^{T}R_{d,t}. \end{array}$$ (34)

The remaining descriptive statistics reported in the privacy comparison table are calculated as


$$\begin{array}{c} R_{\max}=\max_{d,t}R_{d,t},\;R_{\min}=\min_{d,t}R_{d,t},\;\sigma_{R}=\sqrt{\frac{1}{N_{d}T-1}\sum_{d=1}^{N_{d}}\sum_{t=1}^{T}{\left(R_{d,t}-\bar{R}\right)}^{2}}. \end{array}$$ (35)

The proportions of high-, medium-, and low-risk periods are obtained from


$$\begin{array}{c} \eta_{c}=\frac{1}{N_{d}T}\sum_{d=1}^{N_{d}}\sum_{t=1}^{T}𝕀\;\left(R_{d,t}\in {ℐ}_{c}\right)\times 100\%,\;c\in \{H,M,L\}, \end{array}$$ (36)

where ${ℐ}_{H}=(0.80,1]$, ${ℐ}_{M}=[0.50,0.80]$, and ${ℐ}_{L}=[0,0.50)$ denote the high-, medium-, and low-risk intervals, respectively, and 𝕀(·) is the indicator function.

### Execution process of the privacy-preserving algorithm

In the proposed privacy-preserving optimization strategy, the microgrid does not transmit the precise boundary power $P_{MG}^{k+1}(t)$ directly to the main grid. Instead, a dynamic Laplace noise sequence is superimposed onto the precise power to generate a confusing output sequence, as formulated in Eq (37).


$$\begin{array}{c} {\tilde{P}}_{MG}^{k+1}(t)=P_{MG}^{k+1}(t)+\theta^{k+1}(t) \end{array}$$ (37)

where ${\tilde{P}}_{MG}^{k+1}(t)$ is the noise obfuscated boundary power. $\theta^{k+1}(t)$ is the dynamic noise value added at the *k* + 1 iteration step.

To reduce the influence of perturbation on the numerical residuals, the noise scale is decreased over a prescribed finite iteration horizon. The dynamic scale parameter at iteration *k* is defined as


$$\begin{array}{c} b_{k}=c\alpha^{k}\frac{\Delta f}{\epsilon_{0}},\;0<\alpha <1,\;1\leq k\leq K_{\max}, \end{array}$$ (38)

where *c* is the initialization coefficient, α is the decay factor, $\epsilon_{0}$ is the baseline privacy coefficient, and $K_{\max}$ is the prescribed maximum number of released ADMM iterates. Because $K_{\max}$ is finite, $b_{k}$ remains positive for every transmitted iterate. Moreover, the unperturbed boundary power is not additionally released after termination through the monitored communication channel.

For the Laplace mechanism, the privacy budget associated with the *k*th released boundary-power vector is


$$\begin{array}{c} \epsilon_{k}=\frac{\Delta f}{b_{k}}=\frac{\epsilon_{0}}{c\alpha^{k}}. \end{array}$$ (39)

According to the basic sequential composition property, if the algorithm terminates after *K* released iterates, the complete intercepted transcript satisfies an upper-bound privacy budget of


$$\begin{array}{c} \epsilon_{cum}(K)=\sum_{k=1}^{K}\epsilon_{k}=\frac{\epsilon_{0}}{c}\sum_{k=1}^{K}\alpha^{-k}=\frac{\epsilon_{0}}{c}\frac{\alpha^{-K}-1}{1-\alpha },\;K\leq K_{\max}. \end{array}$$ (40)

Equations (39) and (40) also make the privacy–accuracy trade-off explicit. Since $b_{k+1}/b_{k}=\alpha$ and $\epsilon_{k+1}/\epsilon_{k}=1/\alpha >1$, later releases receive weaker perturbation than earlier releases. Therefore, the proposed scheme does not claim uniform or zero-leakage protection at convergence; it provides a finite, explicitly reported privacy bound for the released finite transcript. In particular, differences between two successive late-stage iterates may reveal more trajectory information than differences between early-stage iterates, which is a limitation of the present geometrically decaying-noise design. Following the reception of the obfuscated power information, the main grid and the multiplier updating step utilize the modified values. The updated dual multiplier calculation is formulated in Eq (41).


$$\begin{array}{c} \lambda^{k+1}(t)=\lambda^{k}(t)+\rho \left({\tilde{P}}_{MG}^{k+1}(t)-P_{Grid}^{k+1}(t)\right) \end{array}$$ (41)

The alternating iterations are executed under the finite horizon $K_{\max}$ and terminate when both the primal residual $r^{k+1}$ and the dual residual $s^{k+1}$ fall below their predefined tolerances, or when the maximum iteration number is reached. Accordingly, convergence is interpreted as practical numerical convergence within the prescribed tolerances rather than exact disclosure-free convergence to an unperturbed terminal message. The finite horizon also bounds the cumulative privacy loss by $\epsilon_{cum}(K_{\max})$. The definitions of the residuals are shown in Eqs (42) and (43).

For a compact residual definition, let ${\tilde{𝐏}}_{MG}^{k}=[{\tilde{P}}_{MG}^{k}(1),...,{\tilde{P}}_{MG}^{k}(T){]}^{T}$ and ${𝐏}_{Grid}^{k}=[P_{Grid}^{k}(1),...,P_{Grid}^{k}(T){]}^{T}$. The primal and dual residuals used in the stopping test are defined as


$$\begin{array}{c} r^{k+1}={\left\|{\tilde{𝐏}}_{MG}^{k+1}-{𝐏}_{Grid}^{k+1}\right\|}_{2}, \end{array}$$ (42)

$$\begin{array}{c} s^{k+1}=\rho {\left\|{𝐏}_{Grid}^{k+1}-{𝐏}_{Grid}^{k}\right\|}_{2}. \end{array}$$ (43)

Because the local scheduling model contains the binary operating-state variables in Eq (8), its feasible region is nonconvex. Consequently, the standard global-convergence result for convex ADMM cannot be directly extended to the complete mixed-integer problem. In this study, every local mixed-integer subproblem is solved to solver optimality by CPLEX at each iteration, and convergence is assessed numerically using Eqs (42) and (43). Therefore, “convergence” in the following simulation analysis denotes finite-iteration numerical convergence under the specified residual tolerances, rather than a general proof of global convergence for arbitrary mixed-integer instances.

### Theoretical privacy, convergence, and optimality properties

The following results distinguish the finite-transcript privacy guarantee from the convergence and economic-accuracy properties of the perturbed iterations. Let $\theta^{k}=[\theta^{k}(1),...,\theta^{k}(T){]}^{T}$ and let $b_{k}=b_{0}\alpha^{k}$, where $b_{0}=c\Delta f/\epsilon_{0}$.

**Assumption 1** *For any fixed feasible binary operating-mode sequence, the remaining microgrid and upper-level-grid subproblems have nonempty compact feasible sets; their objective functions are proper, closed, and convex; and the boundary-consensus problem admits a saddle point. Each local continuous subproblem is solved exactly. The components of* $\theta^{k}$ *are mutually independent and satisfy* $\theta^{k}(t)\sim \mathrm{Lap}(0,b_{k})$ *with* 0<α<1*.*

**Proposition 1** (Finite-transcript privacy-budget accounting*). For the adjacency relation in* Eq (27), *the kth released vector* ${\tilde{P}}_{MG}^{k}$ *satisfies* $\epsilon_{k}$*-differential privacy with* $\epsilon_{k}=\Delta f/b_{k}$*. A transcript containing K released vectors satisfies* $\epsilon_{cum}(K)$*-differential privacy under basic sequential composition, where* $\epsilon_{cum}(K)$ *is given by* Eq (40).

*Proof.* For adjacent datasets *D* and $D^{\prime }$, the ratio of the joint Laplace densities of one released vector is bounded by


$$\begin{array}{c} \frac{p({\tilde{P}}_{MG}^{k}|D)}{p({\tilde{P}}_{MG}^{k}|D^{\prime })}\leq \exp\;\left(\frac{\|f(D)-f(D^{\prime }){\|}_{1}}{b_{k}}\right)\leq \exp\;\left(\frac{\Delta f}{b_{k}}\right)=\exp(\epsilon_{k}). \end{array}$$ (44)

Thus, each release is $\epsilon_{k}$-differentially private. Sequential composition over the *K* transmitted iterates gives $\sum_{k=1}^{K}\epsilon_{k}=\epsilon_{cum}(K)$ [32].

Proposition 1 is used to account for the formal privacy budget consumed by the finite transcript under the stated adjacency relation. It is not used as a stand-alone claim of strong practical protection. In the reported run, $\epsilon_{cum}(38)=60.23$ under basic composition, which is a large cumulative budget. The manuscript therefore reports this value explicitly and evaluates practical reconstruction resistance separately through $R_{d,t}$ under the stated attack model. □

**Theorem 1** (Conditional convergence under summable perturbations). *Under Assumption 1, the perturbed ADMM iterates for any fixed feasible binary mode sequence converge almost surely to a primal–dual solution of the associated convex problem, and the corresponding primal and dual residuals converge to zero.*

*Proof.* For a scalar zero-mean Laplace variable, $𝔼|\theta^{k}(t)|=b_{k}$. Hence,


$$\begin{array}{c} \sum_{k=1}^{\infty }𝔼\|\theta^{k}{\|}_{1}=T\sum_{k=1}^{\infty }b_{0}\alpha^{k}=\frac{Tb_{0}\alpha }{1-\alpha }<\infty . \end{array}$$ (45)

Because the expected sum of the non-negative random variables is finite, $\sum_{k=1}^{\infty }\|\theta^{k}{\|}_{1}<\infty$ almost surely. The noisy boundary update can therefore be interpreted as an inexact ADMM iteration with an absolutely summable error sequence. Standard inexact-ADMM results then imply convergence to a saddle point and $r^{k}\to 0$, $s^{k}\to 0$ for the fixed-mode convex problem [33,34]. □

**Proposition 2** (Conditional expected terminal optimality-loss bound). *Let V(p) be the optimal value of the coupled clearing problem parameterized by the boundary-power vector p, and suppose that V is Lipschitz continuous with constant* $L_{V}$ *on the compact tie-line feasible set. Let* ${\bar{p}}^{K}$ *denote the terminal unperturbed boundary iterate generated by the distributed algorithm and assume that its deviation from the centralized optimum satisfies*


$$\begin{array}{c} {\left\|{\bar{p}}^{K}-p^{⋆}\right\|}_{2}\leq \delta_{K}. \end{array}$$ (46)

For the released terminal vector ${\hat{p}}^{K}={\bar{p}}^{K}+\theta^{K}$, the expected cost deviation satisfies


$$\begin{array}{c} 𝔼\left|V({\hat{p}}^{K})-V(p^{⋆})\right|\leq L_{V}\left(\delta_{K}+\sqrt{2T}\;b_{K}\right). \end{array}$$ (47)

*Proof.* The Lipschitz property and the triangle inequality yield


$$\begin{array}{c} \left|V({\hat{p}}^{K})-V(p^{⋆})\right|\leq L_{V}\left({\left\|{\bar{p}}^{K}-p^{⋆}\right\|}_{2}+{\left\|\theta^{K}\right\|}_{2}\right). \end{array}$$ (48)

Using Eq (46) and $𝔼\|\theta^{K}{\|}_{2}^{2}=2Tb_{K}^{2}$ for independent Laplace components, Jensen’s inequality gives $𝔼\|\theta^{K}{\|}_{2}\leq \sqrt{2T}b_{K}$, which establishes Eq (47). □

The bound $\delta_{K}$ represents the distance between the terminal unperturbed distributed iterate and the centralized optimum. It is not identified with the primal residual, because a small consensus residual alone does not guarantee proximity to the centralized optimum. When a problem-specific error-bound relation of the form $\|{\bar{p}}^{K}-p^{⋆}{\|}_{2}\leq \kappa_{r}r^{K}+\kappa_{s}s^{K}$ is available, $\delta_{K}$ may be replaced by the corresponding residual-based bound. Otherwise, the realized economic deviation is evaluated directly against the centralized benchmark using Eq (49).

**Remark 1** *Theorem 1 is conditional because binary charging, discharging, and conversion modes make the complete scheduling problem nonconvex. It characterizes the continuous convex subproblem associated with a fixed feasible mode sequence, while the full mixed-integer implementation is evaluated through the reported residuals and benchmark cost gaps. Proposition 2 is also conditional on the explicit terminal-solution error bound* $\delta_{K}$*; the primal residual is used only as a consensus stopping indicator and is not treated as a direct measure of distance to the centralized optimum. Proposition 1 shows that convergence accuracy and privacy cannot both improve indefinitely: geometric noise decay makes the perturbation summable but increases the per-release and cumulative privacy budgets. The proposed implementation therefore uses a finite stopping horizon and reports both* $\epsilon_{cum}(K)$ *and the realized cleared-cost gap.*

### Performance evaluation metrics

To quantify the realized economic deviation of each distributed method from the centralized benchmark, let $F_{cen}$ denote the centralized cleared cost, $F_{m}$ the cleared cost of method *m*, and $F_{ADMM}$ the cleared cost of the noise-free distributed ADMM. The centralized optimality gap $G_{m}$ and the additional noise-induced loss $L_{m}^{noise}$ are defined as


$$\begin{array}{c} G_{m}=\frac{F_{m}-F_{cen}}{F_{cen}}\times 100\%,\;L_{m}^{noise}=\frac{F_{m}-F_{ADMM}}{F_{ADMM}}\times 100\%. \end{array}$$ (49)

The first metric measures the overall departure from centralized clearing, whereas the second isolates the incremental economic effect introduced by the privacy perturbation relative to the same distributed algorithm without noise.

The fixed-noise benchmark is additionally evaluated under matched-privacy and matched-cost conditions rather than only by matching the average perturbation amplitude. A parameter sweep is performed over the fixed Laplace scale and over the dynamic-noise calibration parameters. Each configuration is mapped to the pair $\left(\bar{R},G_{m}\right)$ together with its iteration count and computation time. A configuration is identified as Pareto efficient if no other tested configuration has both a lower mean reconstruction-risk score and a lower centralized cost gap. For the matched-privacy comparison, configurations with approximately equal $\bar{R}$ are compared in terms of $G_{m}$, iteration count, and computation time; for the matched-cost comparison, configurations with approximately equal $G_{m}$ are compared in terms of $\bar{R}$. This procedure avoids treating equal average noise amplitude as equivalent privacy or equivalent economic burden. For the multidimensional comparison of privacy protection, economic optimality, convergence speed, solution stability, and computational efficiency, lower-is-better raw indicators are converted into larger-is-better normalized scores. For method *m* and indicator *j*, the score is


$$\begin{array}{c} q_{m,j}=0.20+0.80\frac{x_{j}^{\max}-x_{m,j}}{x_{j}^{\max}-x_{j}^{\min}}, \end{array}$$ (50)

where $x_{m,j}$ is the raw indicator value, and $x_{j}^{\max}$ and $x_{j}^{\min}$ are the maximum and minimum values among the compared methods. The offset 0.20 prevents the lowest score from collapsing at the radar-chart origin while preserving the relative ranking.

## Intra-day real-time scheduling based on model predictive control

While the day-ahead plan provides an economic baseline, forecasting errors of renewable generation and loads inevitably cause real-time power imbalances. To suppress these fluctuations, an intra-day scheduling strategy based on model predictive control is established, utilizing the latest measurements for multi-step rolling optimization to minimize tracking deviations and ensure physical safety.

### State-space model of the multi-energy microgrid

The foundation of the model predictive control framework is the state space representation of the energy storage assets within the microgrid. The state vector is defined to include the normalized states of the battery hydrogen system and thermal storage tank. The mathematical definition of the state vector is shown in Eq (51).


$$\begin{array}{c} x(k)=[S_{SOC}(k),S_{SOHC}(k),S_{HOC}(k){]}^{T} \end{array}$$ (51)

The control vector encompasses all dispatchable active powers and thermal powers within the system as shown in Eq (52).


$$\begin{array}{c} u(k)=[P_{bat}^{cha}(k),P_{bat}^{dis}(k),P_{EL}(k),P_{FC}(k),Q_{tst}^{cha}(k),Q_{tst}^{dis}(k),P_{EB}(k),P_{GB}(k){]}^{T} \end{array}$$ (52)

The uncontrollable disturbance vector consists of the renewable energy generation and the multi-energy loads as formulated in Eq (53).


$$\begin{array}{c} w(k)=[P_{W}(k),P_{PV}(k),P_{load}(k),Q_{load}(k){]}^{T} \end{array}$$ (53)

The discrete time state space dynamic update equation for the microgrid storage systems is formulated in Eq (54).


$$\begin{array}{c} x(k+1)=Ax(k)+Bu(k) \end{array}$$ (54)

where matrix A is the identity matrix representing the state retention capability as shown in Eq (55). Matrix B is the control input matrix defining the conversion efficiencies and capacities of the storage devices as detailed in Eq (56).


$$\begin{array}{c} A=[\begin{array}{ccc} 1 & 0 & 0\\ 0 & 1 & 0\\ 0 & 0 & 1 \end{array}] \end{array}$$ (55)

$$\begin{array}{c} B=\Delta t[\begin{array}{cccccccc} \frac{\eta_{bat}^{cha}}{E_{bat}^{cap}} & \frac{-1}{\eta_{bat}^{dis}E_{bat}^{cap}} & 0 & 0 & 0 & 0 & 0 & 0\\ 0 & 0 & \frac{\gamma_{EL}}{E_{HSS}^{cap}} & \frac{-\gamma_{FC}}{E_{HSS}^{cap}} & 0 & 0 & 0 & 0\\ 0 & 0 & 0 & 0 & \frac{\eta_{tst}^{cha}}{E_{tst}^{cap}} & \frac{-1}{\eta_{tst}^{dis}E_{tst}^{cap}} & 0 & 0 \end{array}] \end{array}$$ (56)

Over a finite prediction horizon length of $N_{p}$ the future state variables can be recursively predicted based on the current state and the future control sequence. The predicted state at step *k* + *i* is shown in Eq (57).


$$\begin{array}{c} x(k+i|k)=A^{i}x(k|k)+\sum_{j=0}^{i-1}A^{i-1-j}Bu(k+j|k) \end{array}$$ (57)

By aggregating the predictions across the entire horizon the vectorized state prediction model is constructed as shown in Eq (58).


$$\begin{array}{c} X(k)=\Phi x(k|k)+\Gamma U(k) \end{array}$$ (58)

where *X*(*k*) and *U*(*k*) represent the aggregated state sequence and respectively acontrol sequences defined in Eqs (59) and (60).


$$\begin{array}{c} X(k)=[x(k+1|k{)}^{T},x(k+2|k{)}^{T},...,x(k+N_{p}|k{)}^{T}{]}^{T} \end{array}$$ (59)

$$\begin{array}{c} U(k)=[u(k|k{)}^{T},u(k+1|k{)}^{T},...,u(k+N_{p}-1|k{)}^{T}{]}^{T} \end{array}$$ (60)

The coefficient matrices Φ and Γ are constant matrices derived from the system dynamics as formulated in Eqs (61) and (62).


$$\begin{array}{c} \Phi =[A^{T},(A^{2}{)}^{T},...,(A^{N_{p}}{)}^{T}{]}^{T} \end{array}$$ (61)

$$\begin{array}{c} \Gamma =[\begin{array}{cccc} B & 0 & ... & 0\\ AB & B & ... & 0\\ \vdots & \vdots & \ddots & \vdots \\ A^{N_{p}-1}B & A^{N_{p}-2}B & ... & B \end{array}] \end{array}$$ (62)

### Rolling-optimization objective function

The intra-day rolling controller uses one tracking formulation throughout the manuscript. Specifically, the optimized control sequence tracks the day-ahead control reference in Eq (19), while the predicted storage states are governed by Eqs (54)–(58) and are penalized only at the end of the prediction horizon. The unique rolling objective is


$$\begin{array}{cc} J^{⋆}(k)=\min_{U(k)}\{ & \sum_{i=1}^{N_{p}}\left[J_{track}(i)+J_{ope}(i)+J_{curtail}(i)\right]\\ & +J_{term}(k+N_{p})\}. \end{array}$$ (63)

The stage-wise tracking term measures the weighted quadratic deviation between the real-time control action and the corresponding day-ahead control reference:


$$\begin{array}{c} J_{track}(i)={\left\|u(k+i-1|k)-u^{ref}(k+i-1)\right\|}_{W}^{2}, \end{array}$$ (64)

where $\|z{\|}_{W}^{2}=z^{T}Wz$ and W≻0 is a diagonal weighting matrix. Before evaluating this quadratic term, each control-channel deviation is divided by the corresponding device upper power limit, so the entries of *W* are dimensionless and comparable across electric and thermal devices. The implemented value is W=diag(1.0,1.0,0.8,0.8,0.6,0.6,0.5,0.5) following the control-vector order in Eq (52); it is also reported in Table 4. Thus, the battery, hydrogen-system, thermal-storage, electric-boiler, and gas-boiler control powers track their day-ahead schedules using one consistent control-variable formulation.

To prevent the rolling horizon from producing an undesirable terminal drift in the stored-energy levels, the terminal storage-state penalty is defined as


$$\begin{array}{c} J_{term}(k+N_{p})={\left\|x(k+N_{p}|k)-x^{ref}(k+N_{p})\right\|}_{S}^{2}, \end{array}$$ (65)

where S⪰0 is the terminal-state weighting matrix and $x^{ref}(k+N_{p})$ contains the day-ahead terminal values of the battery state of charge, hydrogen state of charge, and thermal-storage state of charge. Because the three storage states are normalized quantities, the adopted terminal weighting matrix is S=diag(20,20,10), as listed in Table 4. This terminal term is not a second stage-wise tracking objective; it is used only to preserve end-of-horizon energy consistency.

For recursive-feasibility and stability certification, the terminal state is additionally constrained to a compact set centered on the day-ahead terminal reference:


$$\begin{array}{c} x(k+N_{p}|k)\in {𝒳}_{f}(k+N_{p}), \end{array}$$ (66)

$$\begin{array}{c} {𝒳}_{f}(k+N_{p})=\left\{x:\;x_{min}\leq x\leq x_{max},\;\left|x-x^{ref}(k+N_{p})\right|\leq \delta_{f}\right\}, \end{array}$$ (67)

where the inequalities are componentwise and $\delta_{f}=[0.05,0.05,0.08{]}^{T}$ for the battery, hydrogen, and thermal-storage states, respectively. The terminal set is verified offline to admit a feasible local backup policy that satisfies the state, input, ramping, power-balance, and mixed-integer mode constraints.

The real-time operation cost includes natural-gas consumption and equipment operating or depreciation costs:


$$\begin{array}{c} J_{ope}(i)=c^{T}u(k+i-1|k). \end{array}$$ (68)

Renewable-energy curtailment is penalized according to


$$\begin{array}{c} J_{curtail}(i)=\lambda_{W}\Delta P_{W}(k+i|k)+\lambda_{PV}\Delta P_{PV}(k+i|k), \end{array}$$ (69)

where $\lambda_{W}$ and $\lambda_{PV}$ are the wind- and photovoltaic-curtailment penalty coefficients, respectively, and $\Delta P_{W}$ and $\Delta P_{PV}$ denote the curtailed active powers. Both coefficients are set to $\lambda_{W}=\lambda_{PV}=2.00$ CNY/kWh in the case study; the complete MPC weighting settings are provided in Table 4. Accordingly, Eqs (63)–(69) constitute one control-sequence-based MPC objective, with a separate terminal-state regularization term.

### Operational constraints in the real-time stage

The real-time dispatch must strictly satisfy physical boundary conditions. The electrical and thermal power balance constraints must hold for every prediction step as formulated in Eqs (70) and (71).


$$\begin{array}{c} P_{grid}(k+i)+P_{W}^{actual}+P_{PV}^{actual}+P_{FC}+P_{bat}^{dis}=P_{load}+P_{EL}+P_{bat}^{cha}+P_{EB} \end{array}$$ (70)

$$\begin{array}{c} Q_{GB}+\eta_{EB}P_{EB}+Q_{tst}^{dis}+\eta_{EL}^{heat}P_{EL}+\eta_{FC}^{heat}P_{FC}=Q_{load}+Q_{tst}^{cha} \end{array}$$ (71)

The intra-day exchange variable $P_{grid}(k+i)$ in Eq (70) follows the same positive-import and negative-export convention as $P_{MG}(t)$ in the day-ahead model. The state variables are bounded by safety limits to prevent overcharging and deep discharging as shown in Eq (72).


$$\begin{array}{c} x_{min}\leq x(k+i|k)\leq x_{max} \end{array}$$ (72)

The physical output limits for all controllable devices are represented by Eq (73).


$$\begin{array}{c} u_{min}\leq u(k+i-1|k)\leq u_{max} \end{array}$$ (73)

To preserve equipment lifespan strict ramping constraints are applied to limit the power variation rate between adjacent control steps as shown in Eq (74).


$$\begin{array}{c} -\Delta R_{max}\leq u(k+i-1|k)-u(k+i-2|k)\leq \Delta R_{max} \end{array}$$ (74)

### Transformation into a mixed-integer quadratic program

The presence of complementary operational states such as charging and discharging creates nonconvex feasible regions. Binary logic variables are introduced to enforce mutual exclusivity. The battery operating constraints are shown in Eq (75).


$$\begin{array}{c} v_{bat}^{cha}(k+i)+v_{bat}^{dis}(k+i)\leq 1 \end{array}$$ (75)

where *v* represents binary decision variables. The nonlinear complementary constraints are converted into linear forms using the Big M relaxation method as formulated in Eqs (76) and (77).


$$\begin{array}{c} 0\leq P_{bat}^{cha}(k+i)\leq Mv_{bat}^{cha}(k+i) \end{array}$$ (76)

$$\begin{array}{c} 0\leq P_{bat}^{dis}(k+i)\leq Mv_{bat}^{dis}(k+i) \end{array}$$ (77)

where the Big-M coefficient is fixed at *M* = 500 kW in the binary-linked power constraints. The largest binary-linked equipment rating in Table 2 is the 300 kW gas-boiler fuel-input limit; therefore, *M* = 500 kW is 1.67 times this largest bound and satisfies $M\geq \max_{i}P_{i}^{\max}$ without using an excessively loose constant. The physical upper bounds $P_{i}^{\max}$ remain active in the device constraints, so *M* is used only to enforce the on/off logic rather than to replace the engineering ratings. Similar logic is applied to the hydrogen energy storage system to separate the operation of the electrolyzer and fuel cell as shown in Eqs (78)–(80).

**Table 2:** Key physical equipment and operational parameters of the microgrid.

Parameter Name	Set Value
**Renewable Generation**	
Wind rated capacity	300 kW
PV rated capacity	400 kW
**Battery Energy Storage System**	
Energy capacity $E_{bat}^{cap}$	600 kWh
Maximum charging/discharging power	180/180 kW
Charge efficiency	0.90
Discharge efficiency	0.90
Initial SOC	0.50
Safe range	[0.20, 0.80]
**Hydrogen Energy Storage System**	
Hydrogen-storage capacity $E_{HSS}^{cap}$	120 ${\text{kg-H}}_{2}$
Electrolyzer rated power	220 kW
Fuel-cell rated power	180 kW
Hydrogen lower heating value $L_{H_{2}}$	33.3 kWh/${\text{kg-H}}_{2}$
EL hydrogen-production efficiency $\eta_{EL}^{H_{2}}$	0.70
FC electrical efficiency $\eta_{FC}^{el}$	0.55
EL mass-conversion coefficient $\gamma_{EL}$	0.0210 ${\text{kg-H}}_{2}$/kWh
FC mass-consumption coefficient $\gamma_{FC}$	0.0546 ${\text{kg-H}}_{2}$/kWh
Initial SOHC	0.50
Safe range	[0.20, 0.80]
**Heat Conversion and Thermal Storage**	
Electric-boiler rated input power	180 kW
Gas-boiler rated fuel-input power	300 kW
GB thermal efficiency	0.73
EB thermal efficiency	0.90
Thermal-storage capacity $E_{tst}^{cap}$	800 ${\text{kWh}}_{th}$
Maximum thermal charging/discharging power	200/200 ${\text{kW}}_{th}$
Charge thermal efficiency	0.90
Discharge thermal efficiency	0.90
Initial HOC	0.50
Safe range	[0.20, 0.80]
**Boundary and Dynamic Limits**	
Tie-line import/export limits $P_{MG}^{imp,max}/P_{MG}^{exp,max}$	350/350 kW
Control ramp-limit vector $\Delta R_{max}$	$[60,60,55,45,70,70,60,80{]}^{T}$


$$\begin{array}{c} v_{HSS}^{EL}(k+i)+v_{HSS}^{FC}(k+i)\leq 1 \end{array}$$ (78)

$$\begin{array}{c} 0\leq P_{EL}(k+i)\leq Mv_{HSS}^{EL}(k+i) \end{array}$$ (79)

$$\begin{array}{c} 0\leq P_{FC}(k+i)\leq Mv_{HSS}^{FC}(k+i) \end{array}$$ (80)

By expanding the quadratic tracking error terms and integrating the linear constraints the entire predictive control model is transformed into a standard Mixed Integer Quadratic Programming problem. The canonical matrix representation is shown in Eq (81).


$$\begin{array}{c} \min\frac{1}{2}U(k{)}^{T}ℋU(k)+{ℱ}^{T}U(k) \end{array}$$ (81)

where ℋ is the global positive definite Hessian matrix containing all quadratic weight coefficients and ℱ is the global linear coefficient vector incorporating reference trajectories and operation costs. All inequality and equality constraints derived from physical limits and energy balances are mapped into global affine matrices as formulated in Eqs (82) and (83).


$$\begin{array}{c} {𝒜}_{in}U(k)\leq {ℬ}_{in} \end{array}$$ (82)

$$\begin{array}{c} {𝒜}_{eq}U(k)={ℬ}_{eq} \end{array}$$ (83)

Solving this standardized problem yields the optimal control sequence at step *k*. Following the receding horizon principle, only the first element *u*(*k*|*k*) is executed before the optimization window advances.

### Recursive feasibility and closed-loop stability

The stability analysis is first established for the nominal deviation dynamics $\tilde{x}=x-x^{ref}$ and $\tilde{u}=u-u^{ref}$. Because the objective contains both tracking and economic terms, define the rotated stage cost


$$\begin{array}{c} \ell_{r}(x,u)=\ell (x,u)-\ell (x^{ref},u^{ref})+\Lambda (x)-\Lambda \;\left(Ax+Bu\right), \end{array}$$ (84)

where $\ell =J_{track}+J_{ope}+J_{curtail}$ and Λ(x) is a storage function used in the economic-MPC dissipativity construction.

**Assumption 2** *The day-ahead reference pair* $\left(x^{ref},u^{ref}\right)$ *satisfies the nominal dynamics and all hard constraints. There exists a terminal backup controller* $\kappa_{f}(x)$ *and the terminal set* $X_{f}$ *in* Eq (67) *such that, for every* $x\in X_{f}$*: (i)* $\kappa_{f}(x)$ *and its terminal mixed-integer mode satisfy all input, ramping, and balance constraints; (ii)* $Ax+B\kappa_{f}(x)\in X_{f}$*; and (iii) the terminal cost* $V_{f}(x)=\|x-x^{ref}{\|}_{S}^{2}$ *satisfies*


$$\begin{array}{c} V_{f}\;\left(Ax+B\kappa_{f}(x)\right)-V_{f}(x)\leq -\ell_{r}\;\left(x,\kappa_{f}(x)\right), \end{array}$$ (85)

*where $\ell_{r}(x,u)\geq \alpha_{\ell }(\|x-x^{ref}\|)$ for a class-$K_{\infty }$ function $\alpha_{\ell }$.*

To make the terminal-set condition reproducible, $X_{f}$ is verified offline using a finite test set that contains the vertices of the box in Eq (67) together with uniformly distributed interior samples. For every test state $x^{\left(q\right)}\in X_{f}$, a one-step mixed-integer feasibility problem is solved to obtain a backup action $\kappa_{f}(x^{\left(q\right)})$ satisfying the input, ramping, energy-balance, and operating-mode constraints, while enforcing $Ax^{\left(q\right)}+B\kappa_{f}(x^{\left(q\right)})\in X_{f}$. For each feasible backup action, the terminal-decrease residual


$$\begin{array}{c} d_{q}=V_{f}\;\left(Ax^{\left(q\right)}+B\kappa_{f}(x^{\left(q\right)})\right)-V_{f}\;\left(x^{\left(q\right)}\right)+\ell_{r}\;\left(x^{\left(q\right)},\kappa_{f}(x^{\left(q\right)})\right) \end{array}$$ (86)

is evaluated. The numerical verification reports the feasible-sample ratio and $\max_{q}d_{q}$; the terminal condition is accepted for the reported case only when every tested state admits a feasible backup action and the maximum decrease residual is non-positive. The corresponding numerical evidence is reported in the simulation analysis.

**Theorem 2 (Nominal recursive feasibility).** *If the MPC problem defined by* Eqs (63)–(83) *and the terminal constraint in* Eq (66) *is feasible at time k, then it remains feasible at time k + 1 for the nominal prediction model.*

*Proof.* Let $U^{⋆}(k)=\{u^{⋆}(k|k),...,u^{⋆}(k+N_{p}-1|k)\}$ be a feasible optimal sequence. After applying $u^{⋆}(k|k)$ to the nominal model, construct the candidate sequence at the next sampling instant by shifting the unused controls and appending the terminal backup action:


$$\begin{array}{c} \hat{U}(k+1)=\{u^{⋆}(k+1|k),...,u^{⋆}(k+N_{p}-1|k),\kappa_{f}(x(k+N_{p}|k))\}. \end{array}$$ (87)

Under the nominal dynamics, the shifted predicted states coincide with the tail of the previously feasible trajectory. Hence, the intermediate state, input, ramping, balance, and mixed-integer constraints remain satisfied. Assumption 2(i)–(ii) guarantees feasibility of the appended terminal input and invariance of $X_{f}$. Therefore, $\hat{U}(k+1)$ is feasible. □

**Theorem 3** (Nominal closed-loop stability). *Under Assumption 2, the optimal finite-horizon value function satisfies*


$$\begin{array}{c} V_{N_{p}}^{⋆}(x(k+1))-V_{N_{p}}^{⋆}(x(k))\leq -\alpha_{\ell }(\|x(k)-x^{ref}(k)\|). \end{array}$$ (88)

*Consequently, the nominal tracking error is asymptotically stable on the recursively feasible set.*

*Proof.* Evaluate the objective at time *k* + 1 using the shifted feasible sequence in Eq (87). The common stage terms cancel when it is compared with the optimal objective at time *k*. Applying the terminal decrease condition in Eq (85) yields Eq (88), so $V_{N_{p}}^{⋆}$ is a Lyapunov function for the nominal closed loop [35]. □

For a bounded forecast and state-estimation mismatch *e*(*k*), a practical-stability statement can be made only when the realized successor state remains inside the feasible region, for example through verified operating margins or a constraint-tightened/tube-based implementation. If, on that feasible region, the value function satisfies the perturbed decrease inequality


$$\begin{array}{c} V_{N_{p}}^{⋆}(x(k+1))-V_{N_{p}}^{⋆}(x(k))\leq -\alpha_{\ell }(\|x(k)-x^{ref}(k)\|)+\sigma_{e}(\|e(k)\|), \end{array}$$ (89)

then the standard perturbed-Lyapunov argument gives the conditional bound


$$\begin{array}{c} {lim sup}_{k\to \infty }\|x(k)-x^{ref}(k)\|\leq \alpha_{\ell }^{-1}\;\left(\sigma_{e}(\bar{e})\right),\;\bar{e}=\sup_{k}\|e(k)\|. \end{array}$$ (90)

**Remark 2** *Theorems 2 and 3 provide nominal guarantees.* Equations (89)–(90) *characterize bounded-error behavior conditionally and do not constitute a robust recursive-feasibility proof for the nominal mixed-integer MPC. A formal robust guarantee under arbitrary* e(k)∈E *would require an explicit constraint-tightening, tube-MPC, or robust-invariant-set construction. The present simulations therefore evaluate the empirical feasibility and economic sensitivity under forecast errors, while this robust extension is reserved for future work* [36*].*

Algorithm 1 summarizes the scheduling strategy.

**Algorithm 1 Privacy-Preserving Multi-Time-Scale Scheduling Strategy**

**Require:** Forecast data, load data, equipment parameters, carbon trading parameters, ADMM parameters ρ, $\epsilon_{0}$, Δf, *c*, α, maximum iteration number $K_{\max}$, MPC horizon $N_{p}$, weighting matrices *W* and *S*, terminal set $X_{f}$, curtailment penalties $\lambda_{W}$ and $\lambda_{PV}$, and Big-M coefficient *M*.

**Ensure:** Day-ahead reference schedule, intra-day control trajectory, and cumulative privacy budget.

1: Initialize $P_{Grid}^{0}(t)$, $\lambda^{0}(t)$, *k* = 0, and $\epsilon_{cum}(0)=0$.

**Day-ahead privacy-preserving ADMM clearing**

2: **repeat**

3:   Solve the microgrid local optimization problem and obtain $P_{MG}^{k+1}(t)$.

4:   Set $b_{k+1}=c\alpha^{k+1}\Delta f/\epsilon_{0}$ and generate $\theta^{k+1}(t)\sim \mathrm{Lap}(0,b_{k+1})$.

5:   Transmit ${\tilde{P}}_{MG}^{k+1}(t)=P_{MG}^{k+1}(t)+\theta^{k+1}(t)$.

6:   The upper-level grid updates $P_{Grid}^{k+1}(t)$ according to ${\tilde{P}}_{MG}^{k+1}(t)$.

7:   Update $\lambda^{k+1}(t)=\lambda^{k}(t)+\rho \left({\tilde{P}}_{MG}^{k+1}(t)-P_{Grid}^{k+1}(t)\right)$.

8:   Compute $\epsilon_{k+1}=\Delta f/b_{k+1}$ and $\epsilon_{cum}(k+1)=\epsilon_{cum}(k)+\epsilon_{k+1}$.

9:   Compute the primal residual $r^{k+1}$ and dual residual $s^{k+1}$.

10:   *k*=*k*+1.

11: **until** $\left(r^{k}\leq \varepsilon_{pri}\text{ and }s^{k}\leq \varepsilon_{dual}\right)$ or $k=K_{\max}$

12: Set $K_{term}=k$, obtain the day-ahead reference trajectory $u^{ref}$, and report $r^{K_{term}}$, $s^{K_{term}}$, and $\epsilon_{cum}(K_{term})$.

**Intra-day MPC rolling correction**

13: **for** each intra-day sampling instant τ **do**

14:   Acquire real-time renewable generation, load, and storage state information.

15:   Build the prediction model X(τ)=Φx(τ|τ)+ΓU(τ).

16:   Solve the MIQP rolling optimization problem with tracking, operation cost, curtailment penalty, physical constraints, and the terminal-set constraint $x(\tau +N_{p}|\tau )\in X_{f}$.

17:   Apply the first optimal control action $u^{*}(\tau |\tau )$

18:   Move the prediction horizon forward

19: **end for**

20: Output the day-ahead clearing result and intra-day scheduling trajectory.

## Simulation results and analysis

### Case-study parameters and scenario settings

To evaluate the proposed multi-time-scale electricity–carbon joint clearing model and distributed privacy-preserving optimization strategy, a representative grid-connected hydrogen microgrid is used in the case study. The simulation is implemented in MATLAB R2021b with YALMIP and CPLEX for the resulting mixed-integer quadratic programs. At each ADMM iteration, the local mixed-integer subproblem is solved before the boundary-power and multiplier updates are performed. The CPLEX relative MIP gap is set to $1.0\times 10^{-6}$, and the remaining algorithmic settings are summarized in Table 4.

Regarding the configuration of the scheduling framework, the day-ahead global scheduling horizon is 24 h with a 1 h time step. The intra-day simulation also covers 24 h, corresponding to 96 control intervals with a sampling period of 15 min. At each sampling instant, the MPC optimizer uses a prediction horizon of $N_{p}=8$ intervals, i.e., a 2 h look-ahead window, applies only the first optimized control action, and then shifts the horizon forward by one 15 min interval. This distinction between the 24 h simulation duration and the 2 h receding prediction horizon is now stated explicitly. The primary boundary input conditions of the microgrid system include the forecasted output values of wind power and photovoltaic power, along with the foundational electrical and thermal load demands within the system. In this case study scenario, photovoltaic output is primarily concentrated during the daytime from 08:00–16:00, while wind power exhibits distinct anti-peak regulation characteristics with high output during nocturnal periods and flat output during the day. Furthermore, thermal load demands peak at night due to rigid heating requirements, presenting a significant spatial and temporal misalignment with the peak photovoltaic output. This physical characteristic of sources and loads objectively requires the system to be equipped with comprehensive multi-medium energy storage support units, including battery, hydrogen, and thermal energy storage, to achieve cross-period energy transfer and flexible supply–demand balance. The tie line is modeled as bidirectional throughout the case study. Thus, the optimizer may select $P_{MG}(t)>0$ to cover a local power deficit or $P_{MG}(t)<0$ to export a residual surplus after local loads and storage requirements have been satisfied. The operational parameters, energy conversion efficiencies, and initial capacities of key physical equipment within the system are established based on practical engineering ranges and the scale of the source–load profiles used in the case study. The adopted ratings are 300 kW for wind generation, 400 kW for photovoltaic generation, 600 kWh/180 kW for the battery, 120 ${\text{kg-H}}_{2}$ for hydrogen storage, 220 kW for the electrolyzer, 180 kW for the fuel cell, 180 kW for the electric boiler, 300 ${\text{kW}}_{fuel}$ for the gas boiler, and 800 ${\text{kWh}}_{th}/200\;{\text{kW}}_{th}$ for thermal storage. The bidirectional tie-line limit is 350 kW. The specific boundary constraint settings for equipment parameters are presented in Table 2.

The ramp-limit vector follows the control order in Eq (52); its entries are specified per 15-min MPC interval, with ${\text{kW}}_{th}$ used for the two thermal-storage channels. The baseline carbon-quota coefficient is $\mu_{quota}=0.150$ kg ${\text{CO}}_{2}/{\text{kWh}}_{eq}$.

Under the market environment of electricity–carbon joint clearing, the operational costs of the system encompass not only traditional fuel procurement and equipment depreciation but also deeply integrate carbon emission trading costs. This case study comprehensively considers the operation and maintenance characteristics of various microgrid units alongside external market prices. The essential economic evaluation parameters and carbon market conversion coefficients are detailed in Table 3. The direct natural-gas emission factor is set to $\mu_{GB}=0.202$ kg ${\text{CO}}_{2}/{\text{kWh}}_{fuel}$. This value is obtained by converting the IPCC default factor of 56.1 kg CO_2_/GJ for natural gas in stationary combustion to a per-kWh fuel-energy basis [31]; it is applied to the fuel-input power $P_{GB}(t)$ rather than to the useful heat output. The base-case emission intensity of imported grid electricity is set to $\mu_{Grid}=0.570$ kg CO_2_/kWh. This factor is applied only to $P_{MG}^{imp}(t)$ in Eq (12), so the carbon cost reflects the indirect emissions of purchased electricity without assigning a negative emission credit to exported power. For reproducibility of the privacy experiment, adjacent datasets are defined as differing in one hourly boundary-power element after clipping, with the maximum *L*_1_ change limited to 5.00 kW. Consequently, the global sensitivity in Eq (27) is set to Δf=5.00 kW. In Eq (26), the privacy budget of the message released at iteration *k* is instantiated as $\epsilon_{k}$, while $\epsilon_{0}=0.50$ is the baseline calibration coefficient used in Eq (38). The numerical values of $\epsilon_{0}$, *c*, α, Δf, and the finite iteration horizon are included in Table 3.

**Table 3:** Market trading and economic evaluation parameters and privacy-preserving ADMM settings.

Parameter Name	Set Value
**Grid-Interaction Convention**	
Tie-line exchange mode	Bidirectional; export allowed
Boundary-power sign	$P_{MG}>0$: import; $P_{MG}<0$: export
Base-case settlement rule	Common time-of-use price for both directions
**Carbon Market and Emissions**	
Carbon trading price	0.224 CNY/kg
Direct natural-gas emission factor $\mu_{GB}$	0.202 kg ${\text{CO}}_{2}/{\text{kWh}}_{fuel}$
Grid-import emission intensity $\mu_{Grid}$	0.570 kg CO_2_/kWh
Baseline quota coefficient $\mu_{quota}$	0.150 kg ${\text{CO}}_{2}/{\text{kWh}}_{eq}$
**Fuel and Maintenance Costs**	
Natural gas price	0.331 CNY/kWh
Wind O&M cost	0.11 CNY/kWh
PV O&M cost	0.08 CNY/kWh
Battery O&M cost	0.20 CNY/kWh
Hydrogen O&M cost	0.20 CNY/kWh
Boiler and TES O&M cost	0.05 CNY/kWh
**Privacy-Preserving ADMM Parameters**	
Baseline privacy coefficient $\epsilon_{0}$	0.50
Noise initialization coefficient *c*	1.00
Noise decay factor α	0.95
Adjacency clipping bound	5.00 kW
Global sensitivity Δf	5.00 kW
Maximum released iterations $K_{\max}$	50

For reproducibility, the algorithmic parameters that are not physical equipment or market data are collected separately in Table 4. The same ADMM penalty coefficient and stopping tolerances are used for the noise-free, fixed-noise, and dynamically decaying-noise cases. The MPC weights are applied after normalizing each control deviation by the corresponding device upper power limit, while the storage states are already expressed in normalized form. The Big-M value is selected from the largest relevant device power bound rather than as an arbitrarily oversized number.

**Table 4:** Algorithmic and intra-day MPC parameter settings.

Parameter	Value used in the simulations
ADMM penalty coefficient ρ	1.00
Primal residual tolerance $\varepsilon_{pri}$	$1.0\times 10^{-3}$
Dual residual tolerance $\varepsilon_{dual}$	$1.0\times 10^{-3}$
Maximum ADMM iterations $K_{\max}$	50
Intra-day sampling interval $\Delta t_{MPC}$	15 min
MPC prediction horizon $N_{p}$	8 intervals (2 h)
Control-tracking weight *W*	diag(1.0,1.0,0.8,0.8,0.6,0.6,0.5,0.5)
Terminal-state weight *S*	diag(20,20,10)
Terminal-set radius $\delta_{f}$	$[0.05,0.05,0.08{]}^{T}$
Wind-curtailment weight $\lambda_{W}$	2.00 CNY/kWh
PV-curtailment weight $\lambda_{PV}$	2.00 CNY/kWh
Big-M coefficient *M*	500 kW
CPLEX relative MIP gap	$1.0\times 10^{-6}$

### Numerical convergence of the privacy-preserving ADMM scheme

**Fig 3:**
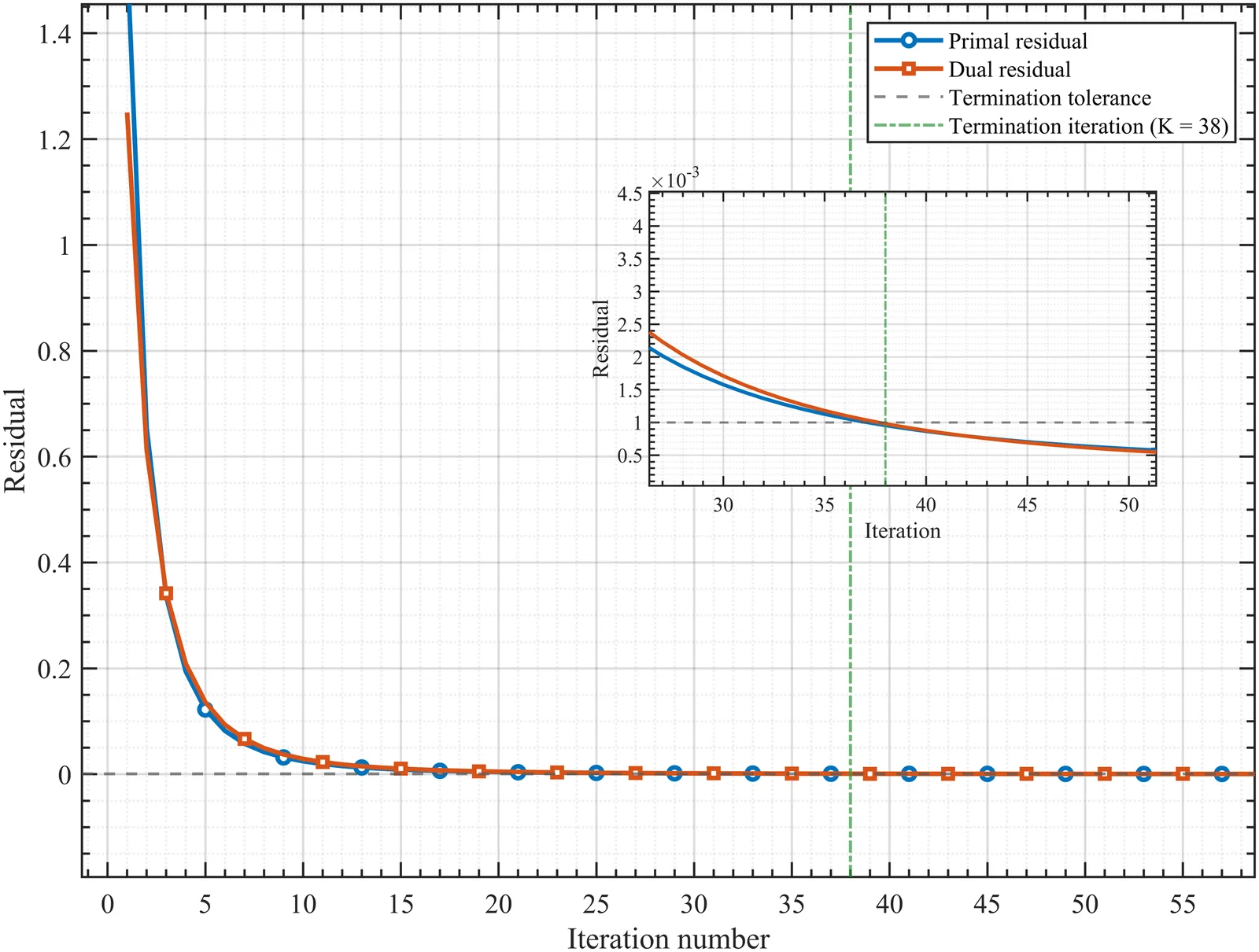
Convergence trajectories of the primal and dual residuals for the privacy-preserving ADMM scheme.

The convergence behavior of the day-ahead distributed clearing process is evaluated using the primal and dual residuals defined in Eqs (42) and (43). The stopping tolerances are set to $\varepsilon_{pri}=\varepsilon_{dual}=10^{-3}$. As shown in Fig 3, both residuals decrease rapidly during the initial iterations and then enter a gradual asymptotic stage. The magnified region further shows the approach of the two residuals to the prescribed tolerance.

The primal and dual residuals simultaneously satisfy the stopping criteria for the first time at iteration $K_{term}=\min\{k:r^{k}\leq \varepsilon_{pri},\;s^{k}\leq \varepsilon_{dual}\}=38$. At termination, the residual values are $r^{38}=9.54\times 10^{-4}$ and $s^{38}=9.79\times 10^{-4}$. The maximum-iteration safeguard is set to $K_{\max}=50$, so the observed stopping iteration satisfies $K_{term}=38<K_{\max}$. The reported run therefore terminates because both residual tolerances are met, not because the maximum-iteration limit is reached. These results provide direct numerical evidence that the privacy-preserving ADMM iterations reach the prescribed practical convergence conditions for the investigated mixed-integer case. The trajectories after the vertical termination marker are retained only to illustrate the asymptotic behavior of the residuals. They are obtained through offline continuation and are not included in the actual communication transcript or in the cumulative privacy-budget calculation.

Based on the aforementioned case study parameters and scenario settings, the subsequent sections present the numerical convergence of the distributed iterations, the multi-energy coordinated operational characteristics at the day-ahead time scale, the rolling tracking strategy at the intra-day time scale, a robustness analysis under multiple forecast-error levels, and sensitivity analyses under various parameters.

### Multi-energy coordinated operation at the day-ahead time scale

**Fig 4:**
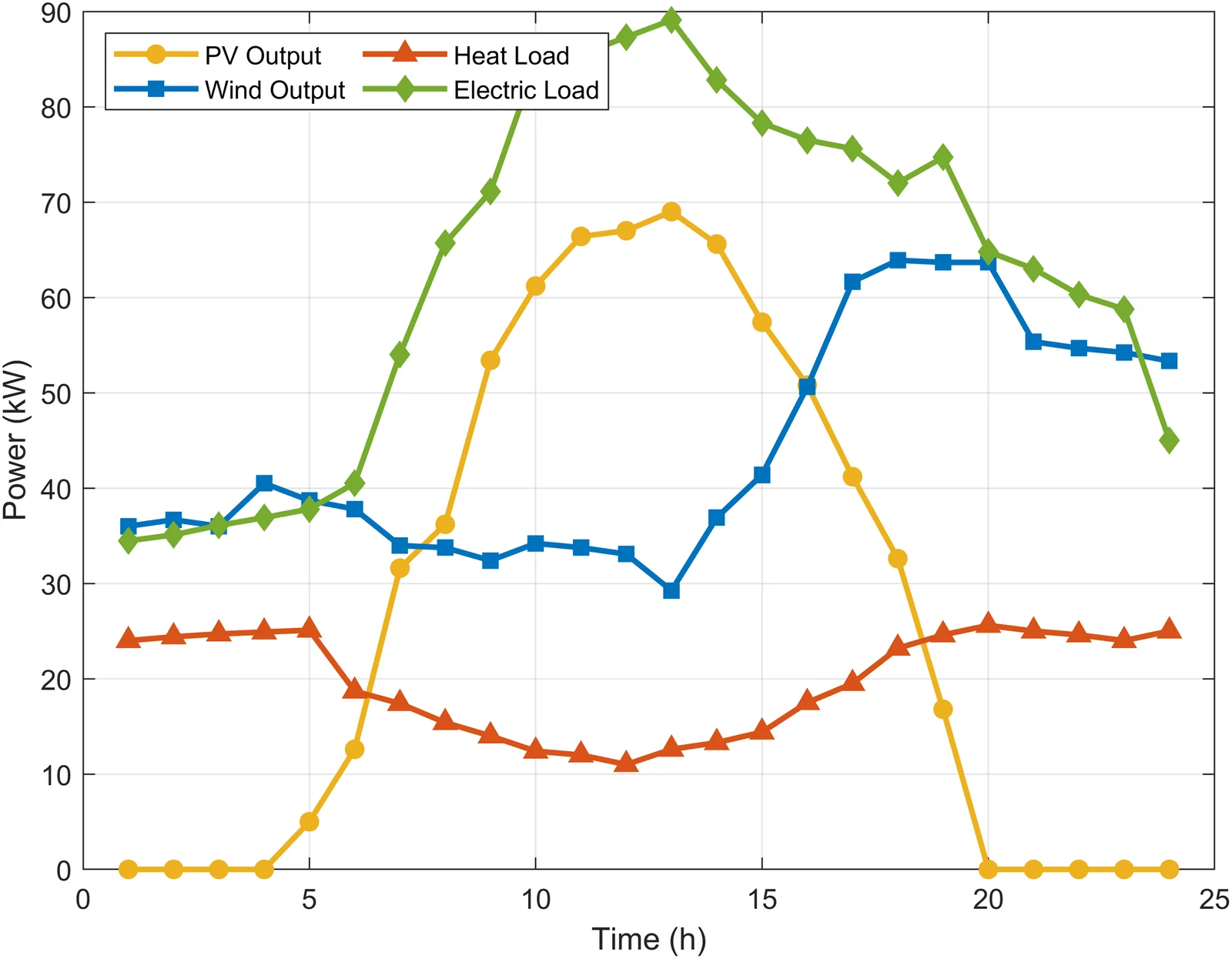
Source and load power prediction curves.

Under the day-ahead global optimal scheduling framework, the microgrid system exhibits an operational trend of high multi-energy flow coupling and cross-period complementarity. The system initially coordinates and allocates various energy conversion and storage devices based on precise perception of external renewable energy output and internal load demand. Observing the source and load power prediction curves shown in Fig 4, photovoltaic power output is concentrated during the daytime and reaches its peak at noon, while wind power output remains high during nocturnal and early morning periods, forming a preliminary complementarity in the temporal dimension. Simultaneously, the electrical load of the system is in a state of high demand during the day, whereas the thermal load peaks at night due to increased nocturnal heating demand.

**Fig 5:**
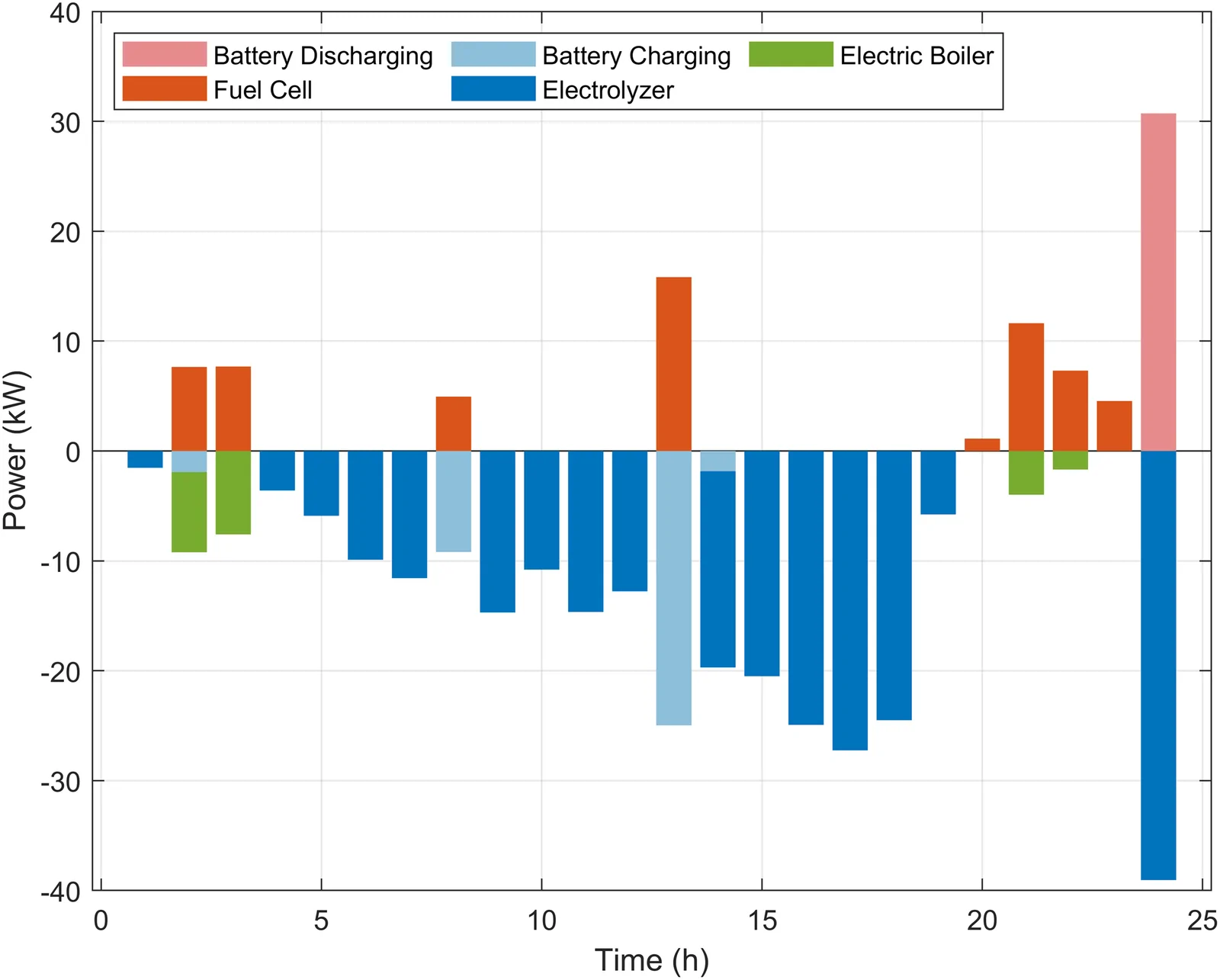
Day ahead electrical power balance of the microgrid.

**Fig 6:**
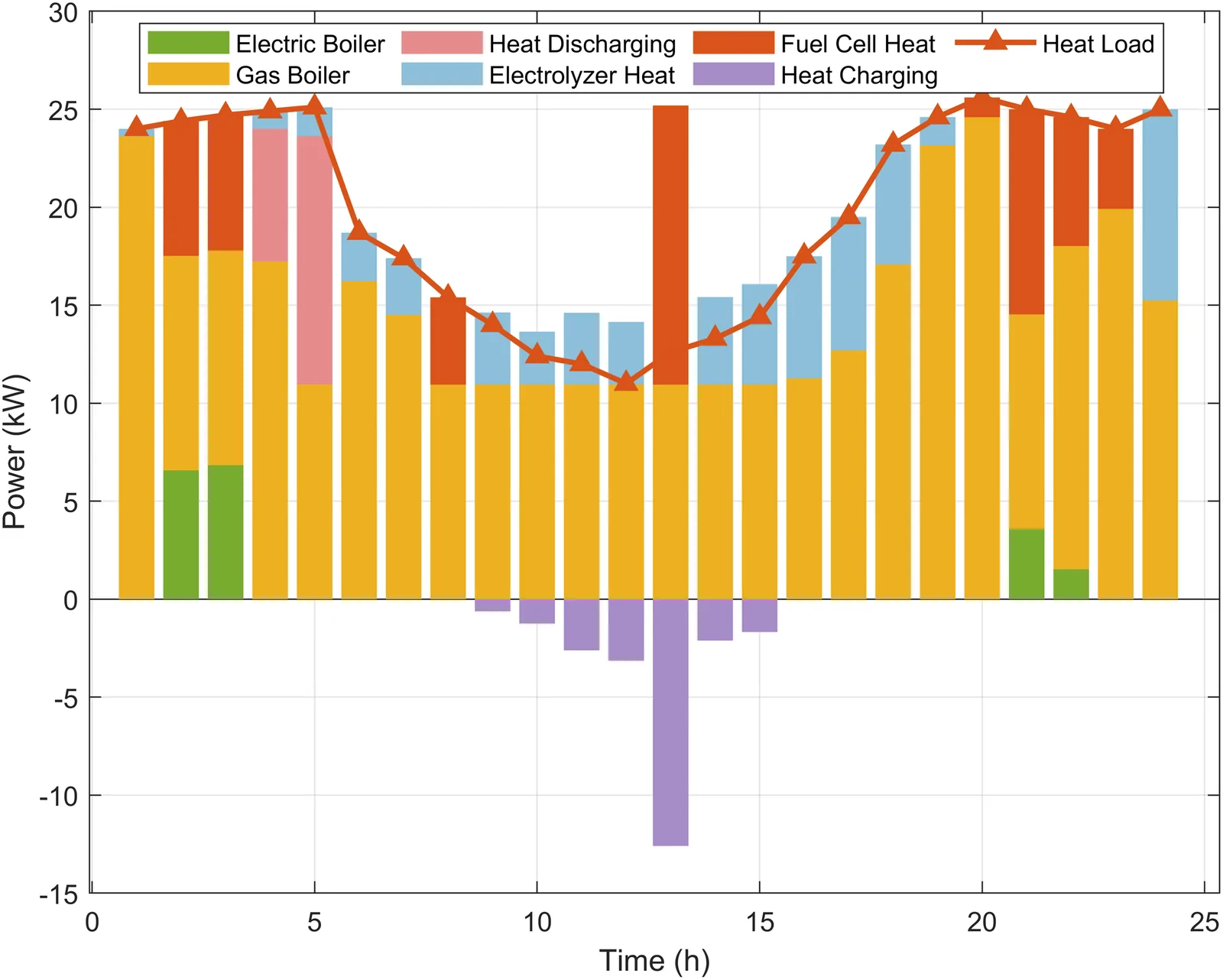
Day ahead thermal power balance of the microgrid.

To address the aforementioned spatial and temporal misalignment of sources and loads, the system achieves precise energy scheduling through the joint constraints of electrical power balance and thermal power balance as shown in Figs 5 and 6. Regarding electrical power balance, when the daytime photovoltaic output significantly exceeds the basic electrical load demand, the system does not generate photovoltaic curtailment. Instead, it injects all surplus electrical energy into the electrolyzer and the battery energy storage system. When photovoltaic output is absent at night and wind power is insufficient to support the electrical load, the fuel cell and battery energy storage system switch to the discharging state to compensate for the power deficit. Under the adopted carbon-accounting model, electricity imported from the upper-level grid incurs both the transaction cost in Eq (15) and the indirect carbon cost in Eq (12). Consequently, the scheduling model avoids treating grid imports as carbon free and gives greater economic preference to local renewable generation and stored energy when their operating constraints permit. Regarding thermal power balance, the high thermal load periods at night primarily rely on the gas boiler to provide a stable base heat source. During periods of abundant renewable energy in the daytime, the system activates the electric boiler for electricity to heat conversion and fully recovers the low grade waste heat generated during the operation of the electrolyzer, effectively reducing natural gas consumption and the corresponding carbon emissions.

**Fig 7:**
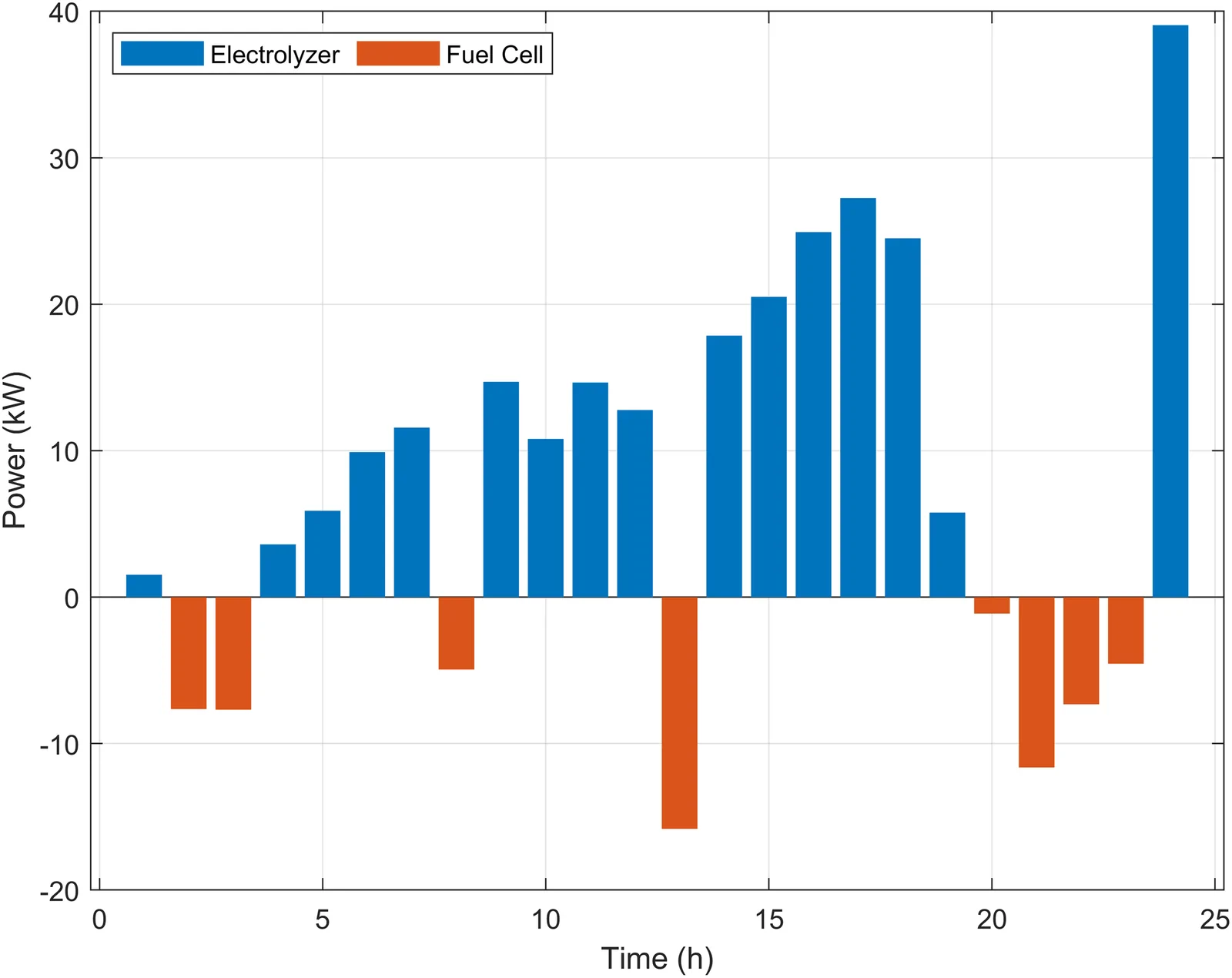
Coordinated operational status of electrolyzer and fuel cell.

The coordinated operating curves of the electrolyzer and fuel cell are further presented in Fig 7.

As the key component of the microgrid electricity–carbon joint scheduling, the hydrogen energy system clearly reflects the cross medium energy conversion process through the coordinated output curves of its internal electrolyzer and fuel cell as shown in Fig 7. During the renewable energy abundant period from 08:00–16:00, the electrolyzer, acting as a controllable flexible load, absorbs electrical energy on a large scale and converts it into hydrogen for storage. During the peak electrical energy demand periods from 01:00–07:00 and from 19:00–24:00, the fuel cell consumes hydrogen to generate power for the grid. In this process, the hydrogen energy system achieves bidirectional conversion between electrical energy and hydrogen energy, greatly enhancing the downward regulation capability of the microgrid.

**Fig 8:**
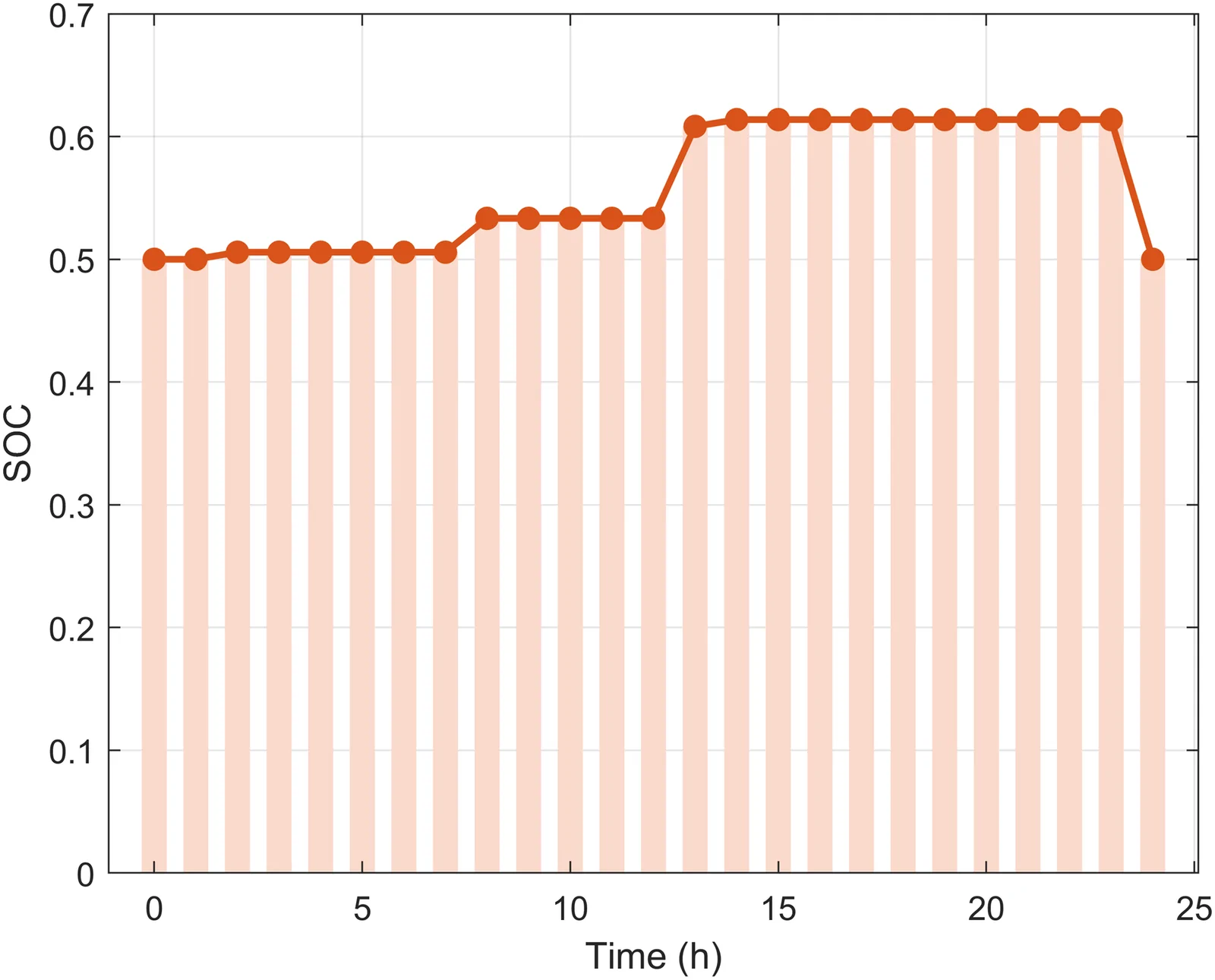
SOC profile of the battery energy storage system.

**Fig 9:**
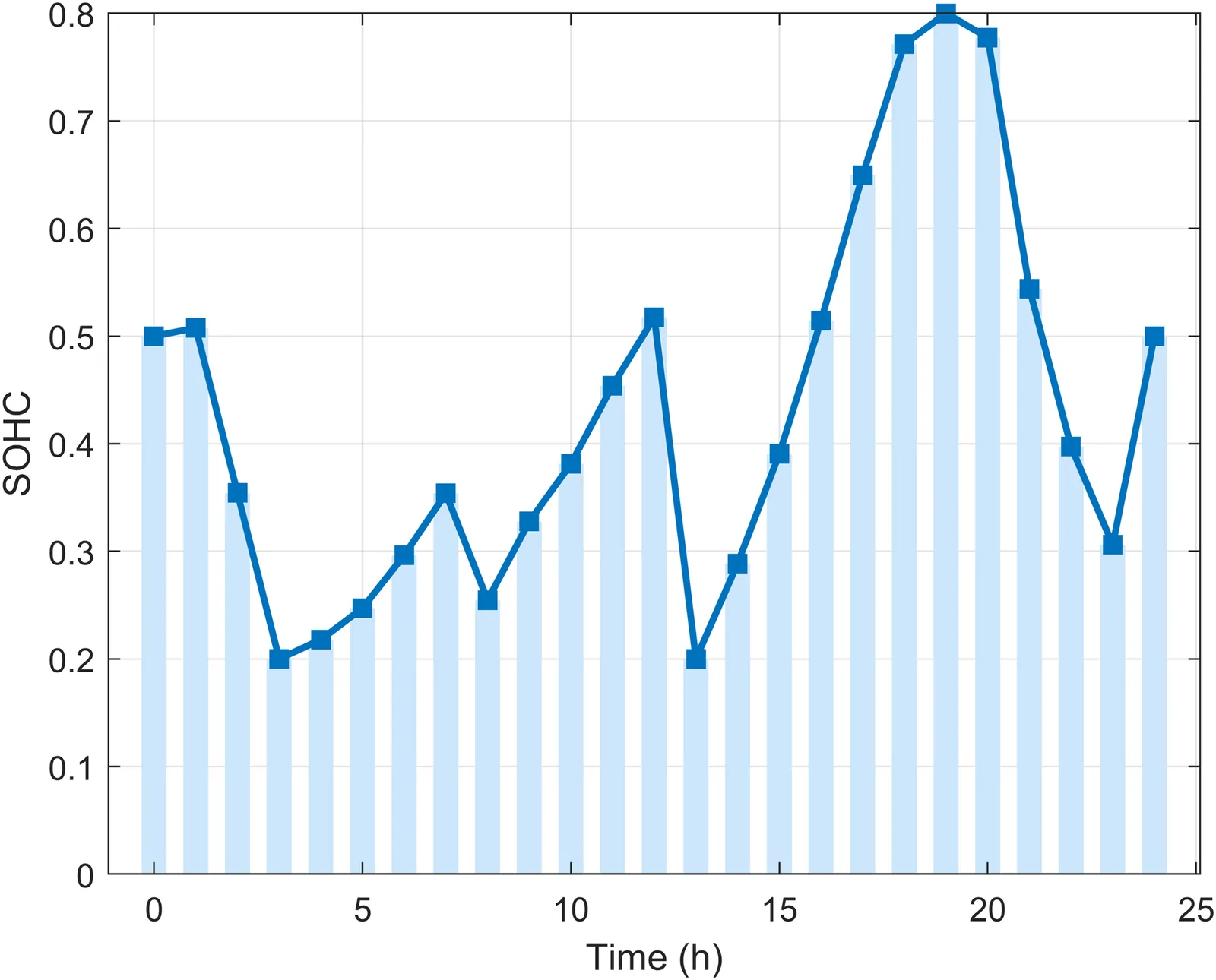
SOHC profile of the hydrogen energy storage system.

**Fig 10:**
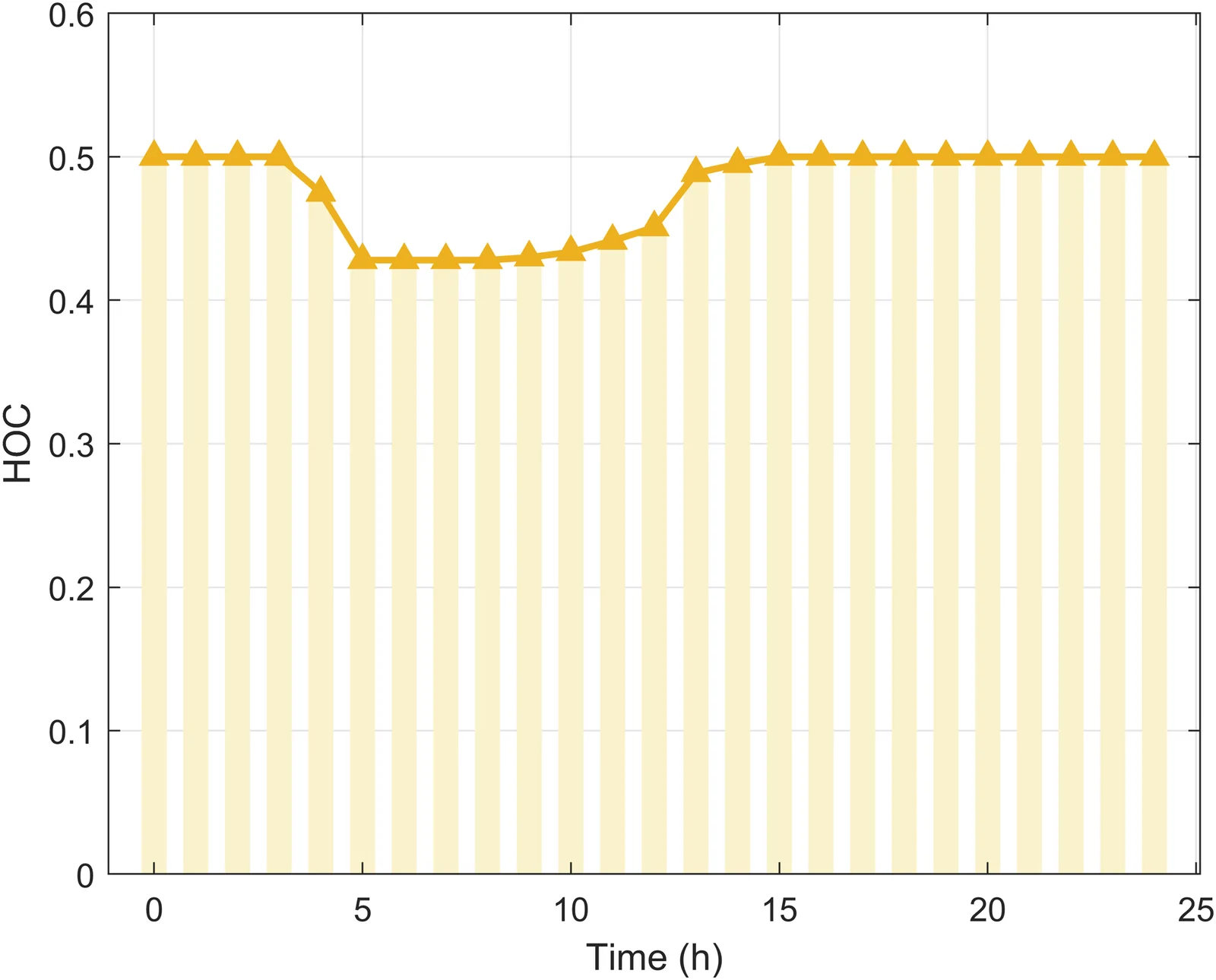
HOC profile of the thermal energy storage system.

**Fig 11:**
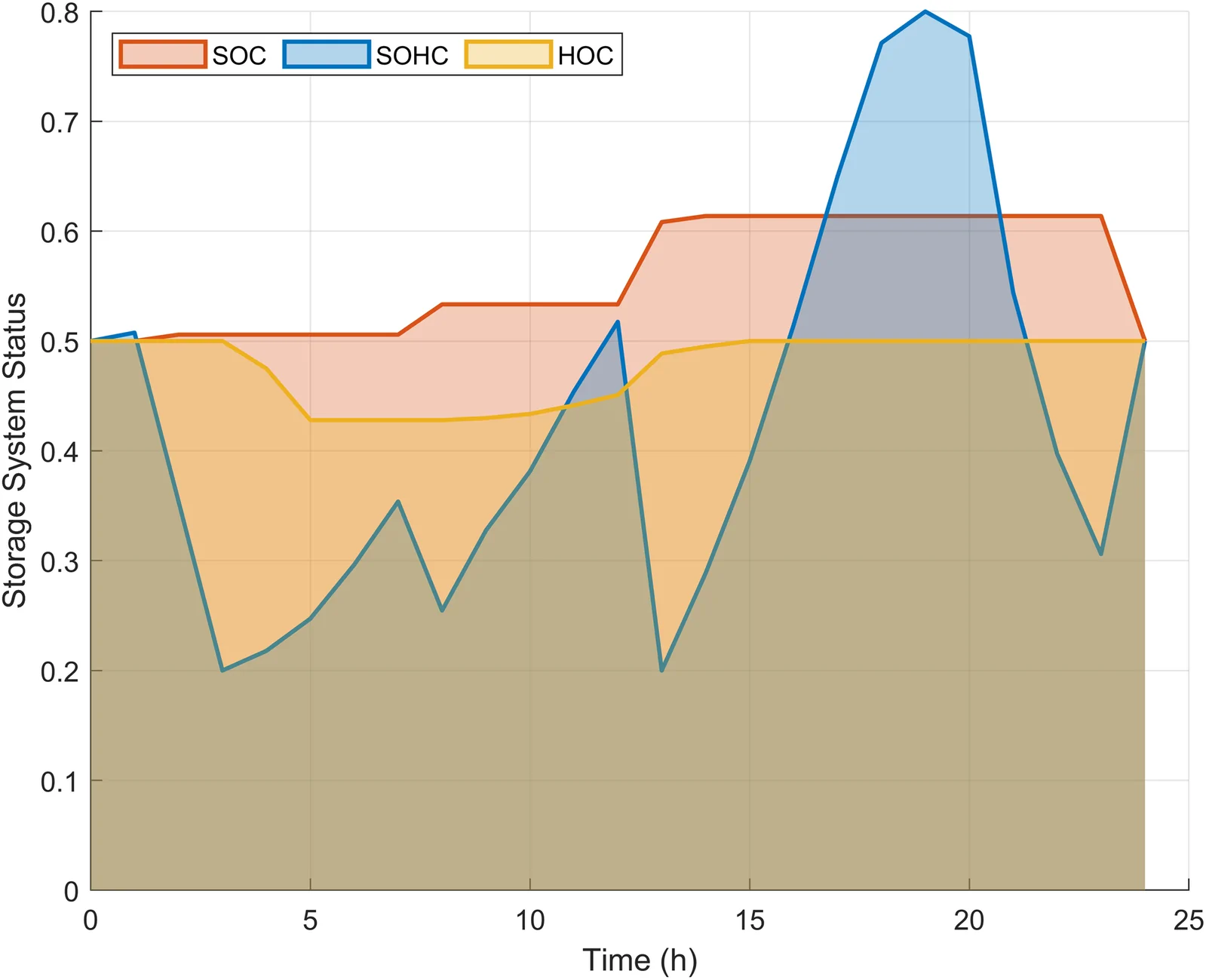
Coordinated operational status of the multi-dimensional energy storage system.

The storage-state trajectories used to evaluate the multi-medium coordination are presented in Figs 8–11.

The state changes of the multidimensional energy storage system further verify the flexible support capability of the system as shown in Figs 8–11. Among them, the state of charge of the battery energy storage primarily fluctuates gently within a narrow range, undertaking the task of smoothing short-term power fluctuations. The state of heat charge is closely bound to the heating demand of the system, functioning to shave peaks and fill valleys. The state of hydrogen charge exhibits wide range and long cycle charge and discharge characteristics, constituting the primary medium for cross-period energy transfer in the system. The translucent overlaid area chart of the three as shown in Fig 11 intuitively demonstrates the staggered peak complementary mechanism of multiple energy storage media in the time series, avoiding excessive charging and discharging of a single energy storage device, thereby ensuring the safety margin of the system.

**Fig 12:**
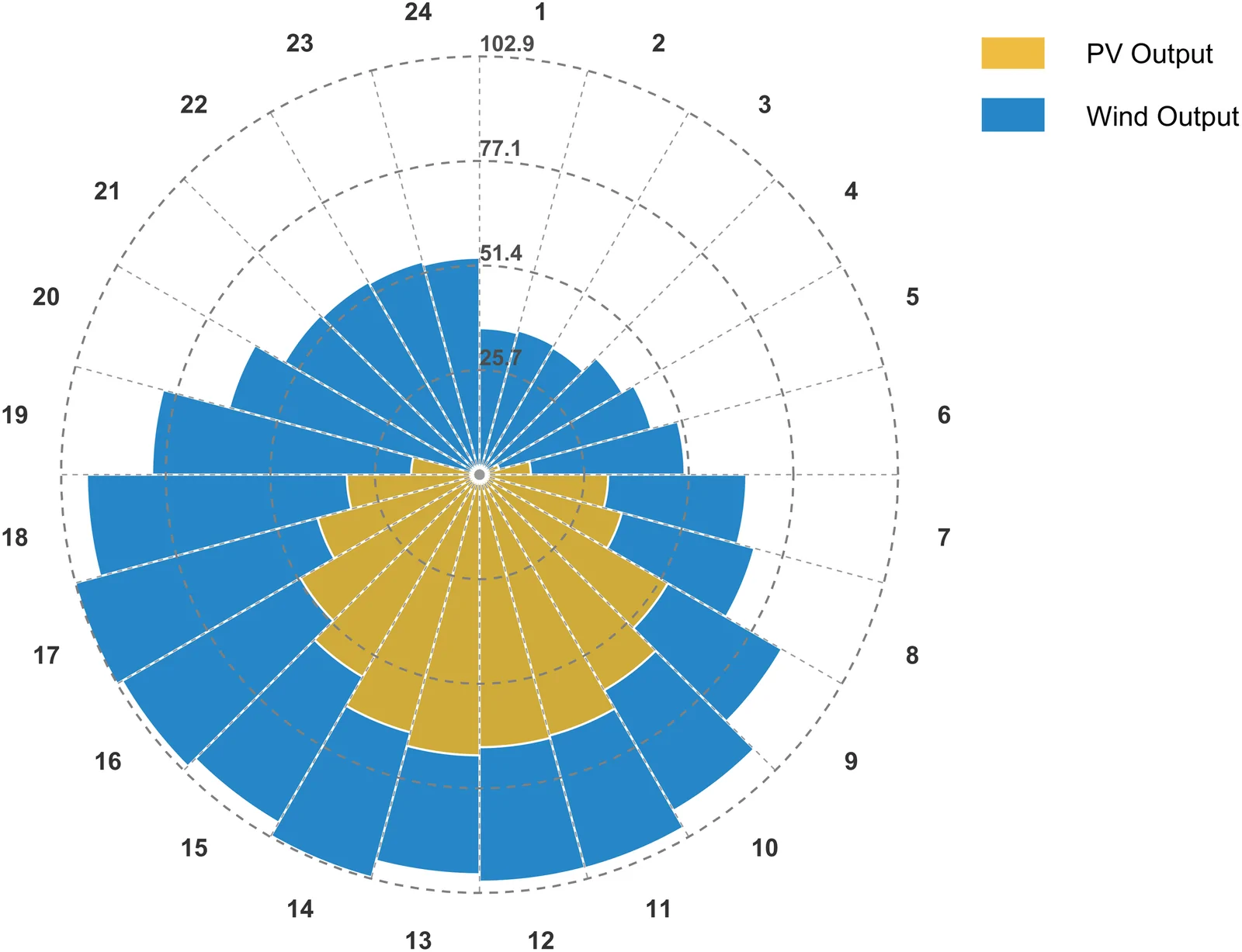
Polar chart of renewable generation.

**Fig 13:**
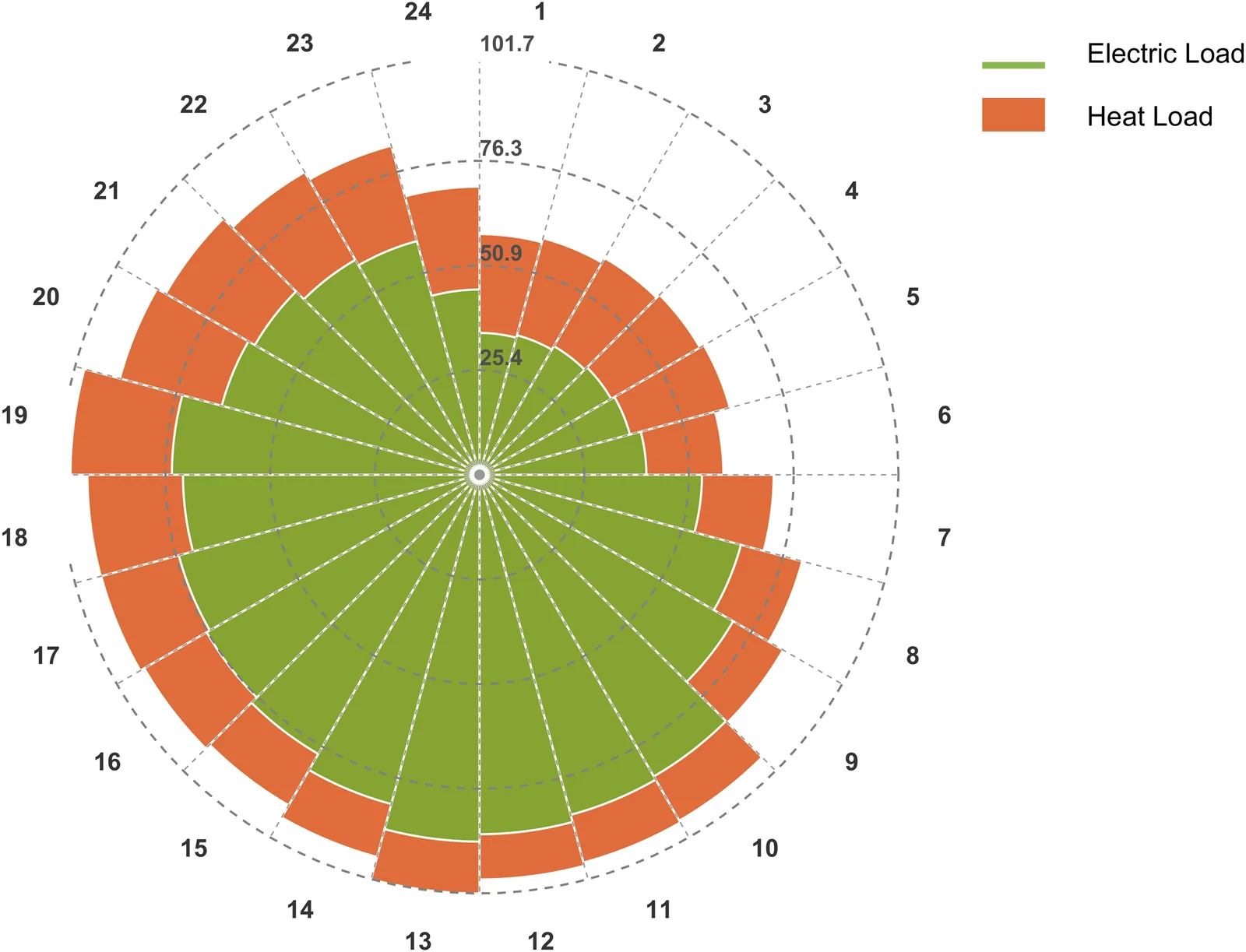
Polar chart of energy demands.

**Fig 14:**
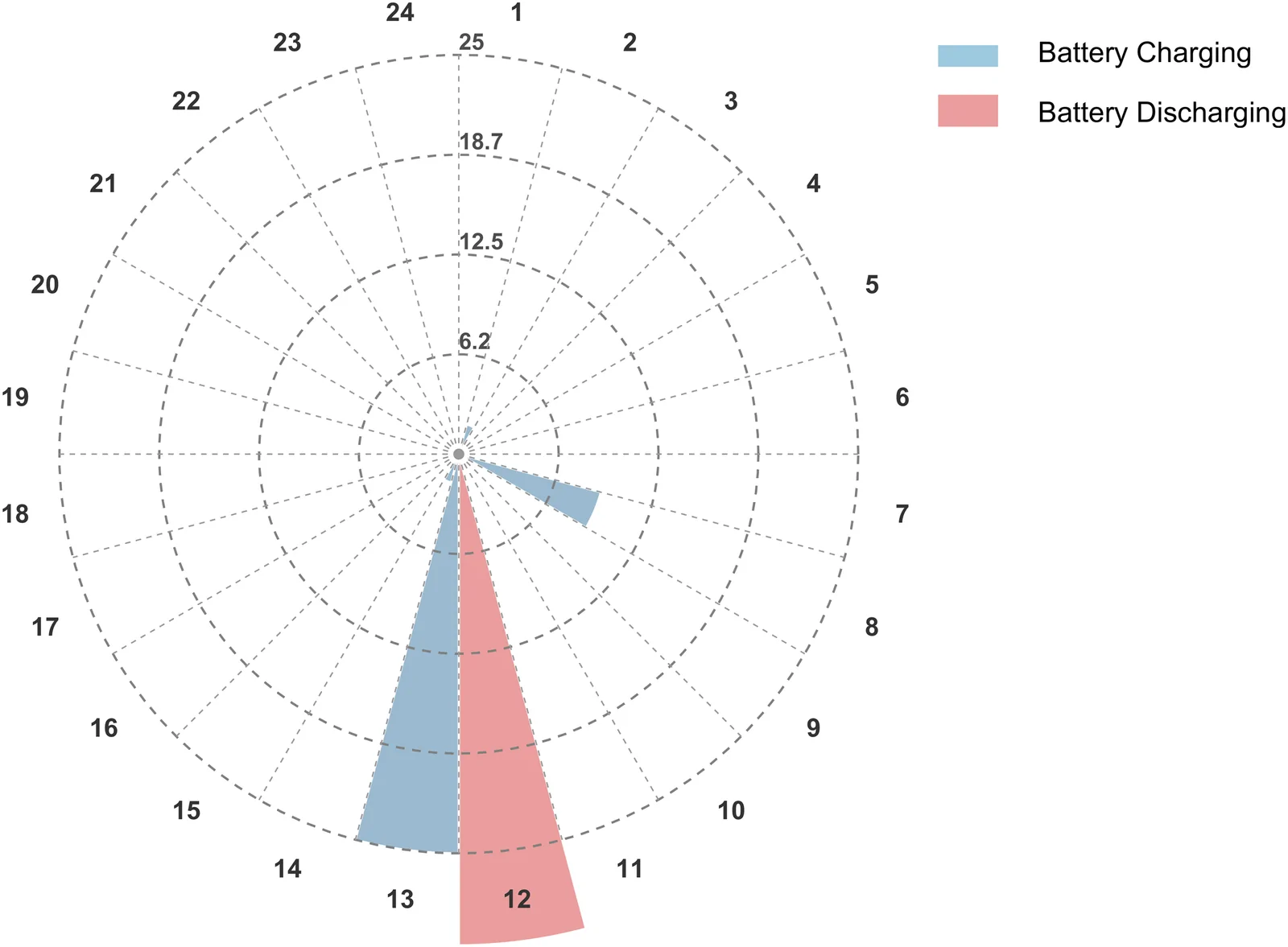
Polar chart of battery dynamics.

**Fig 15:**
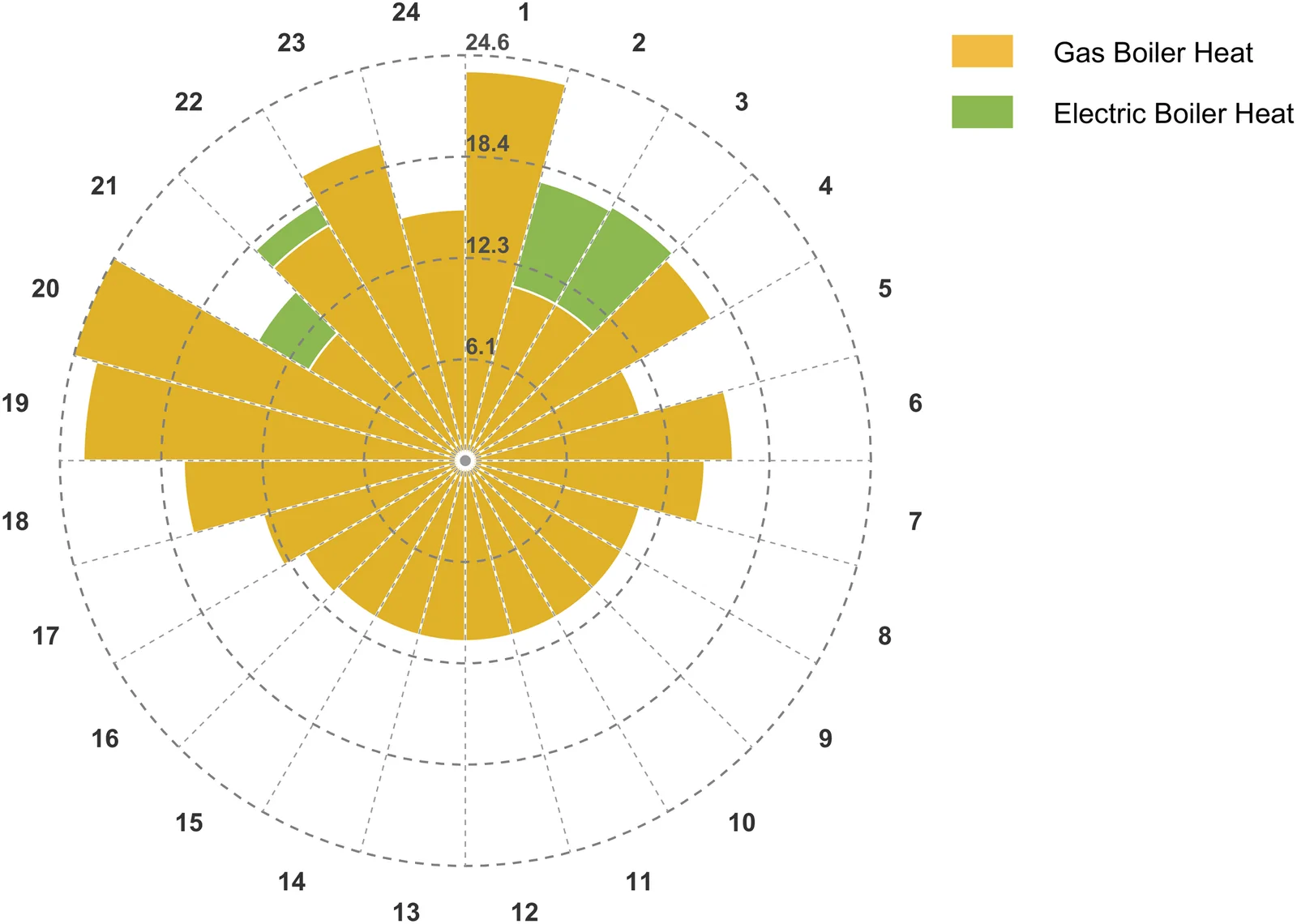
Polar chart of boiler heat production.

The corresponding polar-coordinate results are shown in Figs 12–15.

The polar-coordinate results in Figs 12–15 summarize the 24-h operating patterns of renewable generation, energy demand, battery operation, and boiler heat production. The plots show the temporal complementarity between wind and photovoltaic generation, the different peak periods of electrical and thermal demand, and the corresponding responses of storage and conversion equipment. These results provide an alternative visualization of the day-ahead schedule and its consistency with the diurnal source-load profiles.

### Model-predictive rolling tracking at the intra-day time scale

Building on the day-ahead schedule, the microgrid is subject to short-term renewable-generation and load forecast deviations. The intra-day MPC therefore updates the dispatch every 15 min to reduce the deviation from the day-ahead reference while satisfying the operating constraints.

**Fig 16:**
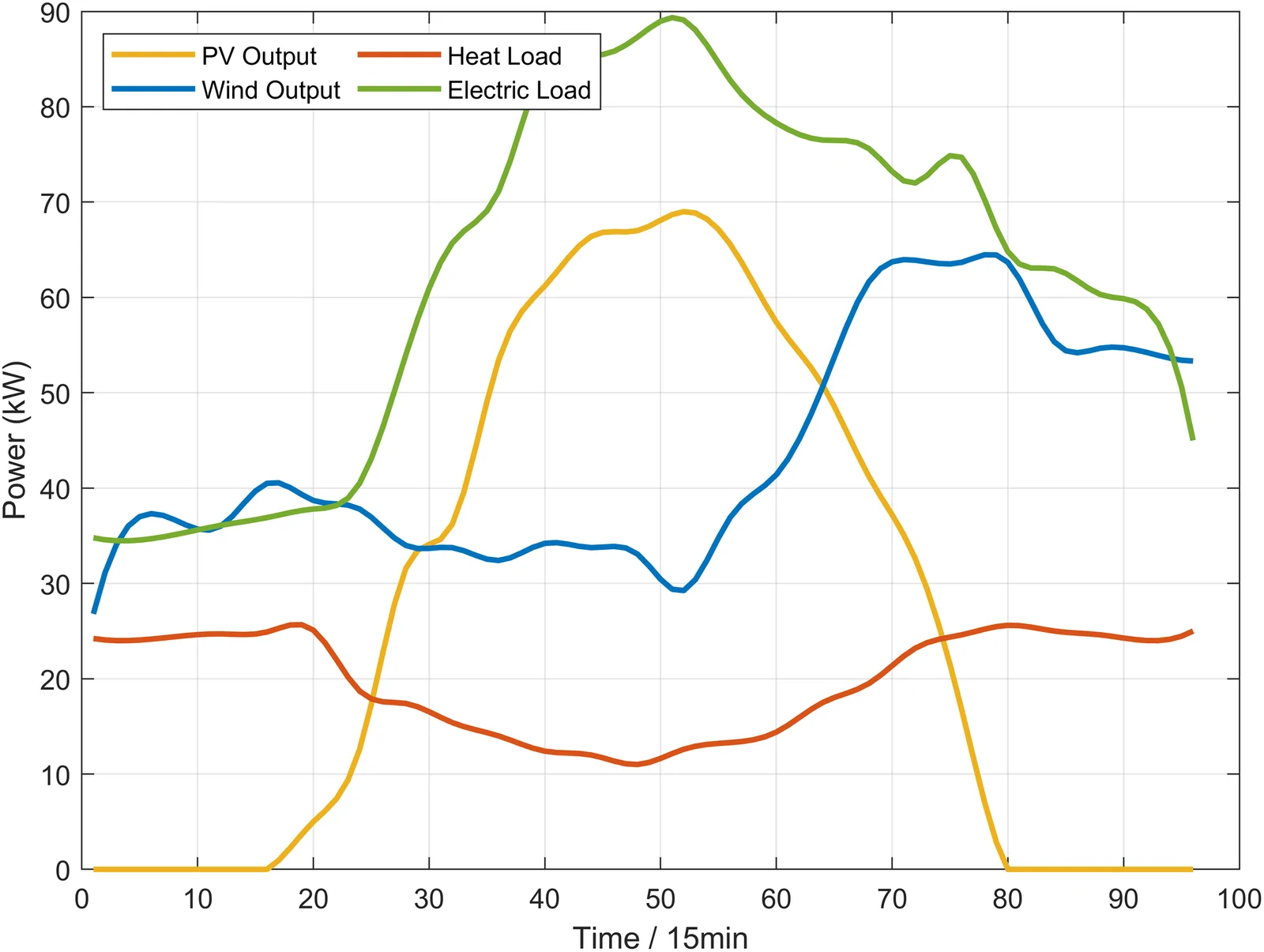
Intra-day source and load power operation curves.

Fig 16 presents the source and load trajectories at 15-min resolution. Relative to the hourly day-ahead profiles, wind and photovoltaic generation contain faster variations, while the electrical and thermal demands also deviate from their forecasts. These deviations create short-term electrical and thermal imbalances that must be corrected by the intra-day controller.

**Fig 17:**
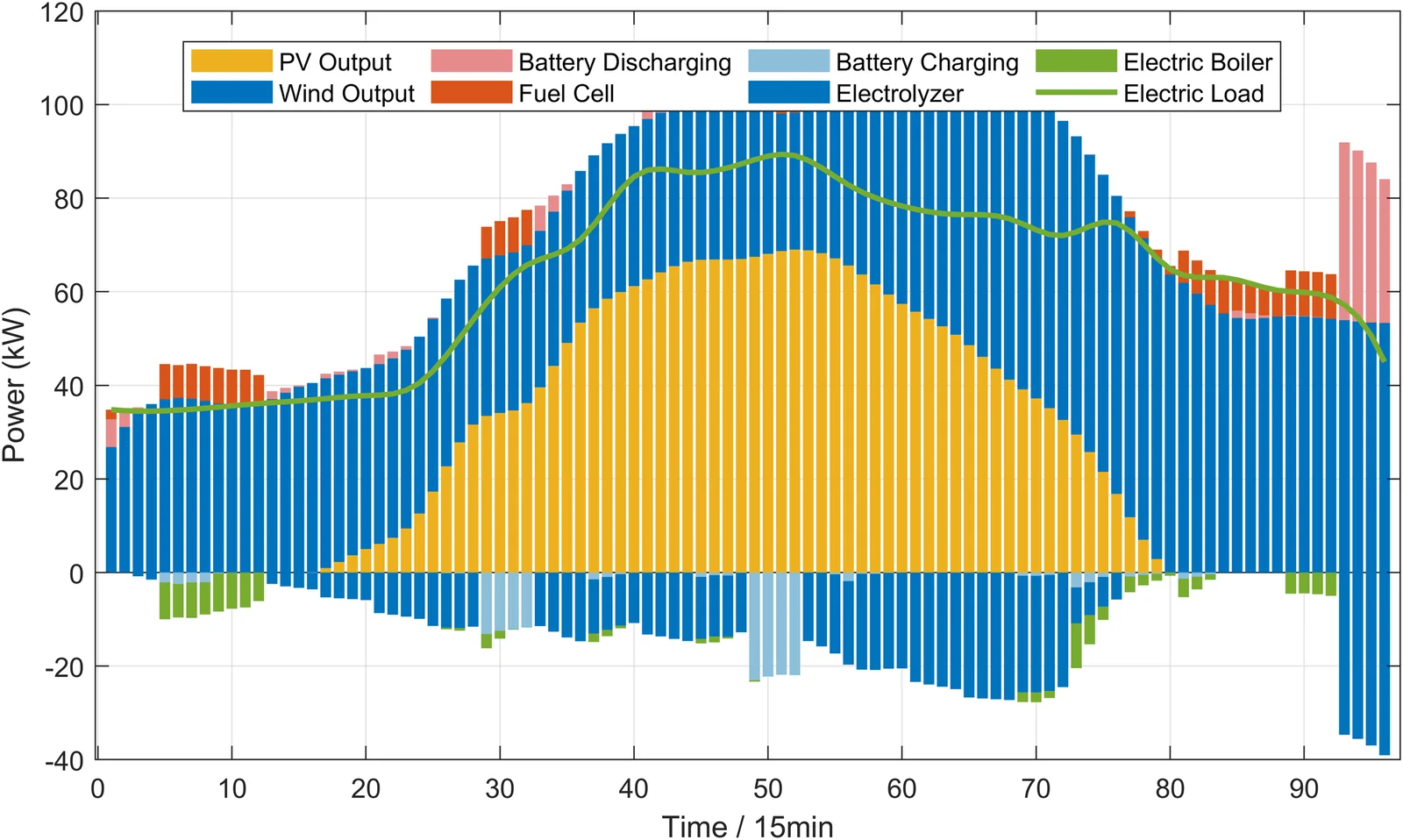
Intra-day electrical power balance of the microgrid.

**Fig 18:**
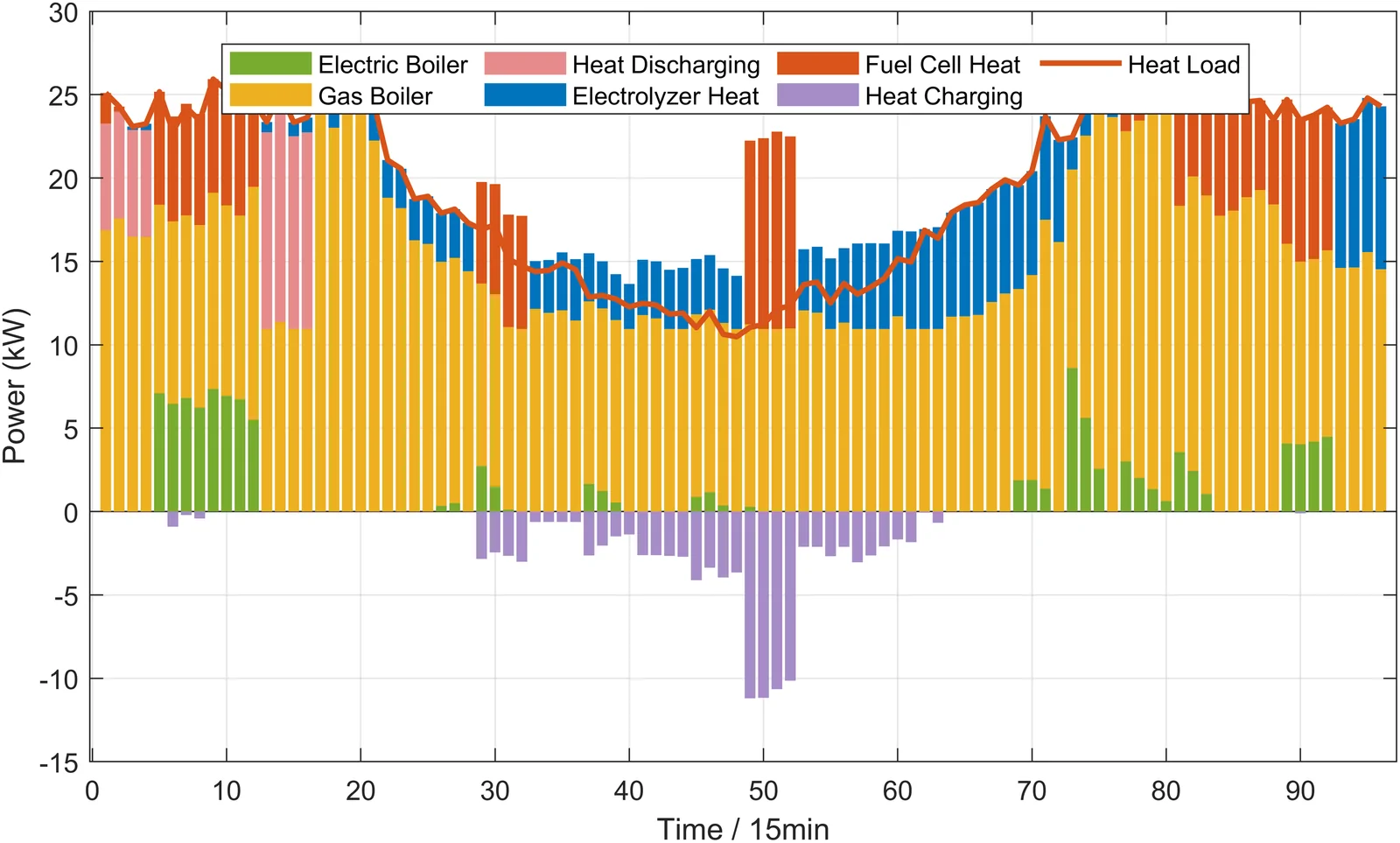
Intra-day thermal power balance of the microgrid.

Under the regulation of the intra-day model predictive control framework, the electrical power and thermal power balance states of the microgrid as shown in Figs 17 and 18 demonstrate the dynamic response capability of the underlying equipment to short-term prediction errors. During the real-time electrical power balancing process, when the system faces sudden short-term renewable energy output surpluses, the electrolyzer and the battery energy storage system rapidly increase their power absorption levels. Conversely, when encountering short-term power deficits, the fuel cell promptly increases its electrical energy output. During the real-time thermal power balancing process, the electric boiler and the thermal storage tank continuously correct their heat production and thermal charge and discharge plans based on the feedback information of the multi-step rolling prediction, ensuring the precise matching of multi-energy flows in the microgrid under complex uncertainty conditions.

**Fig 19:**
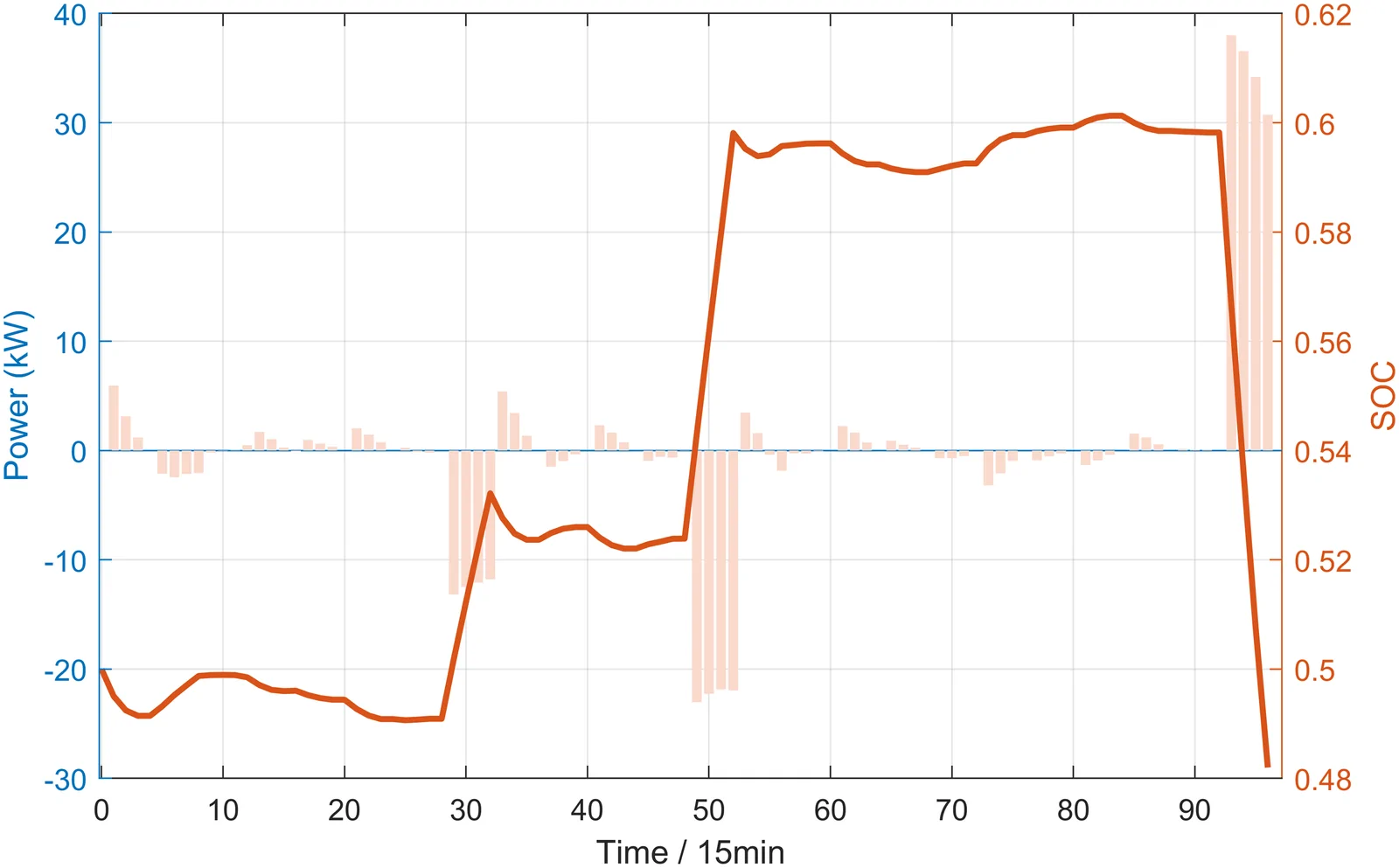
Intra-day battery state of charge.

**Fig 20:**
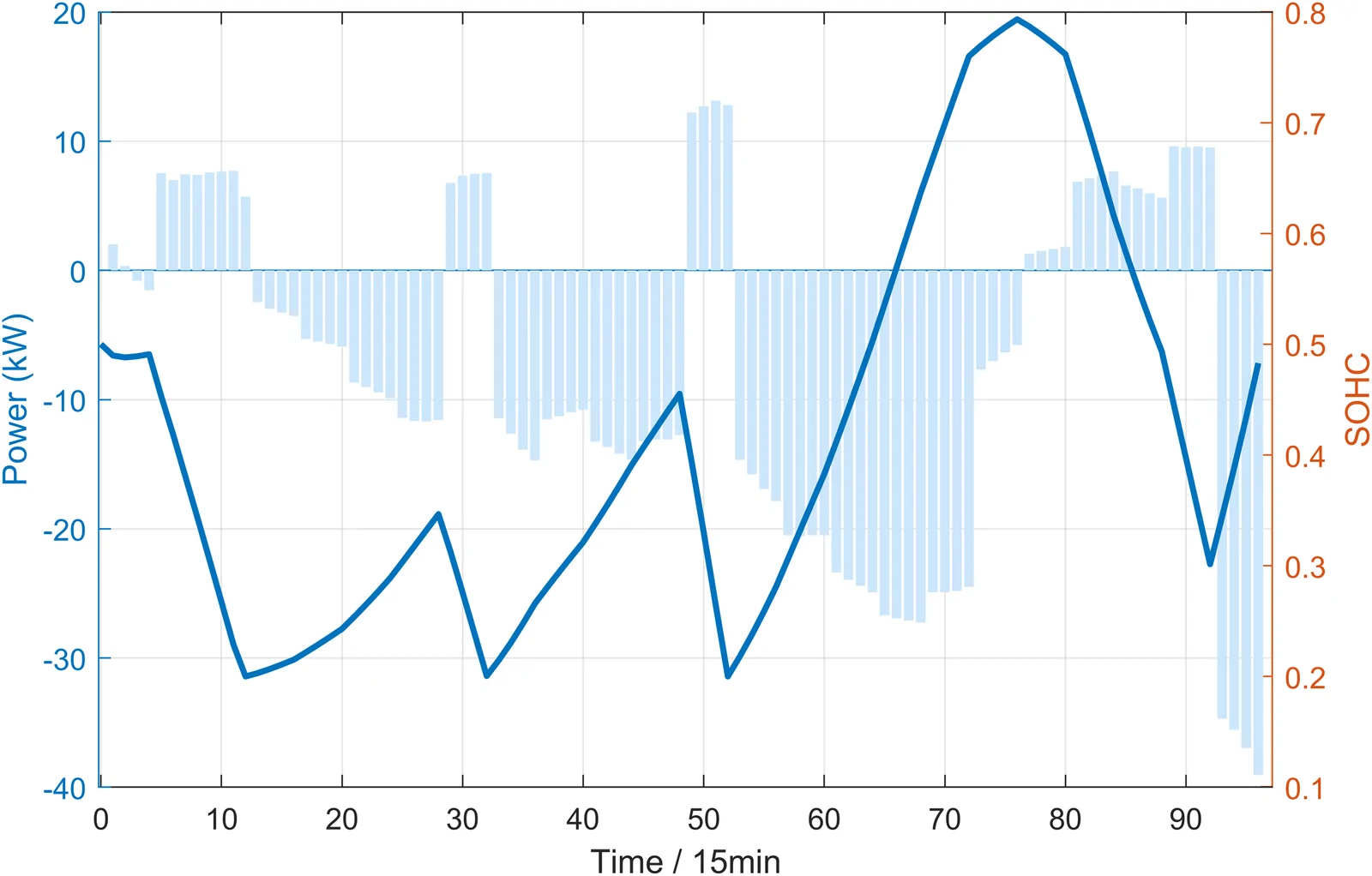
Intra-day state of hydrogen charge.

**Fig 21:**
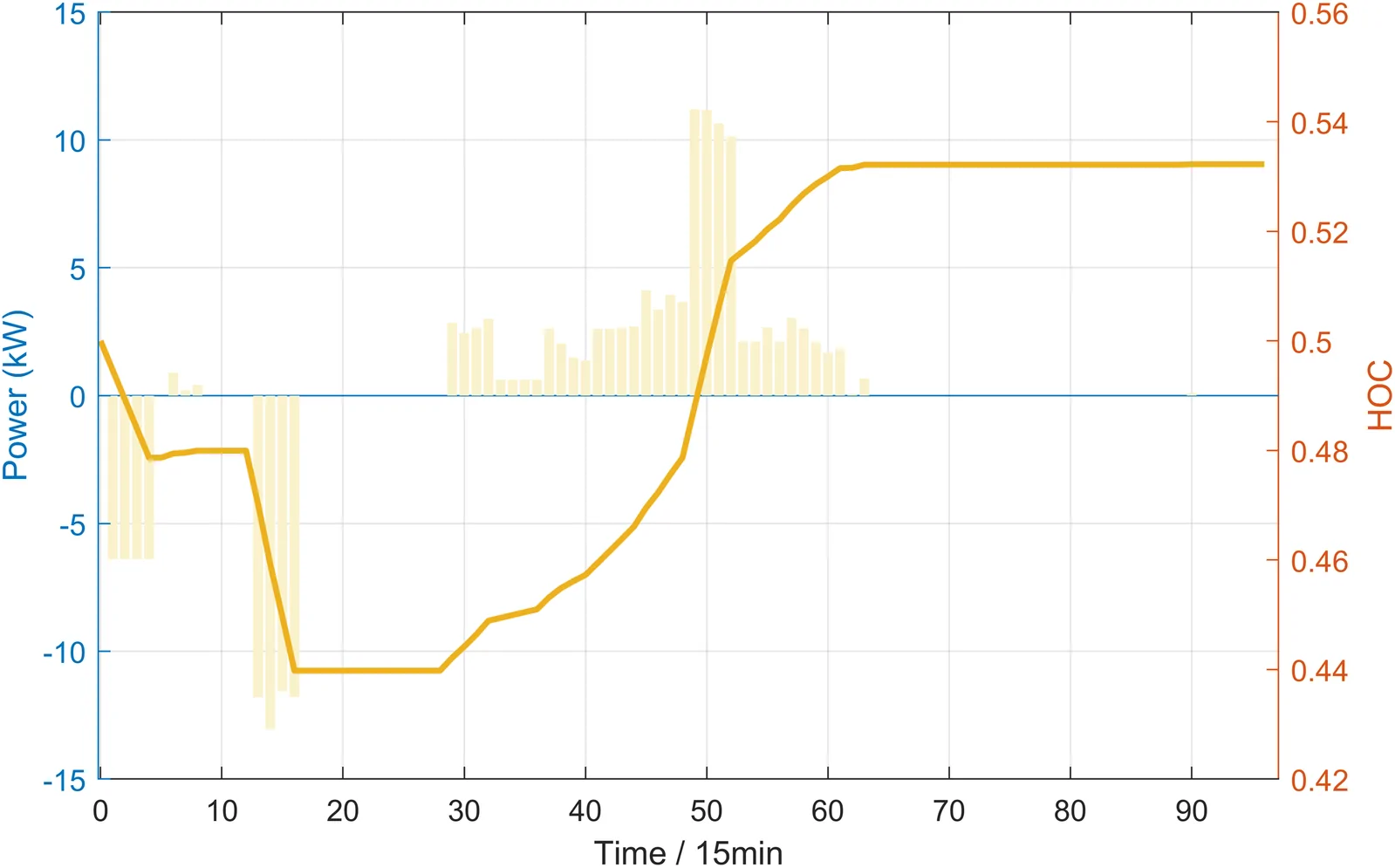
Intra-day state of heat charge.

**Fig 22:**
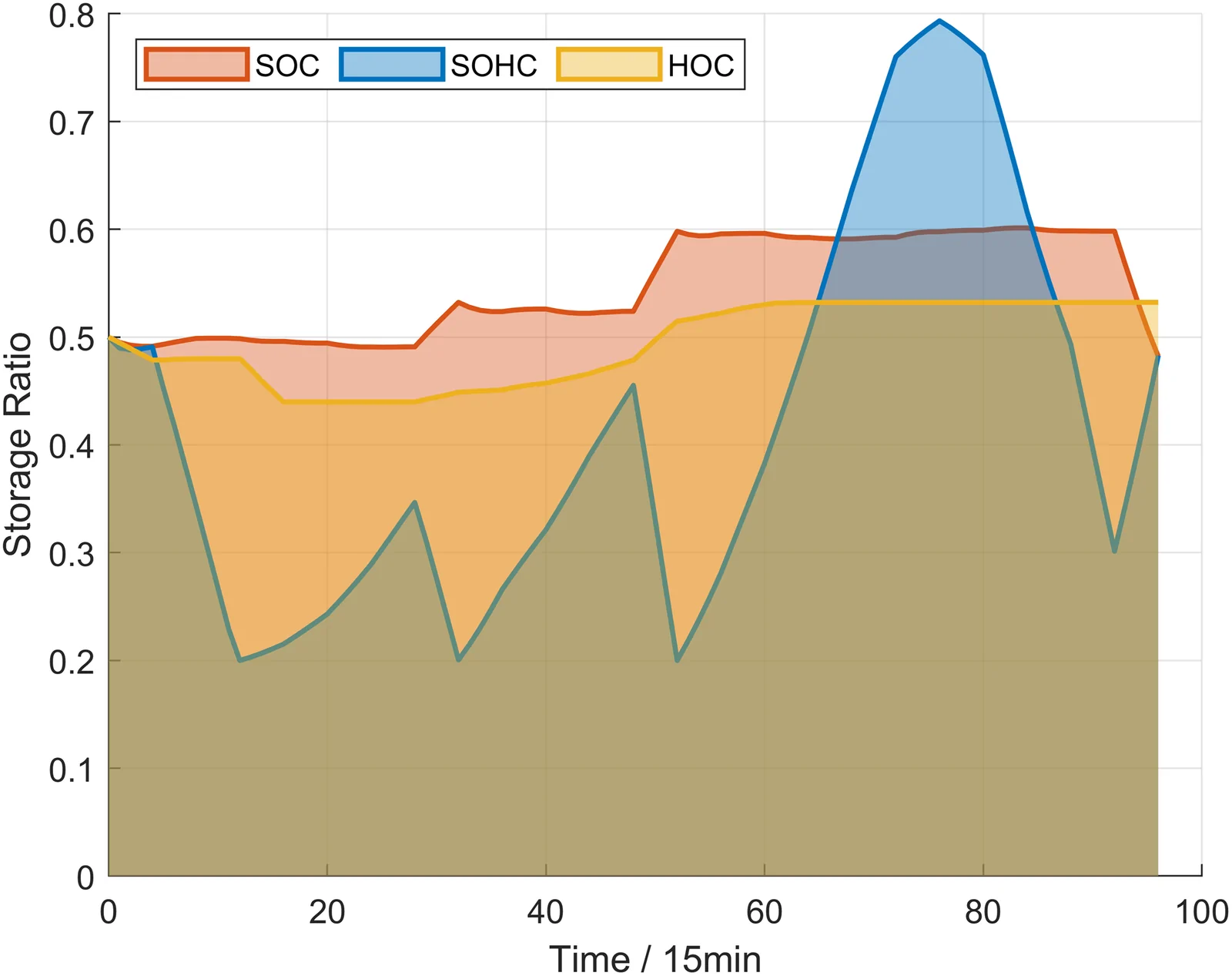
Intra-day coordinated energy-storage states.

Figs 19–22 show the intra-day responses of the battery, hydrogen storage, and thermal storage. The battery performs relatively frequent small adjustments, whereas the hydrogen state varies over a wider time interval and the thermal store compensates for heat-balance deviations. The combined state trajectories remain within their prescribed bounds and illustrate how the three storage media share short- and longer-duration correction tasks while following the day-ahead reference.

### Robustness under varying forecast-error levels

**Fig 23:**
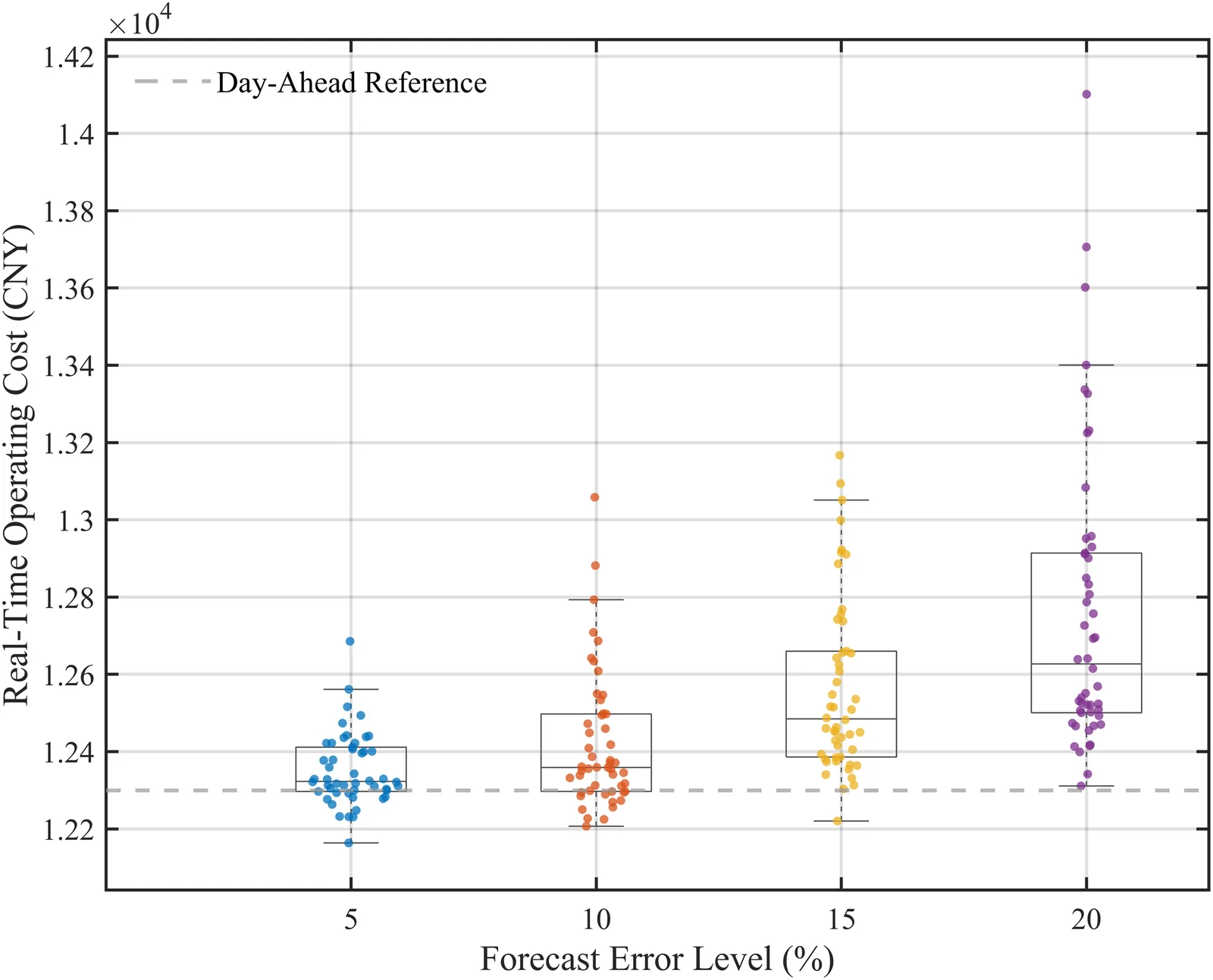
Distribution of real-time operating cost under different forecast-error levels.

To further evaluate the ability of the intra-day MPC to accommodate renewable-generation and load uncertainty, additional robustness tests are conducted at forecast-error levels of 5%, 10%, 15%, and 20%. The equipment parameters, market prices, MPC horizon, weighting matrices, and physical constraints remain unchanged across all cases. For each error level, 40 independent realizations are used. Wind, photovoltaic, electrical-load, and thermal-load forecasts are perturbed by zero-mean truncated-normal relative errors; the truncation bound equals the prescribed error level and the standard deviation is one-third of that bound. The same random-seed set is used at all four levels so that the dispersion changes are attributable to the error magnitude rather than to different random samples. In Fig 23, the colored points denote the operating costs obtained from individual runs, the horizontal line inside each box denotes the median, and the box and whiskers summarize the dispersion of the corresponding cost distribution. The dashed horizontal line represents the day-ahead reference operating cost.

As shown in Fig 23, the real-time operating costs at the 5% and 10% error levels remain concentrated near the day-ahead reference, indicating that the rolling controller can compensate for moderate deviations by coordinating the battery, hydrogen storage, thermal storage, conversion devices, and tie-line exchange. When the forecast-error level increases to 15%, both the median cost and the interquartile range rise, reflecting the additional corrective energy required to preserve multi-energy balance. At the 20% level, the distribution becomes noticeably wider and high-cost realizations occur more frequently, which indicates that severe forecast errors increase both the expected operating cost and its variability.

Nevertheless, the cost distribution exhibits an overall gradual upward shift rather than an abrupt discontinuity across the tested error levels. This result demonstrates that the proposed multi-time-scale scheduling framework retains useful economic robustness under moderate forecasting uncertainty. It also reveals the practical limitation of the nominal MPC formulation: under severe errors, larger corrective actions and more frequent use of grid electricity or dispatchable conversion equipment are required. Therefore, the robustness result complements the recursive-feasibility and practical-stability analysis by quantifying how uncertainty affects economic performance rather than claiming that the operating cost is invariant to forecasting errors.

### Extraction of typical scenarios and sensitivity analysis

**Fig 24:**
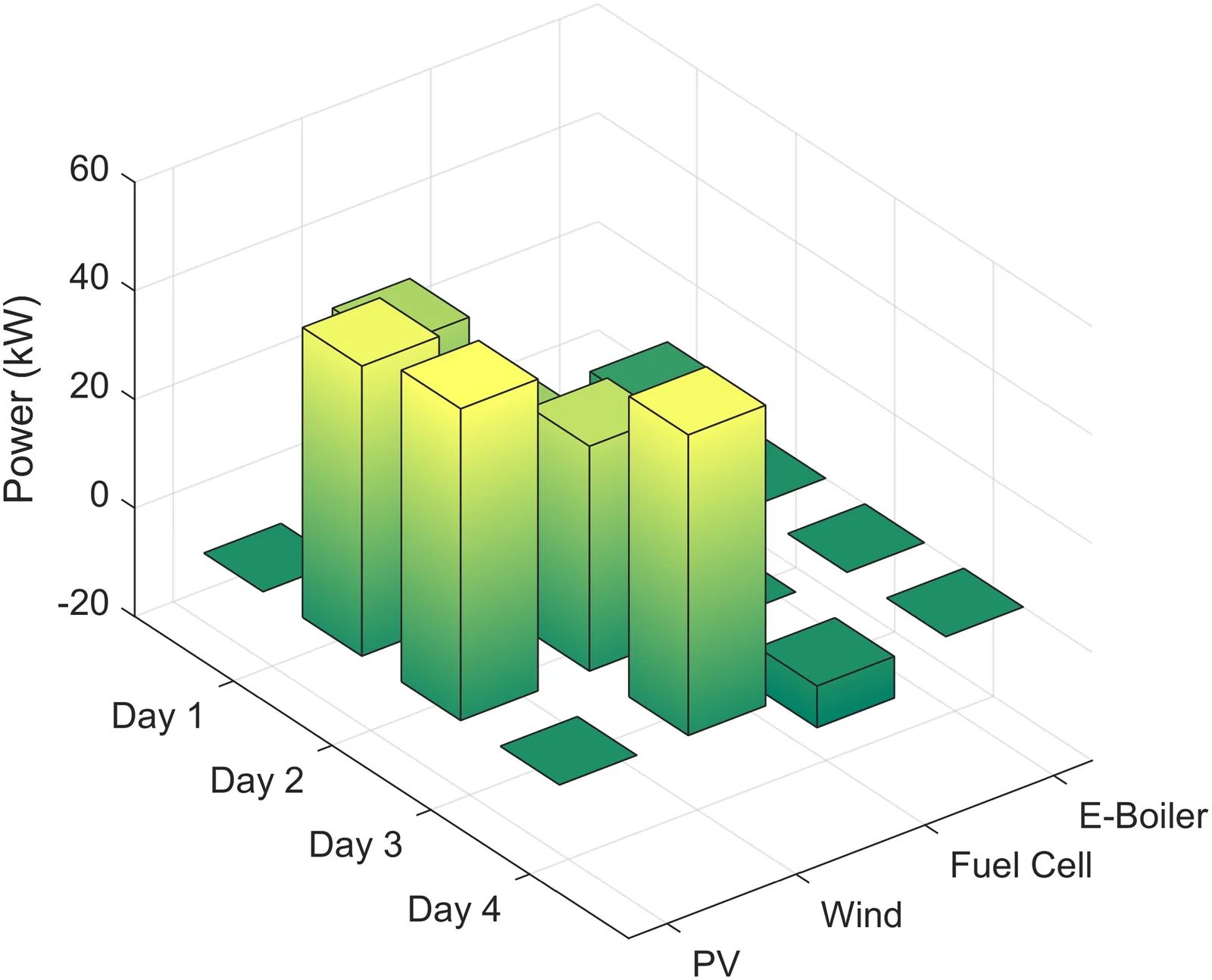
Power of supply equipment in typical scenarios.

**Fig 25:**
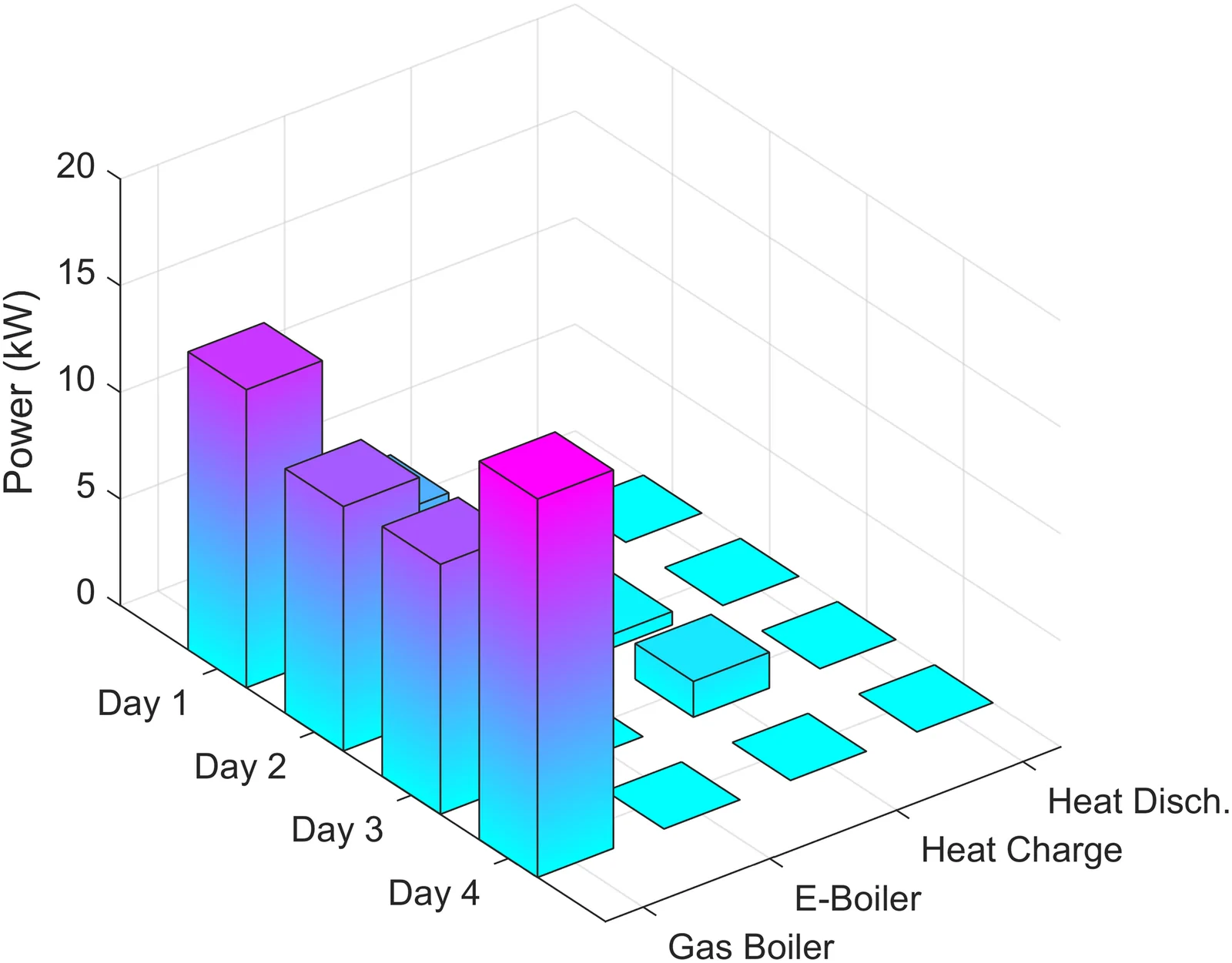
Power of thermal equipment in typical scenarios.

**Fig 26:**
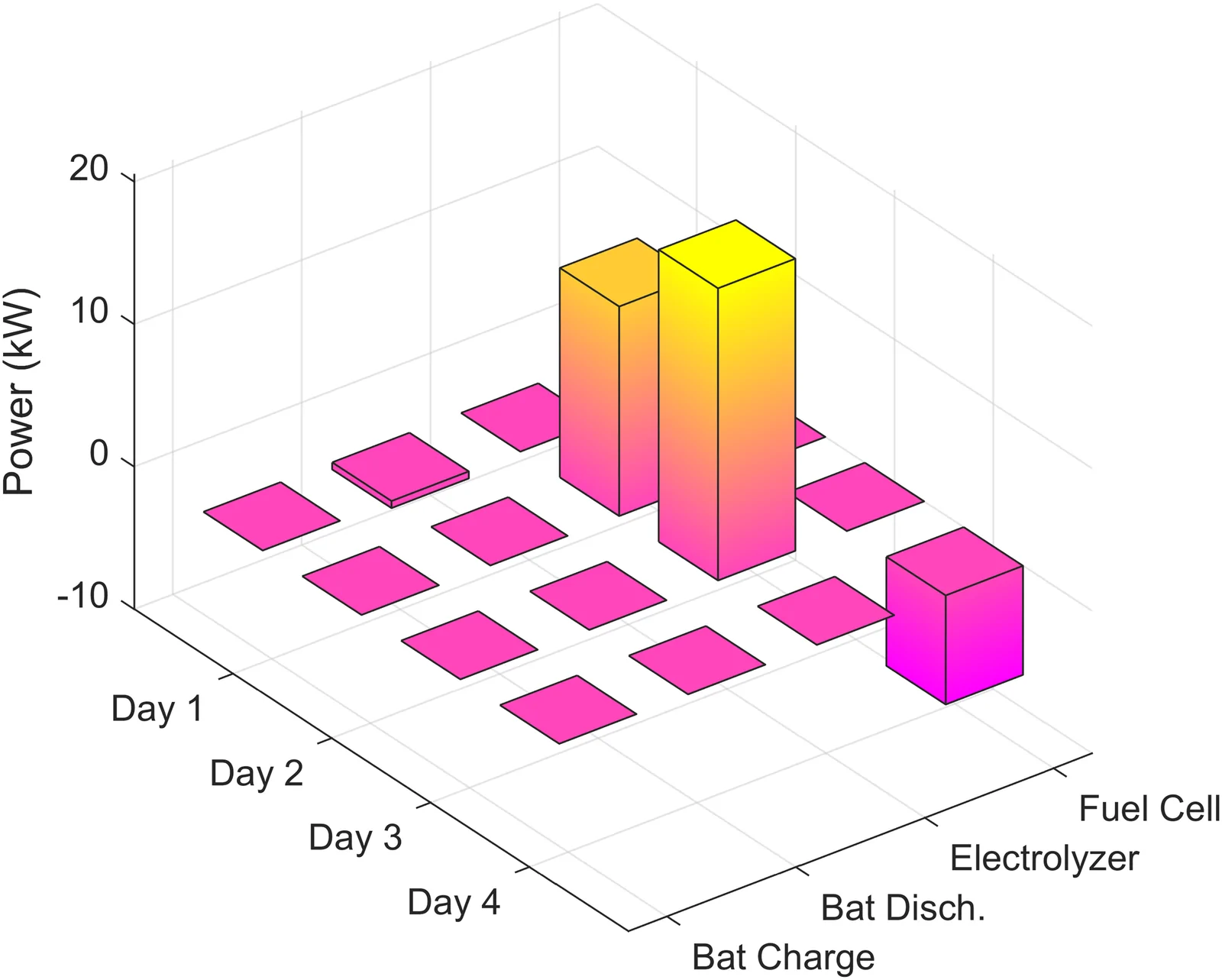
Storage and hydrogen response in typical scenarios.

**Fig 27:**
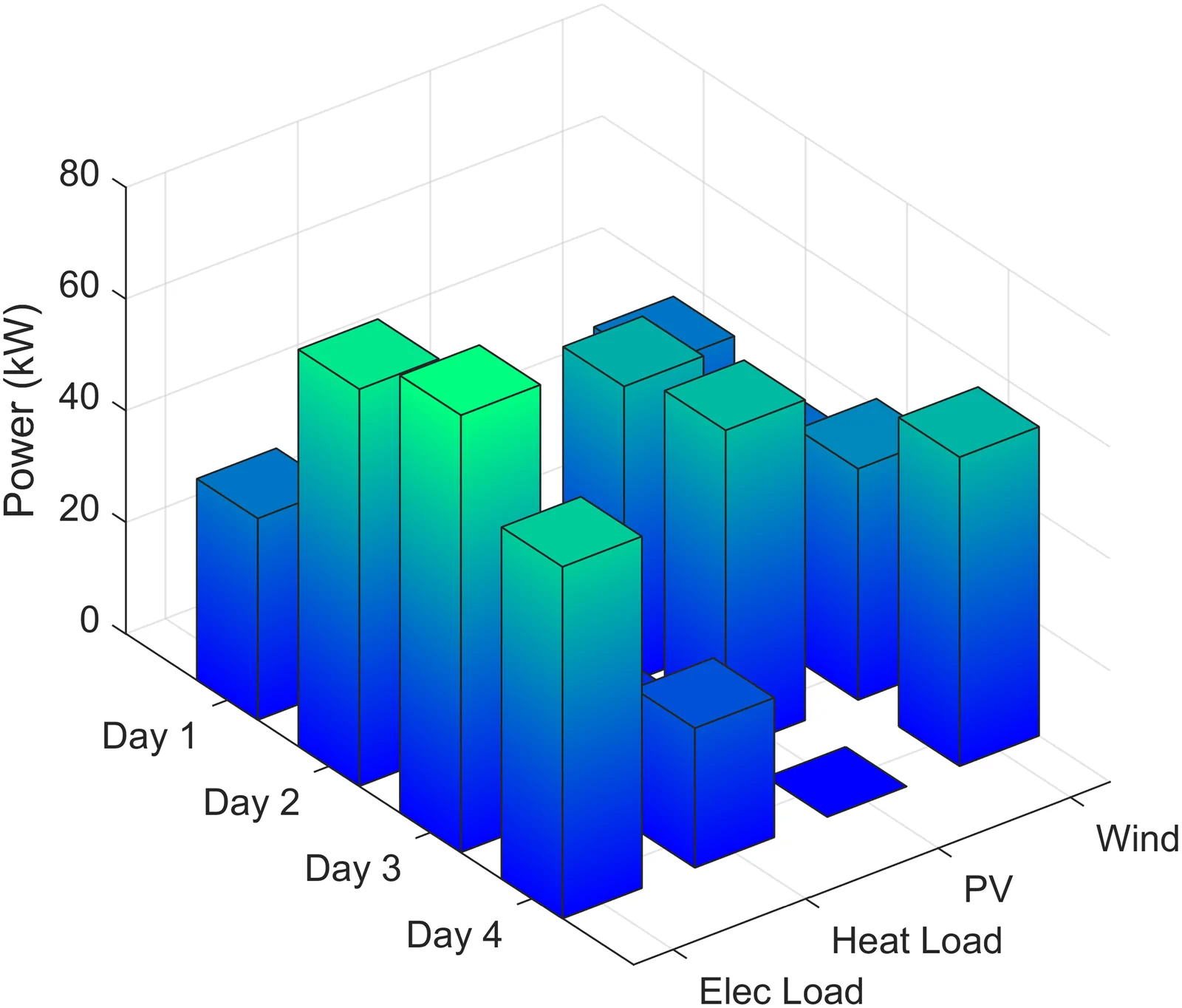
Source and load power distribution in typical scenarios.

Four representative operating days are compared to examine the scheduling response under different renewable-resource and load conditions. Figs 24–27 show the corresponding allocation of supply, conversion, and storage equipment. On days with lower renewable availability or higher thermal demand, the gas boiler and fuel cell provide more output. When renewable generation is abundant, the electrolyzer, electric boiler, and storage devices absorb a larger share of the available energy. Across the four cases, the schedules satisfy the energy-balance and equipment constraints while adapting the conversion and storage decisions to the net-load profile.

**Fig 28:**
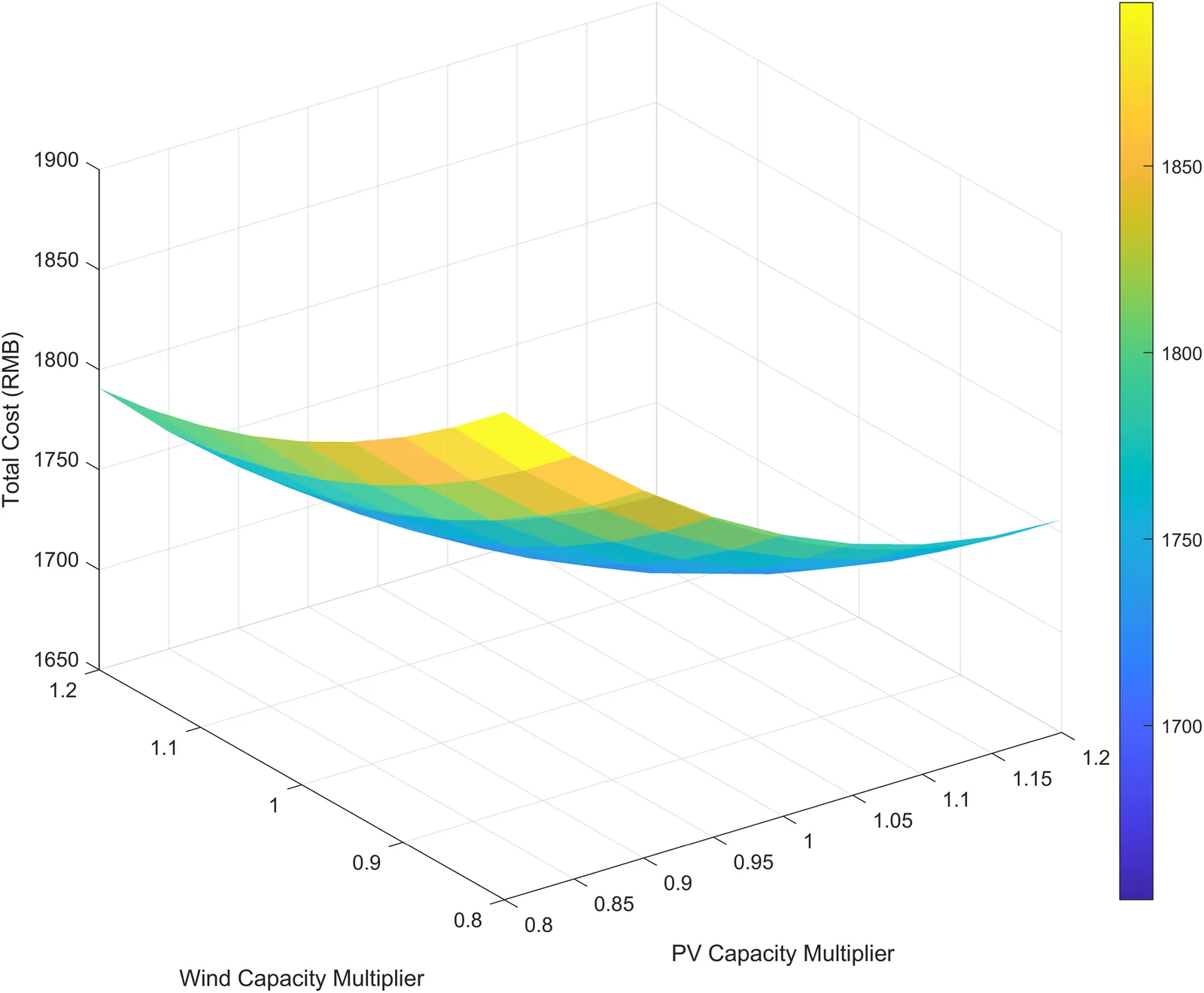
Sensitivity analysis of total cost to wind and PV capacity multipliers.

**Fig 29:**
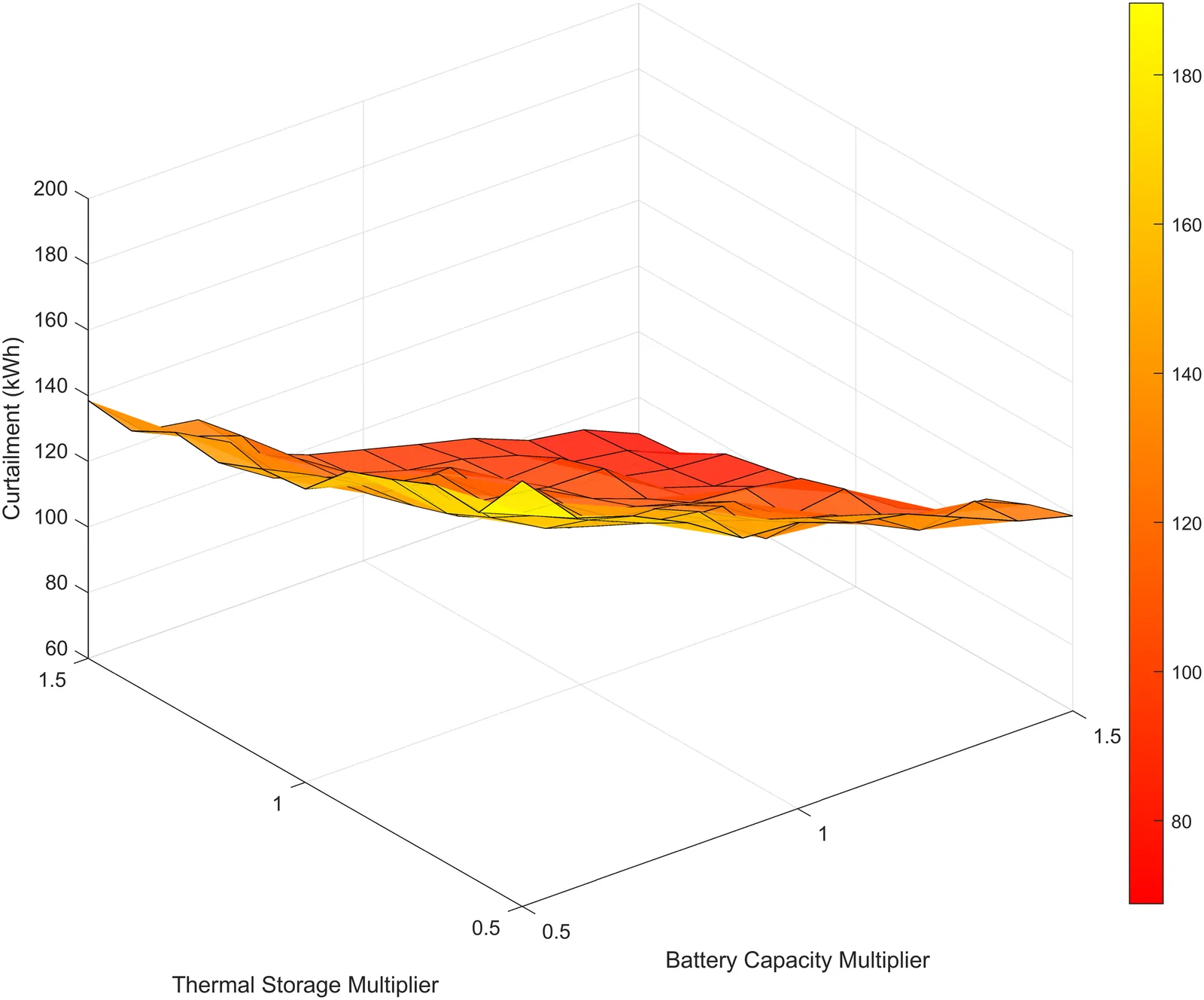
Sensitivity analysis of power curtailment to energy storage capacity multipliers.

A multivariable sensitivity analysis is conducted to examine how equipment-capacity bounds affect operating cost and renewable-energy curtailment. Fig 28 shows a low-cost region over the tested wind and photovoltaic capacity multipliers. Lower capacities increase reliance on grid imports and natural gas, whereas capacities beyond the low-cost region provide limited additional operating-cost benefit under the adopted price assumptions. Fig 29 shows that increasing battery and thermal-storage capacities initially reduces curtailment, followed by diminishing marginal improvement. The results provide a quantitative reference for preliminary capacity-screening decisions within the tested parameter range; they are not presented as a universal investment optimum.

### Privacy-preserving performance analysis

To evaluate the proposed strategy under the passive eavesdropping attack defined in Eqs (29)–(30), 30 operating days are considered, with 24 hourly periods for each day, resulting in 720 day–hour samples. The normalization constant is set to $\delta =10^{-8}$, and $P_{MG}^{\max}$ and $P_{MG}^{\min}$ are obtained from the unperturbed 30-day reference dataset. Three distributed interaction mechanisms are evaluated under identical source, load, market, equipment, penalty-parameter, and stopping-tolerance settings: noise-free ADMM, ADMM with fixed Laplace noise, and the proposed ADMM with dynamically decaying Laplace noise. For the fixed-noise benchmark, the constant scale is set to $b_{fix}=4.29$ kW, equal to the average value of the proposed sequence $\{b_{k}{\}}_{k=1}^{38}$, so that the comparison does not favor either privacy mechanism through an arbitrarily selected perturbation amplitude. For each sample, the complete ADMM communication trajectory is intercepted, the unperturbed boundary power is reconstructed using Eq (30), and the normalized reconstruction error and privacy leakage risk are then calculated using Eqs (32) and (33). Each cell in the calendar heatmaps represents the resulting $R_{d,t}$ value. A larger value means that the eavesdropper reconstructs the unperturbed boundary power more accurately and therefore corresponds to a higher privacy leakage risk.

The privacy parameters used for the reported experiment are $\epsilon_{0}=0.50$, *c* = 1.00, α=0.95, Δf=5.00 kW, and $K_{\max}=50$, as summarized in Table 3. Consistent with Fig 3, the executed distributed communication terminates after $K_{term}=38$ released iterates. Table 5 reports the normalized noise scale, the corresponding dimensional Laplace scale, the iteration-wise privacy budget, and the cumulative basic-composition bound up to the actual stopping iteration. The initial transmitted iterate uses $b_{1}=9.50$ kW, whereas the terminal transmitted iterate retains the positive scale $b_{38}=1.42$ kW. At termination, the single-release budget is $\epsilon_{38}=3.51$, and the total budget of the complete 38-iteration transcript is $\epsilon_{cum}(38)=60.23$.

**Table 5:** Noise scales and privacy-budget consumption up to the observed ADMM termination iteration.

Iteration *k*	$b_{k}/\Delta f$	$b_{k}$ (kW)	$\epsilon_{k}$	$\epsilon_{cum}(k)$
1	1.90	9.50	0.53	0.53
5	1.55	7.74	0.65	2.92
10	1.20	5.99	0.84	6.70
20	0.72	3.58	1.39	17.90
30	0.43	2.15	2.33	36.59
38*	0.28	1.42	3.51	60.23

* Actual termination iteration satisfying both residual tolerances.

These values make both the perturbation amplitude and the associated privacy loss directly reproducible. They show that the terminal boundary-power message is not noise free, but they also confirm that privacy protection becomes weaker in the later iterations and that privacy loss accumulates across repeated releases. The cumulative value in Table 5 counts only the 38 iterates released before the stopping rule is met; the offline continuation displayed after the termination marker in Fig 3 is not transmitted and does not consume additional privacy budget. The maximum-iteration safeguard is set to $K_{\max}=50$, whereas the proposed run terminates at $K_{term}=38$ because both residual tolerances are satisfied. Hence, only the 38 transmitted iterates enter the reported cumulative privacy budget. Therefore, the privacy claim in this paper is restricted to a finite-iteration, reconstruction-risk reduction result under the stated eavesdropping model; it should not be interpreted as uniform strong privacy for an indefinitely long iteration sequence.

#### Benchmark comparison and cleared-cost optimality loss.

To provide a fair benchmark beyond the comparison with an unprotected interaction mechanism, the proposed dynamically decaying-noise ADMM is compared with a conventional fixed-Laplace-noise ADMM and the noise-free ADMM. A centralized optimization model using the complete system information is additionally solved as the economic reference. All four cases use the same forecasts, equipment limits, electricity and carbon prices, grid-emission factor, and solver settings. The noise-based cases are independently repeated 30 times, and their reported costs and cost deviations are the corresponding sample means and standard deviations.

Let $F_{cen}$ denote the cleared cost of the centralized benchmark, $F_{m}$ the cleared cost of method *m*, and $F_{ADMM}$ the cost obtained by the noise-free distributed ADMM. The optimality gap relative to the centralized benchmark and the additional loss caused by noise are evaluated using Eq (49), which has been defined in the methodology section. Table 6 shows that the noise-free ADMM remains close to the centralized benchmark, with a cost gap of 0.12%. The fixed-noise method lowers the mean leakage risk to 0.58, but its persistent perturbation increases the cleared cost to 12,531.86 CNY, corresponding to a 2.85% gap from the centralized result and a 2.73% loss relative to the noise-free ADMM. In comparison, the proposed method obtains a cleared cost of 12,299.14 CNY. Its centralized cost gap is 0.94%, and the privacy-induced loss relative to the noise-free ADMM is limited to 0.82%.

**Table 6:** Comparison of cleared cost, privacy risk, and computational performance under different optimization methods.

Method	Cleared cost (CNY)	Gap to centralized (%)	Noise-induced loss (%)	Mean leakage risk	Cost SD (%)	Iterations	Time (s)
Centralized optimization	12,184.60	0.00	–	–	–	–	6.4
ADMM without noise	12,199.22	0.12	0.00	0.86	0.08	24	7.9
ADMM with fixed Laplace noise	12,531.86	2.85	2.73	0.58	1.25	50	13.8
Proposed dynamic-noise ADMM	12,299.14	0.94	0.82	0.67	0.46	38	10.6

**Fig 30:**
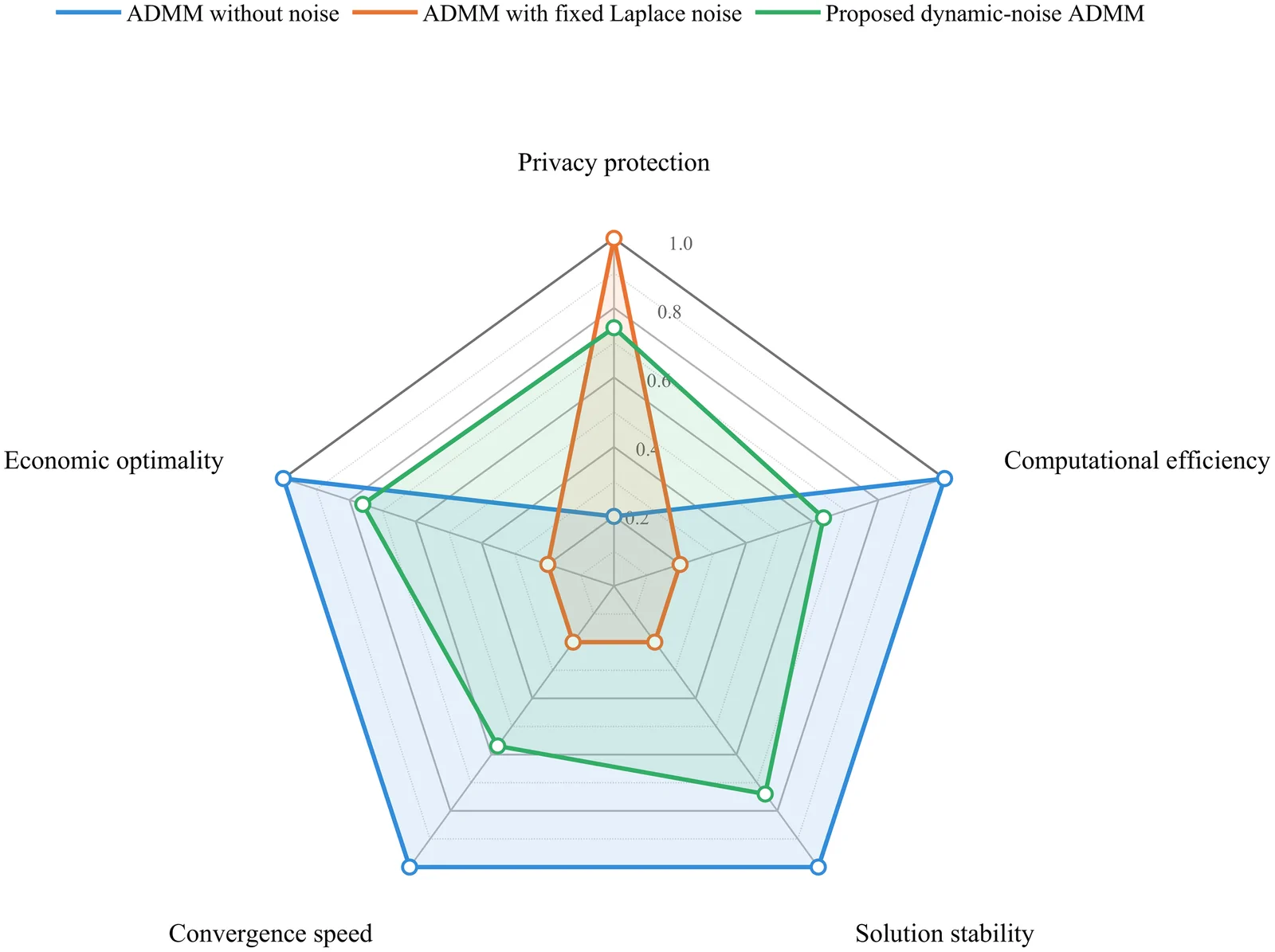
Radar-chart comparison of the noise-free ADMM, fixed-Laplace-noise ADMM, and proposed dynamic-noise ADMM.

Fig 30 summarizes the multidimensional comparison of the three distributed methods in terms of privacy protection, economic optimality, convergence speed, solution stability, and computational efficiency. For reproducibility, the underlying cost-type indicators are transformed into larger-is-better scores using Eq (50), which is defined before the simulation section. Here, $x_{m,j}$ is the raw value of indicator *j* for method *m*. The offset of 0.20 prevents the lowest score from collapsing at the origin and does not change the ranking. The fixed-noise method provides the strongest privacy protection, but its larger cost deviation, slower convergence, and higher computational burden reduce its overall performance. The noise-free ADMM performs best in economic optimality and computational efficiency but exhibits the highest leakage risk. The proposed method lies between these two extremes and provides a more balanced privacy–optimality trade-off.

**Fig 31:**
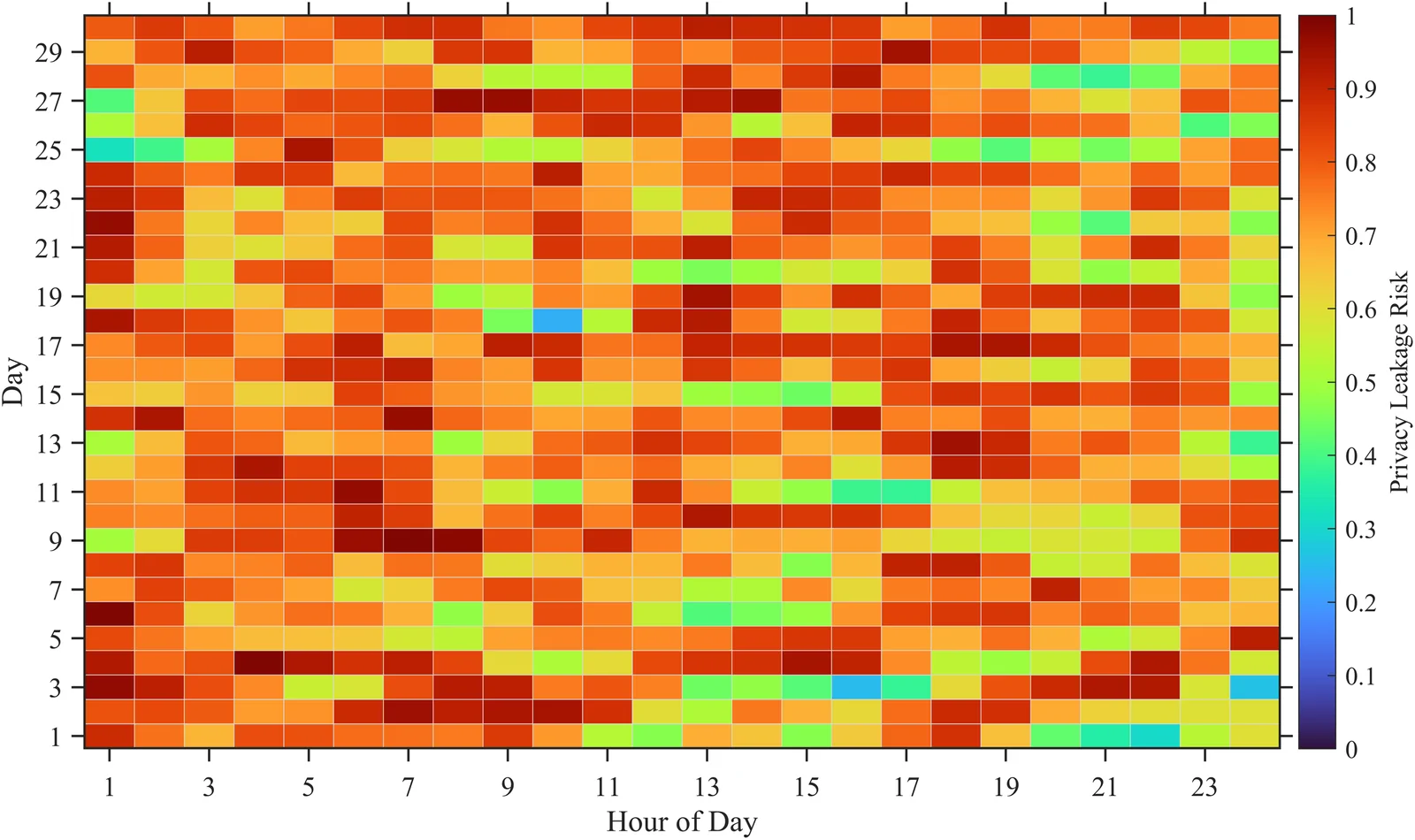
Privacy leakage risk with the proposed privacy-preserving strategy.

Fig 31 shows the privacy leakage risk distribution under the proposed privacy-preserving distributed optimization strategy. It can be observed that the risk values are no longer concentrated in the high-risk region. Several low-risk and medium-risk areas appear during different time periods, indicating that the dynamic Laplace noise mechanism weakens the temporal correlation and reconstruction regularity of the exchanged boundary power. Therefore, the proposed strategy can effectively reduce the exposure of sensitive operation information while maintaining the distributed clearing process.

**Fig 32:**
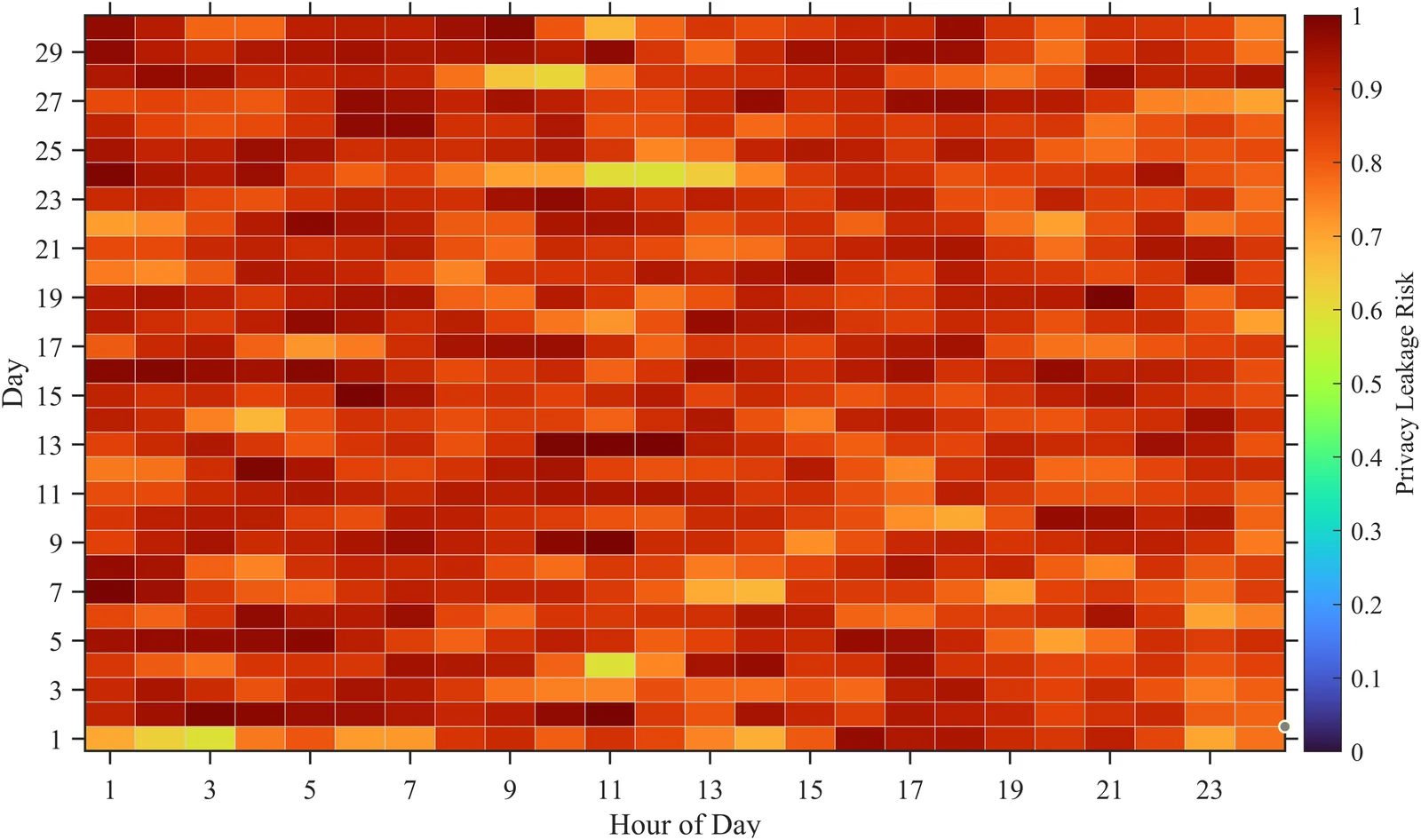
Privacy leakage risk without privacy-preserving protection.

In contrast, Fig 32 presents the privacy leakage risk without adopting the proposed privacy-preserving mechanism. Most calendar blocks remain in the orange-red and dark-red regions, which means that the boundary power information retains strong temporal regularity and can be more easily inferred from the exchanged data. This result indicates that direct information exchange in the distributed optimization process may still expose sensitive microgrid operation characteristics, even if the original equipment parameters and cost functions are not directly uploaded.

To quantitatively compare the three distributed interaction mechanisms, the mean, maximum, minimum, and standard deviation are calculated from the 720 values in each privacy leakage risk matrix according to Eqs (34) and (35). The mean values reported in Table 7 are 0.67 for the proposed strategy, 0.58 for the fixed-Laplace-noise benchmark, and 0.86 for the unprotected mechanism. The high-, medium-, and low-risk proportions are determined by counting the samples in the three intervals specified after Eq (36) and dividing the corresponding counts by 720. The fixed-noise benchmark achieves the lowest reconstruction risk because its perturbation does not decay, but Table 6 shows that this gain is accompanied by a larger cleared-cost loss and slower convergence. The proposed method reduces the mean leakage risk from 0.86 to 0.67 and decreases the proportion of high-risk periods from 82.36% to 43.75%, while retaining substantially lower economic and computational penalties than the fixed-noise benchmark.

**Table 7:** Comparison of privacy leakage risk under different interaction mechanisms.

Indicator	Proposed Strategy	Fixed Laplace Noise	Without Protection
Mean privacy leakage risk	0.67	0.58	0.86
Maximum privacy leakage risk	0.97	0.91	1.00
Minimum privacy leakage risk	0.22	0.18	0.58
Standard deviation of risk	0.16	0.18	0.08
High-risk periods above 0.80	43.75%	17.78%	82.36%
Medium-risk periods between 0.50 and 0.80	45.14%	50.00%	17.08%
Low-risk periods below 0.50	11.11%	32.22%	0.56%

Overall, the expanded benchmark comparison shows that no single method dominates every indicator. The noise-free ADMM achieves the smallest distributed cleared cost and the fastest convergence, but it retains the highest reconstruction risk. The fixed-Laplace-noise method provides stronger privacy protection, whereas its persistent disturbance leads to the largest cost gap, solution variability, iteration count, and computation time. The proposed dynamically decaying mechanism reduces the mean leakage risk relative to the noise-free case while limiting the noise-induced cost loss to 0.82%. Therefore, its principal advantage is a more balanced compromise between boundary-information protection and clearing optimality rather than the best isolated value of every metric.

#### Matched-privacy and matched-cost comparison.

**Fig 33:**
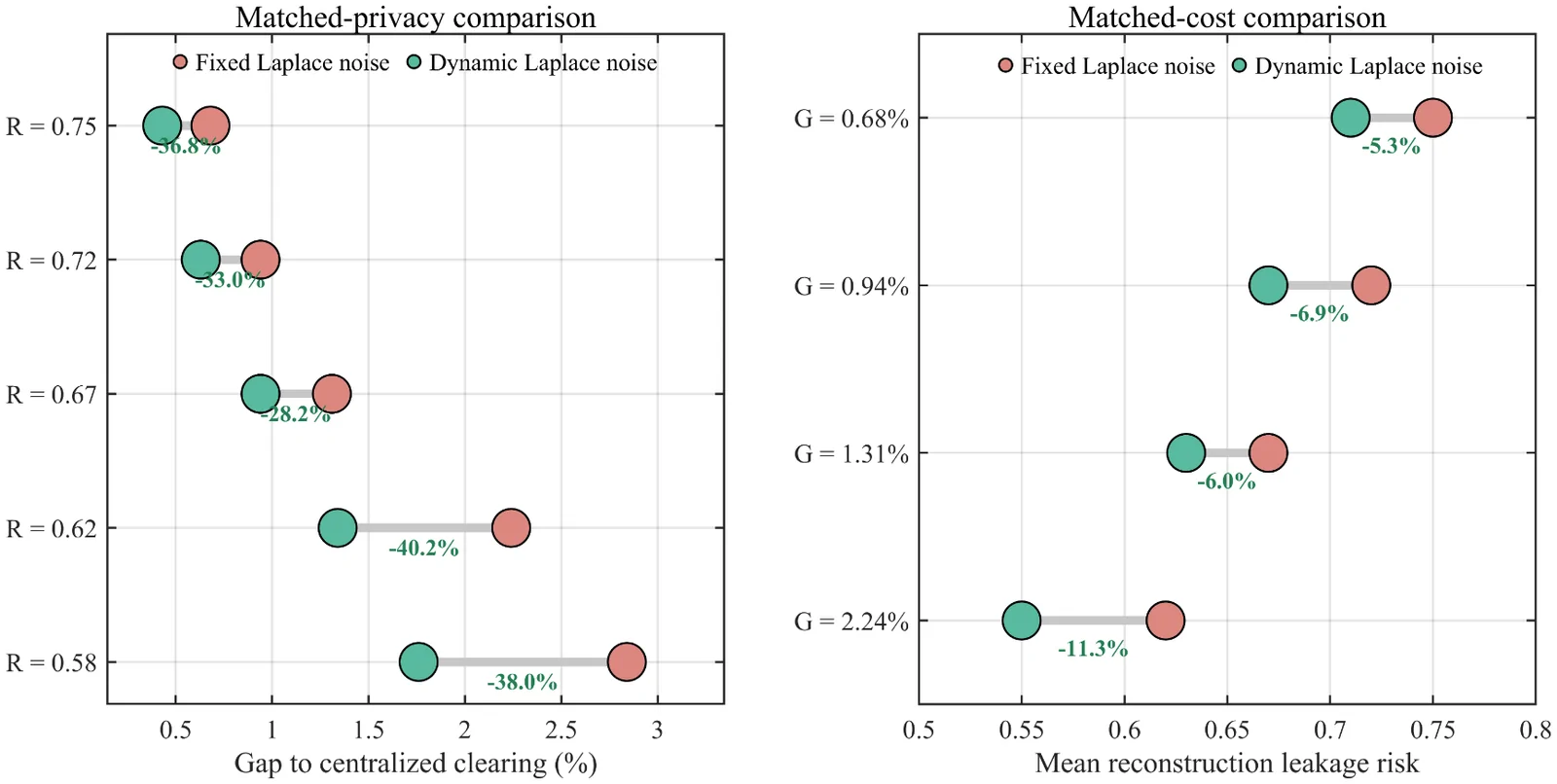
Matched-condition comparison of fixed and dynamically decaying Laplace-noise ADMM.

The average-noise benchmark is retained as a reference case, while an additional parameter sweep is used to compare the two perturbation mechanisms under matched operating conditions. Two complementary comparisons are considered. In the matched-privacy comparison, the mean reconstruction leakage-risk score $\bar{R}$ is fixed and the centralized clearing-cost gap is compared. In the matched-cost comparison, the centralized cost gap is fixed and the corresponding reconstruction-risk scores are compared. The complete numerical results are summarized in Table 8 and Fig 33.

**Table 8:** Matched-condition comparison of fixed and dynamically decaying Laplace noise.

Matched condition	Fixed noise	Dynamic noise	Reduction (%)
Privacy, $\bar{R}=0.75$	0.68% gap	0.43% gap	36.8
Privacy, $\bar{R}=0.72$	0.94% gap	0.63% gap	33.0
Privacy, $\bar{R}=0.67$	1.31% gap	0.94% gap	28.2
Privacy, $\bar{R}=0.62$	2.24% gap	1.34% gap	40.2
Privacy, $\bar{R}=0.58$	2.84% gap	1.76% gap	38.0
Cost, *G* = 0.68%	$\bar{R}=0.75$	$\bar{R}=0.71$	5.3
Cost, *G* = 0.94%	$\bar{R}=0.72$	$\bar{R}=0.67$	6.9
Cost, *G* = 1.31%	$\bar{R}=0.67$	$\bar{R}=0.63$	6.0
Cost, *G* = 2.24%	$\bar{R}=0.62$	$\bar{R}=0.55$	11.3

The matched-privacy results show that the dynamically decaying mechanism yields a smaller economic penalty throughout the tested range. At $\bar{R}=0.67$, corresponding to the baseline dynamic case, the centralized cost gap decreases from 1.31% for fixed noise to 0.94%, a relative reduction of 28.2%. The same tendency is observed at the remaining matched-risk levels, for which the cost-gap reductions range from 33.0% to 40.2%. The matched-cost comparison gives the complementary result. At a cost gap of 0.94%, the mean leakage-risk score decreases from 0.72 for fixed noise to 0.67 for the dynamically decaying mechanism. Across the tested matched-cost levels, the reduction in the reconstruction-risk score ranges from 5.3% to 11.3%. These results do not indicate that dynamically decaying noise is uniformly more private than persistent fixed noise; rather, they show that, for the tested parameter range, the dynamic mechanism provides a more favorable balance when either empirical reconstruction risk or economic deviation is held approximately fixed.

#### Leakage evaluation under the inverse-variance estimator.

The same 720 day–hour communication records are evaluated using Eq (31) in addition to the original linear iteration weights. Table 9 summarizes the resulting mean leakage-risk scores and the proportions of high-risk periods. For the dynamically decaying mechanism, inverse-variance weighting increases the mean leakage-risk score from 0.67 to 0.75 and increases the proportion of high-risk periods from 43.75% to 56.94%. This result is consistent with the smaller late-iteration noise scales, because the variance-aware estimator assigns larger weights to the more weakly perturbed late-stage messages. For fixed Laplace noise, the inverse-variance rule reduces to uniform weighting because all $b_{k}$ are equal; the mean leakage-risk score changes from 0.58 to 0.61 and the high-risk proportion from 17.78% to 21.11%. In the unprotected case, the corresponding values increase slightly from 0.86 to 0.88 and from 82.36% to 84.17%, respectively. The stronger attack therefore reduces the apparent protection margin of all communication mechanisms, with the most pronounced increase occurring for the dynamically decaying sequence.

**Table 9:** Leakage-risk statistics under linear-weight and inverse-variance reconstruction.

Mechanism	Mean leakage risk		High-risk periods (%)	
	Linear	Inverse variance	Linear	Inverse variance
Without protection	0.86	0.88	82.36	84.17
Fixed Laplace noise	0.58	0.61	17.78	21.11
Dynamic Laplace noise	0.67	0.75	43.75	56.94

**Fig 34:**
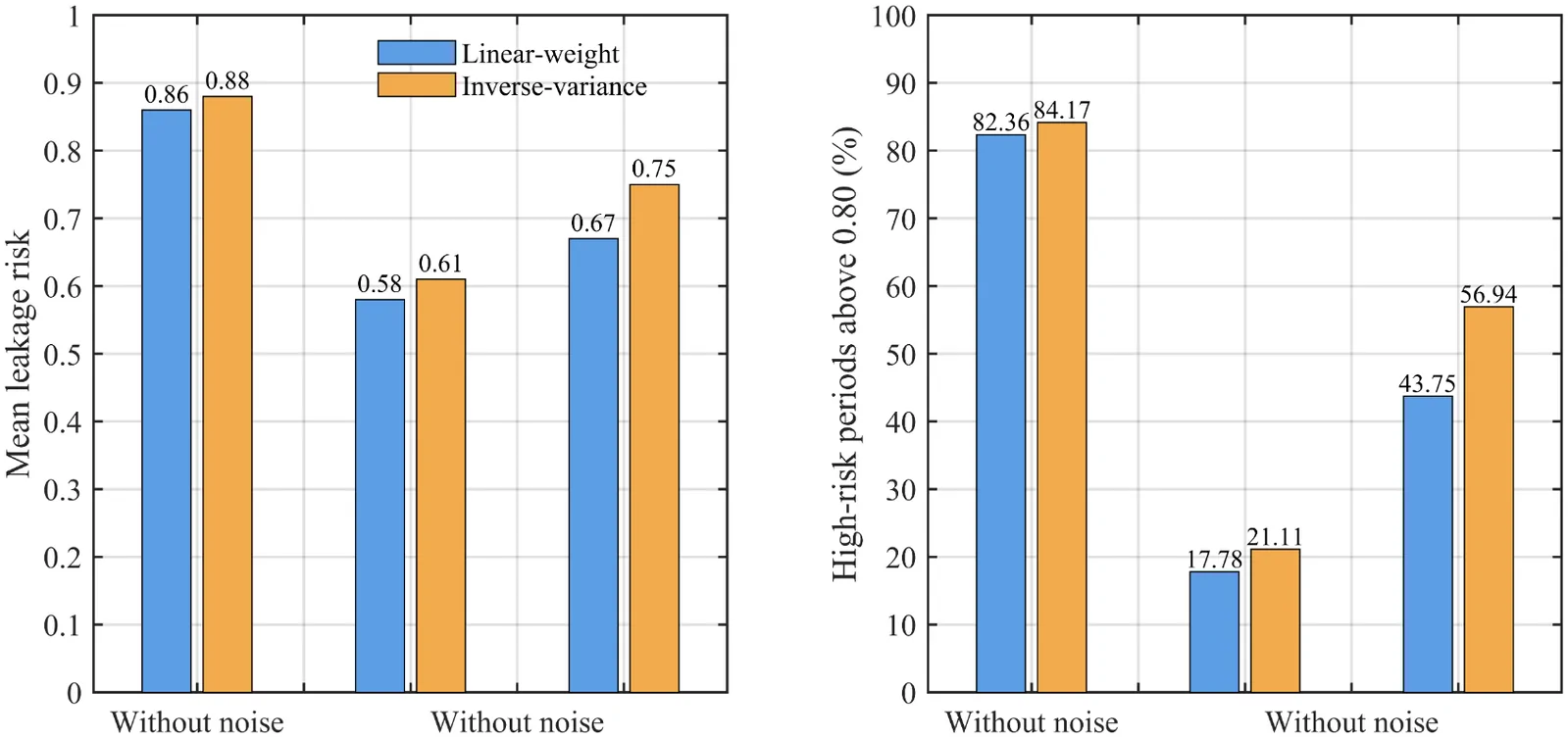
Reconstruction-based leakage risk under linear-weight and inverse-variance estimators.

Fig 34 provides the graphical comparison of the same statistics. The inverse-variance reconstruction is therefore treated as a stricter variance-aware stress test, without assuming that the original linear-weight estimator represents the strongest possible attack.

#### Offline terminal-set verification.

The numerical verification of the terminal-set construction follows the procedure stated after Assumption 2. The test set contains the eight vertices of the three-dimensional terminal box together with 1000 uniformly distributed interior states, giving 1008 terminal-state samples. For every sample, a one-step mixed-integer backup-feasibility problem is solved with the same physical and logic constraints used by the MPC. All 1008 samples admit a feasible backup action, and all successor states remain inside $X_{f}$. The maximum terminal-decrease residual in Eq (86) is $-2.1\times 10^{-3}$ in objective-function units; therefore, no positive terminal-decrease residual is observed in the sampled set. This sampled verification provides numerical evidence for the terminal conditions used in the nominal shifted-sequence argument. It is not interpreted as a proof of robust recursive feasibility under arbitrary disturbances.

#### Operational emissions and carbon-parameter sensitivity.

The base-case physical operational emissions calculated from Eq (13) are 2090.1 kg CO_2_ over the 24-h scheduling horizon. Direct gas-boiler emissions account for 705.0 kg, while indirect emissions associated with imported electricity account for 1385.1 kg. The corresponding imported-grid energy is 2430 kWh. A one-dimensional sensitivity test first varies $\mu_{Grid}$ from 0.456 to 0.684 kg CO_2_/kWh while retaining the nominal carbon price $\lambda_{CO2}=0.224$ CNY/kg. All other equipment, market, and privacy parameters remain unchanged. The numerical results are reported in Table 10.

**Table 10:** Sensitivity of operational emissions and cleared cost to $\mu_{Grid}$.

$\mu_{Grid}$ (kg CO_2_/kWh)	Grid import (kWh)	GB fuel input (kWh)	Operational CO_2_ (kg)	Cleared cost (CNY)
0.456	2570	3360	1850.6	12257.4
0.513	2505	3420	1975.9	12276.8
0.570	2430	3490	2090.1	12299.14
0.627	2365	3550	2200.0	12324.6
0.684	2300	3610	2302.4	12352.7

As $\mu_{Grid}$ increases, imported energy decreases from 2570 to 2300 kWh, while gas-boiler fuel input increases moderately from 3360 to 3610 kWh. The scheduling response therefore shifts part of the supply away from the increasingly carbon-intensive grid. The higher emission factor remains dominant in the evaluated physical total, so operational emissions increase from 1850.6 to 2302.4 kg and the cleared cost rises from 12257.4 to 12352.7 CNY over this slice.

**Fig 35:**
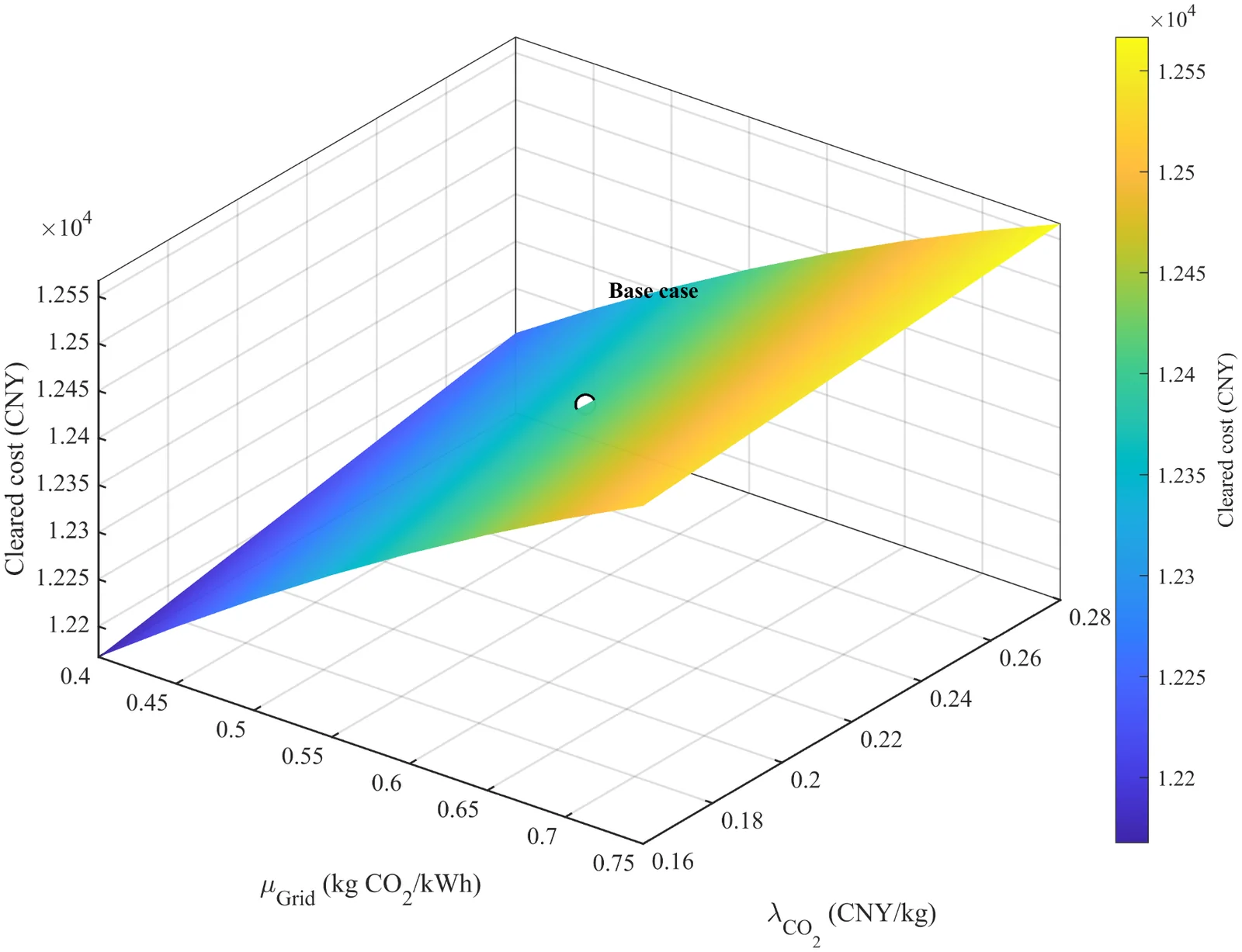
Joint sensitivity of cleared cost to the grid-import emission factor and carbon price.

Fig 35 extends the sensitivity test to the joint variation of the grid-import emission factor and carbon price. In the plotted range, $\mu_{Grid}$ varies from 0.40 to 0.75 kg CO_2_/kWh and $\lambda_{CO2}$ from 0.16 to 0.28 CNY/kg. The cleared-cost surface increases smoothly toward the region with larger $\mu_{Grid}$ and $\lambda_{CO2}$, with no abrupt change around the nominal operating point. This behavior is consistent with the carbon-cost formulation in Eq (12): a larger grid-emission factor increases the carbon responsibility assigned to imported electricity, while a larger carbon price increases the monetary weight of the resulting emission balance. The joint sensitivity result therefore confirms that both external carbon parameters affect the economic clearing result and that the scheduling response remains gradual over the investigated range.

## Conclusion

This paper develops a privacy-aware electricity–carbon scheduling framework that links day-ahead distributed clearing with intra-day MPC for a grid-connected hydrogen energy storage microgrid. The main findings are summarized as follows.

1) The electricity–carbon model accounts for direct gas-boiler emissions and indirect emissions from imported electricity while separating physical operational emissions from quota-based settlement. The base-case 24-h operational emissions are 2090.1 kg CO_2_. Increasing the grid-emission factor reduces imported energy but increases the evaluated total emissions and cleared cost over the tested range, while the joint $\mu_{Grid}$–$\lambda_{CO2}$ sensitivity remains smooth.
2) The dynamic Laplace mechanism is evaluated as a privacy–accuracy trade-off rather than as the strongest privacy option. The 38-iteration transcript has a basic-composition cumulative privacy budget of 60.23. Fixed noise gives a lower baseline mean leakage-risk score (0.58 versus 0.67), whereas the dynamic mechanism reduces the centralized cost gap from 2.85% to 0.94% and terminates in 38 rather than 50 iterations. Under matched privacy, the dynamic mechanism lowers the cost gap by 28.2%–40.2%; under matched cost, it lowers the mean leakage-risk score by 5.3%–11.3%. The inverse-variance estimator increases the dynamic-noise mean risk to 0.75, confirming the greater information content of late-stage releases.
3) The intra-day MPC uses a single control-reference tracking objective with a terminal storage-state condition. Offline verification over 1008 terminal-state samples finds feasible one-step backup actions for all tested samples, with a maximum terminal-decrease residual of $-2.1\times 10^{-3}$. The controller satisfies the stated nominal recursive-feasibility and stability conditions, while forecast-error tests show a gradual increase in operating-cost dispersion as uncertainty rises.

The present study assumes synchronized communication and a nominal MPC structure. Future work will consider delay-aware distributed updates, adaptive privacy-budget allocation, robust or stochastic MPC, and hardware-in-the-loop validation.

### Nomenclature

**Table:** 

Symbol	Definition	Unit
*t*,*k*	Scheduling-period and ADMM-iteration indices	–
$T,N_{p}$	Day-ahead scheduling periods and MPC prediction horizon	–
$P_{MG},P_{Grid}$	Signed microgrid and upper-level-grid tie-line powers; positive values denote import	kW
$P_{MG}^{imp},P_{MG}^{exp}$	Non-negative grid-import and grid-export components	kW
$P_{W},P_{PV}$	Wind- and photovoltaic-generation powers	kW
$P_{EL},P_{FC}$	Electrolyzer input and fuel-cell output powers	kW
$P_{bat}^{cha},P_{bat}^{dis}$	Battery charging and discharging powers	kW
$P_{EB},P_{GB}$	Electric-boiler electrical input and gas-boiler fuel input	kW
$Q_{tst}^{cha},Q_{tst}^{dis}$	Thermal-storage charging and discharging powers	${\text{kW}}_{th}$
$S_{SOC},S_{SOHC},S_{HOC}$	Battery, hydrogen, and thermal-storage normalized states	p.u.
$E_{bat}^{cap},E_{HSS}^{cap},E_{tst}^{cap}$	Battery, hydrogen, and thermal-storage capacities	kWh, ${\text{kg-H}}_{2}$, ${\text{kWh}}_{th}$
$\gamma_{EL},\gamma_{FC}$	Hydrogen production and consumption coefficients	${\text{kg-H}}_{2}$/kWh
$C_{grid},C_{OM},C_{fuel},C_{CO2}$	Grid transaction, maintenance, fuel, and carbon-trading costs	CNY
$\mu_{GB},\mu_{Grid}$	Gas-boiler and imported-grid emission intensities	kg CO_2_/kWh
$\lambda_{grid},\lambda_{gas},\lambda_{CO2}$	Electricity, natural-gas, and carbon prices	CNY/kWh, CNY/kg
*x*,*y*	Local decision vectors of the microgrid and upper-level grid	–
$\lambda^{k}$	ADMM dual multiplier vector	CNY/kW
ρ	ADMM augmented-Lagrangian penalty coefficient	–
$r^{k},s^{k}$	ADMM primal and dual residuals	kW
$\theta^{k},b_{k}$	Laplace-noise vector and scale at iteration *k*	kW
Δf	*L*_1_ global sensitivity of the released boundary-power vector	kW
$\epsilon_{0},\epsilon_{k},\epsilon_{cum}$	Baseline, per-release, and cumulative privacy budgets	–
c,α	Noise initialization coefficient and geometric decay factor	–
*x*(*k*),*u*(*k*),*w*(*k*)	MPC state, control, and disturbance vectors	–
*W*,*S*	Control-tracking and terminal-state weighting matrices	–
${𝒳}_{f},\kappa_{f}$	Terminal invariant set and terminal backup controller	–
$V_{f},\ell_{r}$	Terminal cost and rotated stage cost used in stability analysis	–
*M*	Big-*M* coefficient for mixed-integer logical constraints	kW
$\delta_{K}$	Bound on the terminal boundary-solution deviation from the centralized optimum	kW
